# Regio- and Stereoselective Synthesis of Nitro-fatty Acids as NRF2 Pathway Activators Working under Ambient or Hypoxic Conditions

**DOI:** 10.1021/acs.jmedchem.5c00982

**Published:** 2025-05-26

**Authors:** Daniel Chrenko, Jana Pereckova, Martina Zatloukalová, Jan Vacek, Jiří Pospíšil, Tomas Perecko

**Affiliations:** † Department of Chemical Biology, Faculty of Science, Palacky University, Šlechtitelů 27, CZ-783 71 Olomouc, Czech Republic; ‡ Department of Cell Biology and Radiobiology, Institute of Biophysics of the Czech Academy of Sciences, Kralovopolska 135, 612 00 Brno, Czech Republic; § Department of Medical Chemistry and Biochemistry, Faculty of Medicine and Dentistry, Palacky University, Hnevotinska 3, 775 15 Olomouc, Czech Republic; ∥ Department of Organic Chemistry, Faculty of Science, Palacky University, tř. 17. listopadu 1192/12, CZ-771 46 Olomouc, Czech Republic; ⊥ Laboratory of Growth Regulators, Palacky University & Institute of Experimental Botany AS CR, Šlechtitelů 27, CZ-783 71 Olomouc, Czech Republic

## Abstract

Nitro-fatty acids (NO_2_FAs) are endogenously produced electrophiles and NRF2 activators with therapeutic potential. We developed a synthetic protocol combining a Henry reaction and base-promoted β-elimination, yielding ultrapure regio/stereoisomers of nitro-stearic (NO_2_SA), nitro-oleic (NO_2_OA), and conjugated/*bis*-allylic nitro-linoleic (NO_2_LA) acids. These were tested for NRF2 pathway activation in bone marrow cells under different oxygen conditions. We observed that **9-** and **10-NO**_**2**_ **OA**, and **10-NO**_**2**_ **LA** increased NRF2 stabilization under hypoxia, while **9-** and **10-NO**_**2**_ **OA** significantly upregulated *Hmox1* and *Gclm* at all oxygen levels. **9-** and **10-NO**_**2**_ **OA** enhanced HO-1 and GCLM proteins independently of oxygen, while **10-NO**_**2**_ **LA** was oxygen-dependent, boosting HO-1 under hypoxia and GCLM under ambient conditions. Moreover, **10-NO**_**2**_ **OA** and **10-NO**_**2**_ **LA** induced NRF2 nuclear translocation. In contrast, the saturated **10-NO**_**2**_ **SA**, which has lower electron-acceptor ability, was inactive. In summary, these findings suggest the biological activity of NO_2_FAs is dependent on oxygen level, which could be used in future research of other oxidative stress-dependent pathways.

## Graphical abstract

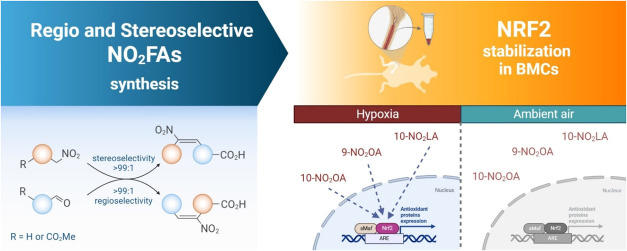

## Introduction

Nitro-fatty acids (NO_2_FAs) are a product of the reaction of reactive nitrogen species and unsaturated fatty acids (FAs), or their derivatives that can act as signaling mediators or transducers.^1,2^ The targeted covalent interactions of NO_2_FAs with amino acid residues: Cys and particularly also His, Lys and Ala, are closely related to the function of NO_2_FAs in cell signaling.^3^ NO_2_FAs are relatively strong electrophilic ligands,^4,5^ which generally react with nucleophiles or with the above-mentioned amino acid residues of proteins.^6,7^ These post-translational modifications are reversible, and for NO_2_FAs we refer to them as *S*-nitroalkylation.^3^ NO_2_FAs exhibit a pleiotropic mode of action^8^ and are broadly associated with cytoprotective, anti-inflammatory, immunomodulatory, and proliferative effects.^2^ NO_2_FAs specifically react with proteins that play a key role in redox homeostasis and the response to stress stimuli. The main cellular targets of NO_2_FAs include the nuclear factor erythroid 2-related factor 2 (NRF2) signaling pathway,^9^ NF-kappaB regulation^10^ or interaction with PPAR receptors,^11^ and STAT and MAPK regulation^12,13^ (reviewed in^14^). NO_2_FAs also modulate the activity of enzymes involved in redox homeostasis^15,16^ or, as was recently found, selectively react with the Ser/Thr phosphatase calcineurin, thereby modulating the production of pro-inflammatory cytokines.^17^

NO_2_FAs therefore represent a new group of electrophilic candidate molecules for drug development.^18^ One of the most important is nitro-oleic acid (NO_2_OA), which occurs in the form of two C9 and C10 isomers. The latter (CXA-10) and its derivatives are being considered as potential (leading) drug candidates.^19^ Other NO_2_FAs include *bis*-allylic nitro derivatives of linoleic acid, arachidonic acid or their conjugated versions, and other nitrated alkenes.^20,21^ In general, there are two different recognized synthetic approaches to NO_2_FAs: (a) biomimetic and (b) those based on a Henry reaction. For a recent overview of all synthetic sequences that yield NO_2_FAs, see^22^ and the Supporting Information. The biomimetic approach is straightforward, based on the transformation of FAs to the corresponding NO_2_FAs, in which a nitro-anion addition/elimination protocol is frequently used.^23–25^ The Henry reaction-based approach is a step-by-step synthesis, which usually involves a Henry reaction (C–C making coupling step) followed by the elimination of a (generally) modified hydroxy group.^22,25^

As was mentioned above, NO_2_FAs have been shown to target the NRF2 signaling pathway,^9,26^ while NRF2 plays a vital role in hematopoiesis.^27,28^ Hematopoietic stem and progenitor cells (HSPCs) reside in the bone marrow (BM) niche, an environment with lower oxygen tensions (1–4%) compared to atmospheric conditions (∼21%).^29^ This hypoxic microenvironment is crucial for maintaining HSPC self-renewal and quiescence, while increased levels of reactive species promote cellular proliferation and differentiation (reviewed in ref 30). However, the collection and processing of bone marrow cells (BMCs) are typically conducted in ambient air (∼21% O_2_), and even brief exposure to atmospheric air may result in changes in HSPCs.^31,32^ In this sense, BMCs are an oxygen-dependent *in vitro* cell model, which can also be used for the investigation of cytoprotective/radioprotective candidate molecules.

This work presents (a) a synthetic procedure for the selective preparation of regio/stereoisomers of NO_2_FAs with different and extended degrees of saturation. The modality of the synthetic approach is demonstrated by the preparation of saturated nitro-stearic acid (NO_2_SA), monounsaturated NO_2_OA and polyunsaturated nitro-linoleic acid (NO_2_LA) in conjugated and nonconjugated (*bis*-allylic) versions. (b) In the biological part of this work, a comparative study is presented that focuses on the use of the NO_2_FAs as activators of the NRF2 pathway in BMCs. (c) Since BMCs reside *in vivo* under hypoxic conditions, the effects of oxygen on NRF2 pathway activation are described here. This opens up an interpretative framework for the development of novel NRF2 activators that function under physiological oxygen tensions.

## Methods

### Chemical Synthesis

For a detailed description of the chemical procedures and compound characterization, see Supporting Information. All the prepared compounds were >95% pure by HPLC and ^1^H NMR analysis.

#### Experimental Procedures

##### General Procedure for Henry Reaction (Coupling of Nitroalkane **1** and Aldehyde **2**)

A neat mixture of nitroalkane **1** (1 mmol, 1.0 equiv) and aldehyde **2** (1.2 mmol, 1.2 equiv) was cooled to 0 °C and 1,1,3,3-tetramethylguanidine (0.2 mmol, 0.2 equiv) was added. The resulting mixture was stirred at room temperature for 12 h, cooled to 0 °C, and H_2_O (5 mL) was added. The resulting mixture was extracted with EtOAc (3 × 15 mL) and the combined organic phases were dried over Na_2_SO_4_, filtered and the solvents were removed under reduced pressure. The crude adduct **3** was purified by flash column chromatography.

##### General Procedure for Methyl Ester NO_2_FA Synthesis

Adduct **3** (0.66 mmol, 1.0 equiv) was dissolved in DCE (3.3 mL, 0.2M), and the resulting mixture was cooled to 0 °C. Et_3_N (1.98 mmol, 3.0 equiv) followed by trifluoroacetic anhydride (TFAA) (0.99 mmol, 1.5 equiv) were sequentially added. The whole mixture was stirred at 0 °C for 4 h and then at room temperature for the next 22 h. The whole mixture was cooled to 0 °C and H_2_O (10 mL) was added. The resulting mixture was extracted with EtOAc (3 × 15 mL), and the organic phases were combined, dried over MgSO_4_, filtered, and the volatiles were removed *in vacuo*. The crude product was purified by semipreparative HPLC chromatography (C18 reverse-phase column; MeOH:H_2_O).

##### General Procedure for Methyl NO_2_FA Ester Hydrolysis

CAL-B (400 mg) was added to a solution of methyl ester NO_2_FA in a mixture of acetone (11 mL, 0.08 M) and aqueous phosphate buffer (88 mL, 0.01 M, pH 7.4). The resulting solution was vigorously stirred (magnetic stirrer, 1000 rpm) at room temperature for 18 h, before the pH of the solution was adjusted to 3 with 1.0 M aq. HCl. The whole mixture was extracted with EtOAc (5 × 20 mL), and the combined organic layers were dried over MgSO_4_, filtered, and the volatiles were removed under reduced pressure. The crude product was purified by semipreparative HPLC chromatography (C18 reverse-phase column; MeOH:H_2_O).

##### General Procedure for Selective Nitro-olefin Function Reduction

Solid NaBH_4_ (1.5 mmol, 1.35 equiv) was added to a cold (0 °C, ice/water, external temperature) solution of the corresponding nitro-olefin compound (1.1 mmol, 1.0 equiv) in THF/MeOH (5.5 mL, 9:1 (*V/V*)). The cooling bath was removed, and the resulting mixture was stirred at RT for 14 h. H_2_O (5 mL) and EtOAc (5 mL) were added, and the resulting layers were separated. The aqueous layer was extracted with EtOAc (5 × 20 mL), and the combined organic layers were dried over MgSO_4_, filtered, and solvents were removed under reduced pressure. The crude product was purified by flash column chromatography on silica gel.

#### Characterization Data for Synthesized NO_2_FAs

##### 9-NO_2_OA

(0.1 g, 81%, *E/Z* ≥ 99:1 based on the ^1^H NMR spectra analysis). ^1^H NMR (500 MHz, Chloroform*-d*) δ 11.53 (broad s, 1H), 7.07 (t, *J* = 7.9 Hz, 1H), 2.60 – 2.51 (m, 2H), 2.34 (t, *J* = 7.5 Hz, 2H), 2.20 (q, *J* = 7.6 Hz, 2H), 1.62 (p, *J* = 7.4 Hz, 2H), 1.48 (h, *J* = 7.2 Hz, 4H), 1.37 – 1.29 (m, 6H), 1.32 – 1.21 (m, 10H), 0.92 – 0.83 (m, 3H); ^13^C {^1^H} NMR (126 MHz, Chloroform*-d*) δ 152.1, 136.4, 33.9, 32.0, 29.4, 29.32, 29.27, 29.1, 29.0, 28.6, 28.11, 28.05, 26.5, 24.7, 22.8, 14.2; HRMS (ESI, *m*/*z*) calculated for C_18_H_32_NO_4_^–^ [M-H]^−^ 326.2337, found 326.2326.

##### 10-NO_2_OA

(0.13 g; 45%, *E*/*Z* > 99:1 based on the ^1^H NMR spectra analysis). ^1^H NMR (500 MHz, Chloroform-*d*) δ 10.50 (broad s, 1H), 7.07 (t, *J* = 7.9 Hz, 1H), 2.59 – 2.54 (m, 2H), 2.36 (t, *J* = 7.5 Hz, 2H), 2.21 (q, *J* = 7.6 Hz, 2H), 1.67 – 1.60 (m, 2H), 1.53 – 1.44 (m, 4H), 1.39 – 1.32 (m, 6H), 1.32 – 1.24 (m, 10H), 0.88 (t, *J* = 6.8 Hz, 3H); ^13^C {^1^H} NMR (126 MHz, Chloroform-*d*) δ 152.1, 136.4, 33.8, 32.0, 29.4, 29.38, 29.32, 29.1, 29.0, 28.6, 28.12, 28.05, 26.5, 24.7, 22.8, 14.2; MS (ESI) *m*/*z* (%): 327 [M-H]^−^; HRMS (ESI, *m*/*z*) calculated for C_18_H_32_NO_4_^–^ [M-H]^−^ 326.2337, found 326.2340.

##### 10-NO_2_LA

(0.12 g, 42%, *E:Z/Z:Z* ≥ 99:1 based on the ^1^H NMR spectra analysis). ^1^H NMR (500 MHz, Chloroform*-d*) δ 11.14 (bs, 1H), 7.08 (t, *J* = 7.9 Hz, 1H), 5.49 (dtt, *J* = 10.9, 7.3, 1.8 Hz, 1H), 5.25 (dtt, *J* = 10.7, 6.9, 1.7 Hz, 1H), 3.34 (dd, *J* = 7.0, 1.8 Hz, 2H), 2.35 (t, *J* = 7.4 Hz, 2H), 2.24 (q, *J* = 7.6 Hz, 2H), 2.12 (q, *J* = 7.3 Hz, 2H), 1.65 – 1.61 (m, 2H), 1.53 – 1.47 (m, 2H), 1.38 – 1.29 (m, 12H), 0.90 (t, *J* = 6.8 Hz, 3H); ^13^C {^1^H} NMR (126 MHz, Chloroform*-d*) δ 179.6, 150.8, 136.6, 133.2, 123.4, 34.0, 31.7, 29.3, 29.2, 29.1, 29.0, 28.5, 28.1, 27.6, 25.0, 24.7, 22.7, 14.2; MS (ESI, *m*/*z*) (%): 324 [M-H]^−^ (100); HRMS (ESI, *m*/*z*) calculated for C_18_H_30_NO_4_^–^ [M-H]^−^ 324.2169, found 324.2179.

##### 9-NO_2_cLA

(0.44 g, 42%, *E/Z* ≥ 99:1 based on the ^1^H NMR spectra analysis). ^1^H NMR (500 MHz, Chloroform*-d*) δ 7.56 – 7.52 (m, 1H), 6.35 (dt, *J* = 14.5, 7.0 Hz, 1H), 6.19 (ddt, *J* = 15.0, 11.4, 1.4 Hz, 1H), 2.69 – 2.62 (m, 2H), 2.35 (t, *J* = 7.5 Hz, 2H), 2.27 – 2.22 (m, 2H), 1.63 (p, *J* = 7.9, 7.1 Hz, 2H), 1.52 (d, *J* = 7.3 Hz, 2H), 1.48 – 1.42 (m, 2H), 1.37 – 1.25 (m, 12H), 0.94 – 0.85 (m, 3H); ^13^C {^1^H} NMR (126 MHz, Chloroform*-d*) δ 178.3, 149.5, 149.2, 134.1, 123.7, 33.83, 33.79, 31.8, 29.12, 29.06, 29.04, 28.99, 28.7, 28.2, 26.7, 24.7, 22.7, 14.2; HRMS (ESI, *m*/*z*) calculated for C_18_H_30_NO_4_^–^ [M-H]^−^ 324.2180, found 324.2178.

##### 10-NO_2_SA

(0.17 g, 96%) in the form of colorless oil. ^1^H NMR (500 MHz, Chloroform*-d*) δ 11.15 (bs, 1H), 4.45 (tt, *J* = 9.3, 4.5 Hz, 1H), 2.34 (t, *J* = 7.5 Hz, 2H), 1.99 – 1.89 (m, 2H), 1.72 – 1.58 (m, 4H), 1.36 – 1.22 (m, 22H), 0.87 (t, *J* = 7.0 Hz, 3H); ^13^C {^1^H} NMR (126 MHz, Chloroform*-d*) δ 179.7, 89.2, 34.1, 34.0, 31.9, 29.4, 29.3, 29.19, 29.17, 29.11, 29.06, 29.02, 25.93, 25.90, 24.7, 22.8, 14.2; MS (ESI, *m*/*z*) 329 [M-H]^−^; HRMS (ESI, *m*/*z*) calculated for C_18_H_34_NO_4_^–^ [M-H]^−^ 328.2493, found 328.2496.

### Compound Storage

All the prepared compounds were placed in vials and stored at −80 °C under an argon atmosphere in their neat form. They proved to be stable over a six-month period. Before use, the compounds were brought up to room temperature (RT) and 10 mM stock solutions in DMSO were prepared. These DMSO stock solutions were stored at −20 °C under an argon atmosphere.

### Ethics Approval

All experiments using animal cells were conducted in compliance with EU Directive 2010/63/EU and were approved by the Ethical Committee of the Academy of Sciences of the Czech Republic (AVCR 1823/2023 SOV II).

### Isolation and Culture of Primary Cells

Primary bone marrow cells (BMCs) were isolated from 8–12-week-old male C57BL/6 mice (25–30 g, AnLab, Czech Republic). Mice were housed under standard conditions. BMCs were isolated as follows: The day before isolation, phosphate-buffered saline (PBS), ACK Lysis Buffer (A10492-01, Thermo Fisher Scientific), and Iscove’s Modified Dulbecco’s Medium (IMDM) (12440-053, Thermo Fisher Scientific) were placed in a hypoxic chamber (InvivO2 400, Baker Ruskinn, UK) with adjustable oxygen control to equilibrate for the target oxygen concentrations. On the day of the experiment, animals were euthanized using ketamine/xylazine, and bilateral femurs were extracted. BMCs were flushed with hypoxic (1% O_2_ unless stated otherwise) PBS supplemented with 2% fetal bovine serum (FBS; low endotoxin, FB-1101/500, Biosera, France). After hemolysis at room temperature using hypoxic ACK Lysis Buffer, nucleated BMCs were washed with hypoxic PBS + 2% FBS and counted using a TC20 automated cell counter (Bio-Rad). Cells were kept under hypoxic conditions prior to treatment. The medium for BMC culture, consisting of IMDM with 2% FBS and 1% penicillin/streptomycin (LM-A4118/100, Biosera, France), was supplemented with 10 ng/mL mouse IL-3, 10 ng/mL mouse IL-6, and 50 ng/mL mouse stem cell factor - SCF (213-13, 216-16, and 250-03, respectively; all from PeproTech). Cells were cultured at 37 °C in a humidified atmosphere containing 5% CO_2_ and the specified oxygen concentrations (21%, 4%, or 1%).

### Cytotoxicity Assay

For cytotoxicity experiments, mouse embryonic fibroblasts (MEFs; STO, 86032003, Sigma-Aldrich) were cultured in DMEM (41966-029, Thermo Fisher Scientific) + 5% FBS and were seeded at 10,000 cells per well in a 96-well plate and allowed to adhere for 8 h under atmospheric conditions. Plates were then placed in incubators with the specified oxygen concentrations (21%, 4%, or 1%) and incubated overnight. The following day, the medium was replaced with fresh medium (+5% FBS) adjusted to match the target oxygen concentrations and containing the test compounds (1–100 μM). After 24 h of incubation, adherent cells were stained using a modified sulforhodamine B (SRB) assay as described in.^33^ SRB was obtained from Sigma-Aldrich (S1402). Briefly, the medium was aspirated, and cells were fixed with 10% cold trichloroacetic acid. After washing twice with distilled water, the cells were stained with 0.1% SRB dissolved in 1% acetic acid. The cells were then washed 3–4 times with 1% acetic acid and allowed to dry. The stain was solubilized with 10 mM unbuffered TRIS base, and absorbance was measured at 540 nm (Infinite M200 Pro, Tecan, Austria). The cell viability of treated samples was calculated as a percentage of nontreated controls, and IC_50_ values were determined (see Statistics). According to the IC_50_ values, a nontoxic 2 μM concentration of NO_2_FAs was used in further *in vitro* experiments. For detailed results, see Supporting Information Figure S1 and Table S3.

### Gene Expression

For the relative gene expression of nuclear factor erythroid 2-related factor 2 (NRF2, encoded by *Nfe2l2*), heme oxygenase-1 (*Hmox1*), and Glutamate-Cysteine Ligase Modifier Subunit (*Gclm*) (Mm00477784_m1, Mm00516005_m1, Mm00514996_m1, all from Thermo Fisher Scientific), mouse BMC (3 × 10^6^ cells) were cultivated on a 6-well plate in 1.5 mL of complete media (see Isolation and culture of primary cells), and then incubated with 2 μM NO_2_FAs or control (medium only) for 4 h at the specified oxygen levels (21%, 4%, or 1%). Then, the adherent and nonadherent cells were washed with PBS and lysed using a NucleoSpin RNA Plus kit (740984, Machery-Nagel; Germany). The concentration and purity of isolated RNA was assessed using an Infinite M200 Pro spectrophotometer (Tecan, Austria). Total RNA (0.2 μg) was reversely transcribed into first strand cDNA using a RevertAid First Strand cDNA Synthesis kit according to the manufacturer’s protocol (K1622, Thermo Fisher Scientific). qRT-PCR reactions were performed in a LightCycler480 instrument (Roche; Switzerland) using TaqMan FastAdvanced Master Mix for qPCR (4444557, Thermo Fisher Scientific) and the following program was used: uracil-*N*-glycosylase activation step at 50 °C for 2 min, an initial denaturation step at 95 °C for 2 min, followed by 45 cycles (95 °C for 3 s, 60 °C for 30 s), and a final cooling step at 40 °C for 1 min. Data were normalized to ribosomal protein L32 (*Rpl32*, Mm02528467_g1, Thermo Fisher Scientific) and presented as 2^–Δcq^.

### Western Blot

Briefly, 1.5 × 10^6^ BMCs were placed in 0.2 mL of complete medium in 1.5 mL microtubes and incubated with 2 μM NO_2_FAs or the control (medium only) for 1 (NRF2), 4 (heme oxygenase-1, HO-1), or 7 (glutamate-cysteine ligase modifier subunit, GCLM) hours at the specified oxygen levels (21%, 4%, or 1%). Cells were then spun down and washed with PBS (from this step onward, cells were processed under atmospheric conditions). Next, cells were lysed in 60 μL RIPA buffer (89901, Thermo Fisher Scientific) containing protease and phosphatase inhibitors cocktail (78442, Thermo Fisher Scientific). Cell lysates were sonicated and centrifuged at 15,000*g* for 3 min at 4 °C, and 15 μL of lane marker (3900, Thermo Fisher Scientific) was added to the supernatants. Samples were boiled for 3 min at 95 °C. Equal volumes of lysates (15–45 μL/well) were loaded onto 7–15% SDS-polyacrylamide gels. Proteins were then transferred onto PVDF membranes (IPVH85R, EMD Millipore Corp.) and blocked with 5% nonfat dry milk in TBST. Membranes were incubated overnight at 4 °C with the following primary antibodies: anti-β-actin (1:1000; #3700, Cell Signaling Technology), antivinculin (1:10,000; #SAV9264, Sigma-Aldrich), anti-NRF2 (1:500; A308758, Lot 38410, Antibodies.com, UK), anti-HO-1 (1:1000; #43966, Cell Signaling Technology), antihistone H3 (1:2000; #4499, Cell Signaling Technology), or anti-GCLM (1:100; sc-55586, Santa Cruz Biotechnology). This was followed by incubation with corresponding HRP-linked secondary antibodies (antimouse IgG, #7076, 1:1000–5000; or antirabbit IgG, #7074, 1:1000–2000; both from Cell Signaling Technology) for 1 h at room temperature. Protein bands were visualized using an enhanced chemiluminescent substrate (SuperSignal 34577/34095, Thermo Fisher Scientific). The optical densities of detected proteins were measured using ImageJ v1.52 (NIH).

For the NRF2 translocation assays, 5.5 × 10^6^ BMCs were incubated in 0.5 mL of complete medium with 2 μM NO_2_FAs or the control for 1 h in 1.5 mL microtubes, but only at 1% oxygen. Cytosol, membrane and nuclear cell compartments were isolated with a Cell Fractionation Kit (9038, Cell Signaling Technology) according to the manufacturer’s instructions. Equal volumes of nuclear lysates (20 μL/well) were loaded onto 7–15% SDS-polyacrylamide gels. Histone H3 was used as the loading control for the nuclear fraction.

### Immunocytochemistry

BMCs (2 × 10^6^ cells in 300 μL of complete medium) were incubated with **10-NO**_**2**_ **OA** or control medium for 1 h in a 1% oxygen incubator. Cells were then washed with PBS, resuspended in fresh PBS, and plated on an 8-well μ-slide (ibidi, Germany). After allowing cells to settle (10 min), PBS was aspirated, and cells were fixed with 4% paraformaldehyde for 10 min. From this step onward, all work was performed under atmospheric conditions. Cells were washed twice, permeabilized with 0.1% Triton X-100, washed again, and blocked with 1% BSA/PBS. Samples were incubated with primary anti-NRF2 antibody (1:100, SAB4501384, Millipore) overnight at 4 °C. After washing, a secondary Alexa Fluor 647 antibody (1:100, A31573, Invitrogen) was added for 1 h at room temperature in the dark. After washes, nuclei were stained with DAPI (0.5 μg/mL, Merck) for 10 min at room temperature in the dark, washed, and mounted on a microscope slide. Images were acquired with an Olympus FV10i (Olympus, Japan) scanning microscope and analyzed using ImageJ v1.52 (NIH).

### Statistics

Values are presented as arithmetic means ± standard deviations (SD), unless otherwise specified. Half-maximal inhibitory concentration (IC_50_) values were calculated using four-parameter logistic regression (dose vs response). For Western blot analyses, one-way ANOVA with Dunnett’s multiple comparisons test was performed against control samples. The significance level was set at *p* < 0.05. Statistical analyses were conducted using GraphPad Prism v10.4 (GraphPad Software, www.graphpad.com). Statistical methods and the number of experiments are indicated in the figure legends.

## Results

### Chemistry

To successfully accomplish the synthesis of NO_2_FA, a two-step approach that included a C–C bond-forming Henry reaction (Figure 1A, regioselectivity control) and a base-promoted β-elimination process (Figure 1B, stereoselectivity control) was proposed. In the first step, the Henry reaction, a simple functional group switch should enable us to prepare possible NO_2_FA isomers (NO_2_-group location) from essentially the same starting material. The second step, base-promoted β-elimination, should further establish the required *E* stereochemistry. We expected that by properly choosing the activation agent (transforming the β-hydroxy group into a good leaving group) and base, *in situ* epimerization of a stereogenic center adjacent to the nitro group would drive the formation of the *E* olefin.

**Figure 1:**
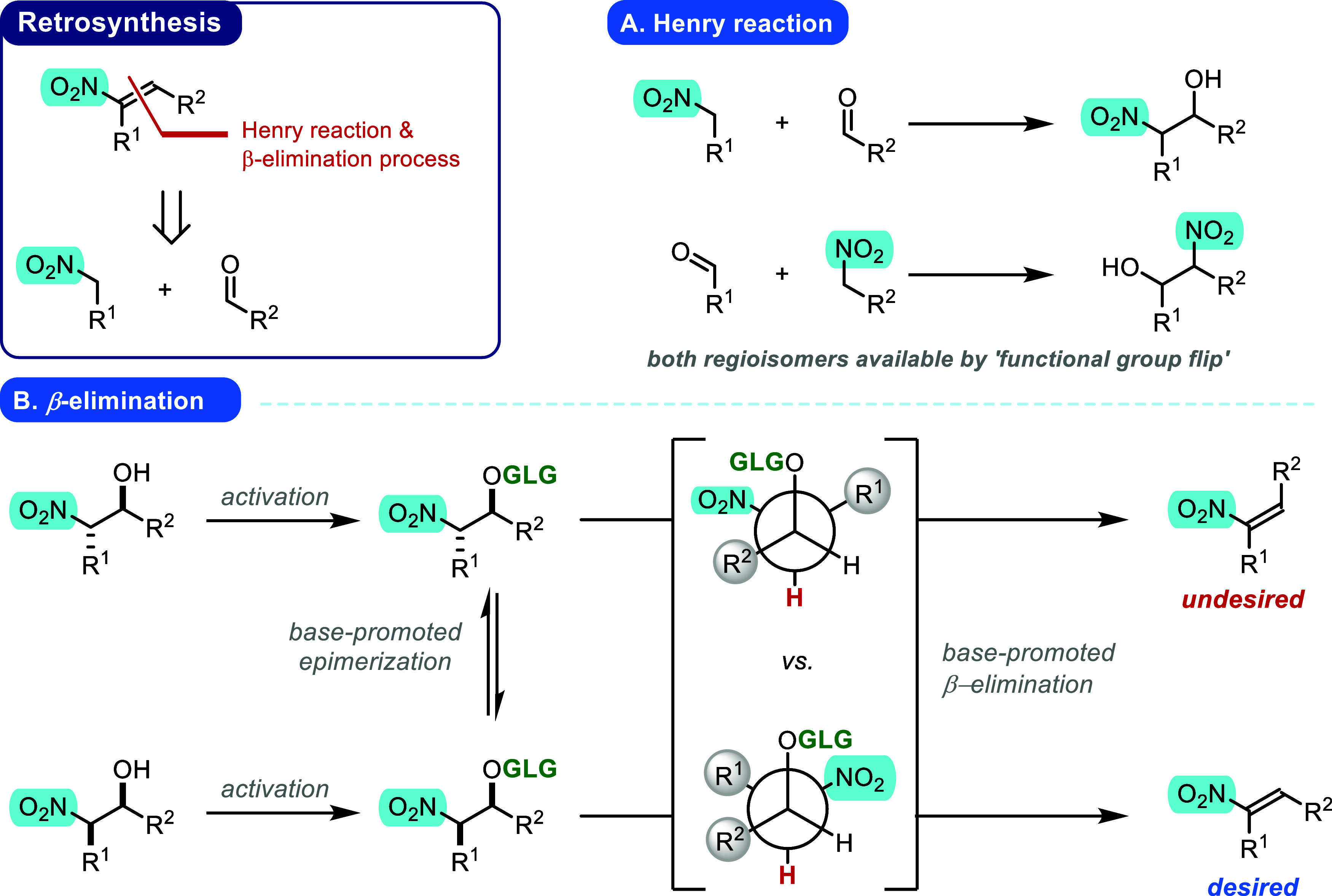
Retrosynthesis of nitro-olefins. (A) Henry reaction: control over regioisomer synthesis. (B) β-Elimination: activator and base pair-controlled stereoselective nitro-olefin synthesis. *GLG = good leaving group, R* ^1^ *and R* ^2^ *= alkyl chains.*

To validate our approach, the synthesis of **9-NO**_**2**_ **OA** and **10-NO**_**2**_ **OA** was attempted (Scheme 1). We identified aldehyde **2a** as a common building block for the synthesis of both **NO**_**2**_ **OA**s, since it can be directly used as one of the two coupling partners in the synthesis of **10-NO**_**2**_ **OA**. Additionally, its three-step transformation could generate nitro alkane **1b**, a coupling partner required for the synthesis of **9-NO**_**2**_ **OA**.

**Scheme 1:**
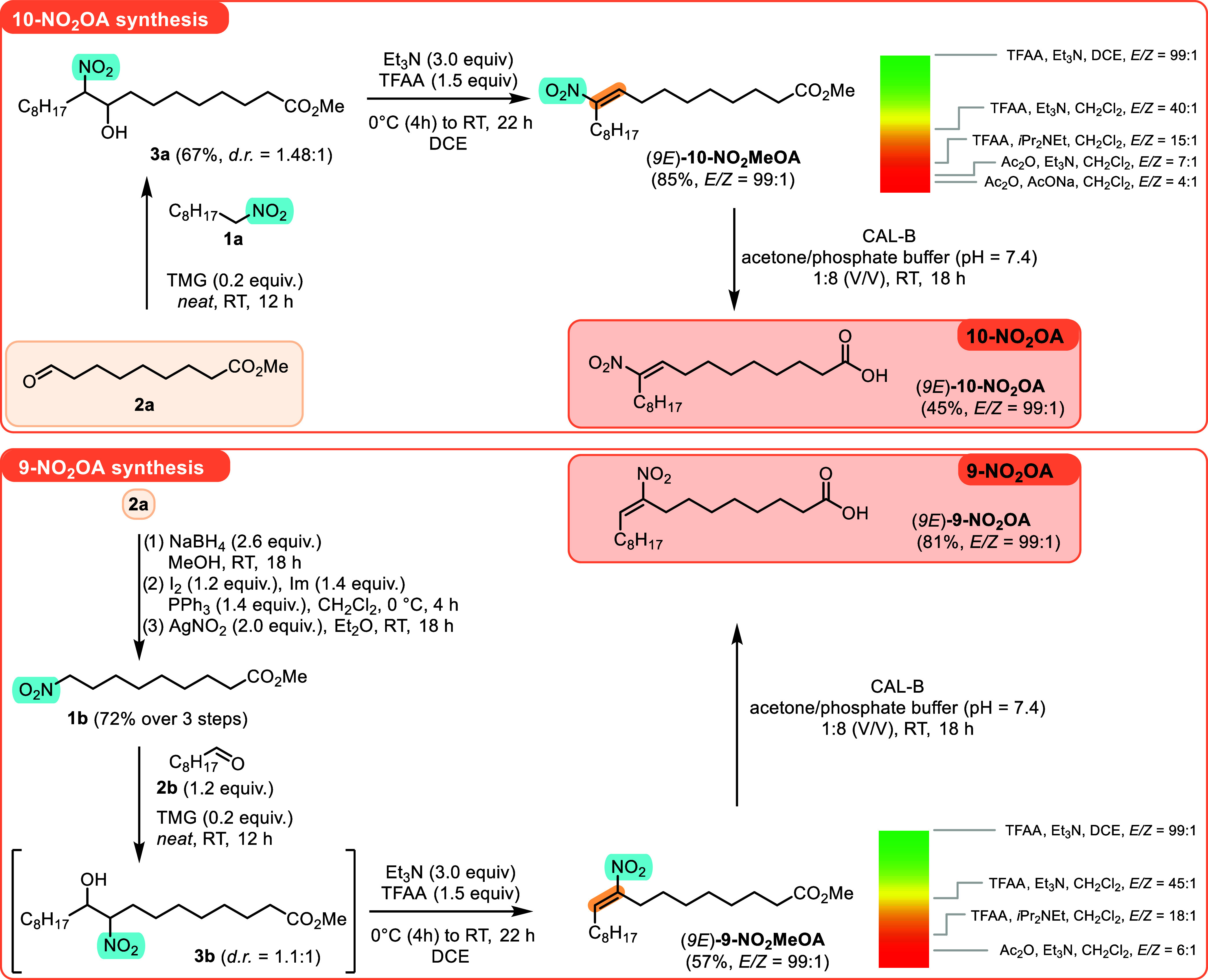
Stereo- and Regioselective Synthesis of (9*E*)-9- and (9*E*)-10-Nitro-oleic Acids (**9-NO**_**2**_ **OA** and **10-NO**_**2**_ **OA**)­^a^

a All yields correspond to isolated material. For more details on how the activation agent/base pair affects the *E*/*Z* ratio during olefin formation, see Supporting Information. The *E*/*Z* and diastereoisomer (d.r.) ratios are based on the crude reaction mixture ^1^H NMR spectra analysis. *CAL-B = Candida Antartica Lipase B, DCE = 1,2-dichloroethane, TFAA = trifluoroacetic anhydride, TMG = 1,1,3,3-tetramethylguanidine*.

The synthesis of **10-NO**_**2**_ **OA** began by combining nitro alkane **1a** with aldehyde **2a** in the presence of 20 mol % of 1,1,3,3-tetramethylguanidine (TMG). The solvent-free Henry reaction yielded the desired product **3a** as an inseparable mixture of two diastereoisomers (*d.r.* = 1.48:1) in a 67% isolated yield. With easy access to the desired adduct **3a**, we evaluated the key elimination step. It was found that using an acetyl group to activate the **3a** hydroxy group, in combination with bases like AcONa or Et_3_N, generated the desired olefin but with only moderate *E* selectivity. Detailed analysis revealed that the formation of the desired olefin was accompanied by the retro-Henry reaction (aldehyde **2a** and traces of nitro alkane **1a** were detected). We expected that the presence of the nitro alkane **3a** anion, generated *in situ* by the retro-Henry reaction, was accelerating the elimination step (the presence of a strong base avoided equilibration between both isomers, as expected in Figure 1B).

To avoid the undesired retro-Henry reaction, we used a stronger acylating agent, trifluoroacetic anhydride (TFAA), to activate the hydroxy group in **3a**. Gratifyingly, the use of TFAA in combination with *i*Pr_2_NEt or Et_3_N yielded the desired olefin with increased *E* selectivity (it is expected that the lower *E* selectivity observed with *i*Pr_2_NEt, being a sterically more demanding base, is caused by a slower reprotonation step in the base-triggered equilibration step). Finally, changing the solvent from CH_2_Cl_2_ to DCE led to the formation of (9*E*)-**10-NO**_**2**_ **MeOA** in an 85% isolated yield and with 99:1 *E/Z* selectivity.

Having paved the way to a single isomer of (9*E*)-**10-NO**_**2**_ **MeOA**, ester hydrolysis was attempted. Standard reaction conditions resulted in low reaction yields of **10-NO**_**2**_ **OA** and often compromised the stereointegrity of both the starting material **3a** and the desired acid **10-NO**_**2**_ **OA**. Gratifyingly, the use of *Candida antarctica* lipase B (CAL-B)^34^ solved the problem, yielding the desired **10-NO**_**2**_ **OA** in a 45% yield and 99:1 *E/Z* selectivity.

We then applied the developed synthetic approach to the synthesis of **9-NO**_**2**_ **OA** (Scheme 1). First, aldehyde **2a**, a common starting material, was transformed into nitro alkane **1b** in three steps with a 72% overall yield. Next, the Henry reaction of **1b** with nonanal **2b** in the presence of TMG (20 mol %) yielded the adduct **3b** as a mixture of two diastereoisomers (*d.r.* = 1.1:1). The crude product was used directly in the TFAA/Et_3_N-mediated elimination step, and the desired olefin (9*E*)-**9-NO**_**2**_ **MeOA** was obtained in a 57% yield as a virtually single *E* isomer (*E/Z* = 99:1). Enzymatic methyl ester hydrolysis finally yielded the desired **9-NO**_**2**_ **OA** in an 81% isolated yield and a 99:1 *E/Z* ratio.

Having established a stereoselective and efficient approach to the synthesis of **NO**_**2**_ **OA**, the synthesis of **10-NO**_**2**_ **LA** (*bis*-allylic linoleic acid) and **9-NO**_**2**_ **cLA** (conjugated linoleic acid) was attempted (Scheme 2). In the first case, the synthesis was straightforward, and the previously developed approach consisting of the Henry reaction/elimination/methyl ester hydrolysis sequence could be readily applied. The only challenge in the synthesis was related to the synthesis of nitro alkene **1c**. Even though the transformation of silyl homopropargyl ether to the corresponding alcohol **4**, which comprises a sequence of three steps (alkylation/Nickel catalyst-promoted *cis*-hydrogenation/desilylation), was straightforward, the further transformation of alcohol **4** to nitro alkene **1c** surprised us with its exclusivity. First, it was envisaged that alcohol **4** would be transformed into the corresponding nitro alkene via an alkenyl bromine intermediate. The choice of alkenyl bromide as the reaction intermediate was motivated by the concern that the synthesis of the corresponding iodo intermediate might be accompanied by iodine-mediated *Z* to *E* olefin isomerization. Unfortunately, the use of alkyl bromide as a reaction intermediate yielded the desired nitroalkene as an inseparable mixture of nitro- and nitritoalkenes. In contrast, alkyl iodine yielded nitroalkene **1c** exclusively, and no isomerization of the olefin bond was observed under the applied reaction conditions.

**Scheme 2:**
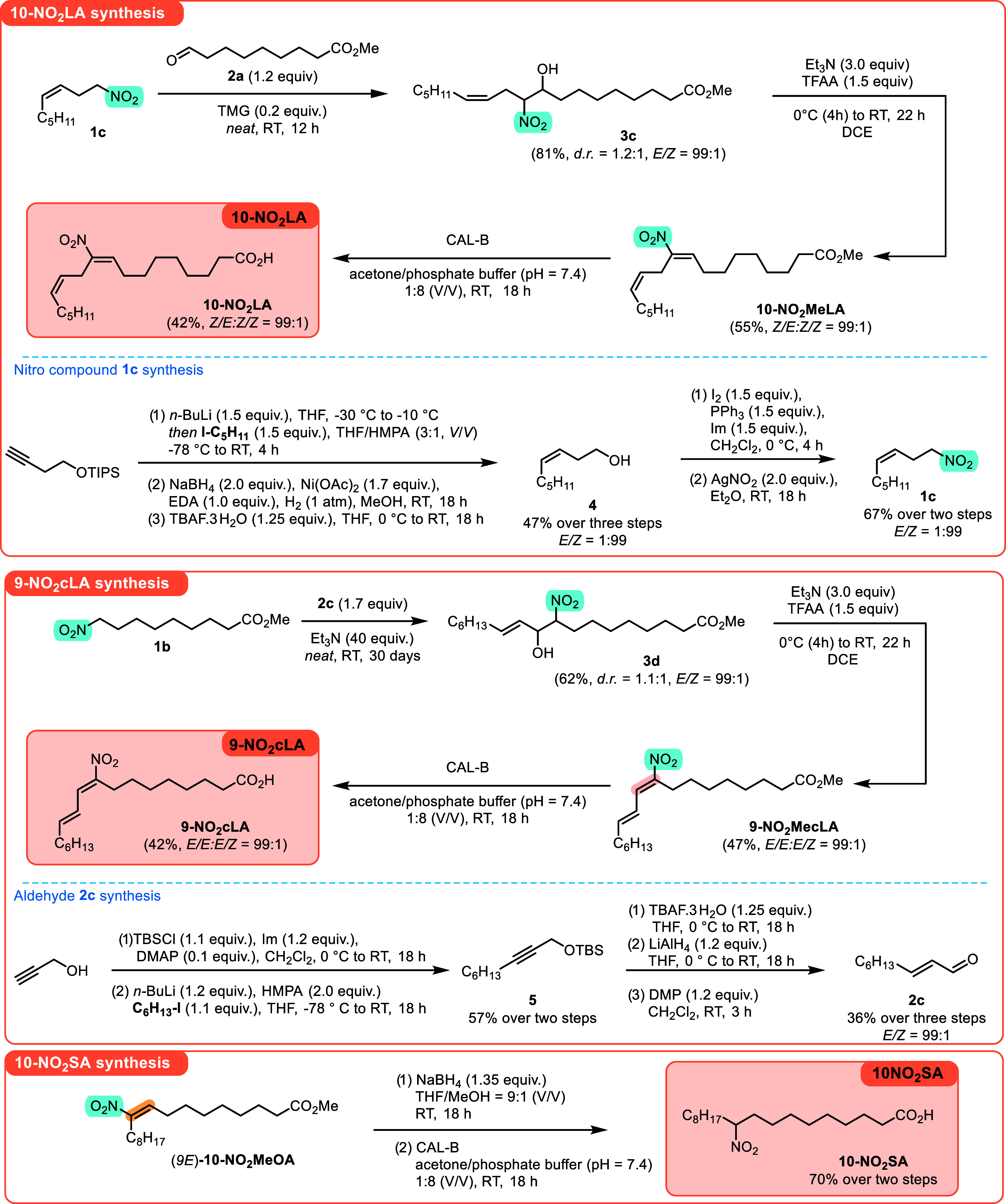
Stereo- and Regioselective Synthesis of (9*E*,12*Z*)-10-Nitro Linoleic Acid (**10-NO**_**2**_ **LA**), (9*E*,11*E*)-9-Nitro Conjugated Linoleic Acid (**9-NO**_**2**_ **cLA**), and 10-Nitro Stearic acid (**10-NO**_**2**_ **SA**)­^a^

a All yields correspond to isolated material. The *E*/*Z* and diastereoisomer (d.r.) ratios are based on the crude reaction mixture ^1^H NMR spectra analysis. *CAL-B = Candida antarctica lipase B, DCE = 1,2-dichloroethane, DMAP = 4-dimethylaminopyridine, DMP = Dess–Martin periodinane, EDA = ethylenediamine, HMPA = hexamethylphosphoramide, Im = imidazole, TBAF = tetra-n-butylammonium fluoride, TBSCl = tert-butyldimethylsilyl chloride, TFAA = trifluoroacetic anhydride, TMG = 1,1,3,3-tetramethylguanidine*.

In the **9-NO**_**2**_ **cLA** synthesis, the Henry reaction coupling step had to be adapted, as previously developed reaction conditions proved to be inefficient. Finally, the use of modified literature conditions,^35^ with changes in reaction time and equivalents, led to the desired adduct **3d**. Unfortunately, even under these reaction conditions (reaction time extended to 30 days), only a partial conversion of nitro alkane **1b** to the adduct was observed.

Finally, a **10-NO**_**2**_ **OA** analog without the olefinic bond, **10-NO**_**2**_ **SA**, was prepared starting from the (9*E*)-**10-NO**_**2**_ **MeOA** ester. Indeed, chemoselective reduction of the double bond in the presence of the carboxylic ester function was carried out. The resulting methyl ester was then hydrolyzed to the corresponding acid with the help of CAL-B.

### Effects of NO_2_FAs on NRF2 Stabilization under Different Oxygen Conditions

Different chemoprotective agents were shown to induce a rapid stabilization of NRF2 protein within minutes after incubation.^36^ Based on this relatively fast effect and our preliminary data, we analyzed the stabilization of NRF2 in BMCs after 1 h of incubation with a 2 μM concentration of the tested compounds under different oxygen conditions, emphasizing a more physiological environment. As shown in Figure 2, the effects of NO_2_FAs on NRF2 stabilization were structure and oxygen dependent. Despite the fact that the NRF2 protein was stabilized under atmospheric conditions, the addition of NO_2_FA derivatives did not further enhance its expression (Figure 2A). On the other hand, the incubation of BMCs with NO_2_FAs at 4 and 1% oxygen led to an increased stabilization of NRF2 compared to the corresponding control (Figure 2B,C). Of the derivatives tested, **10-NO**_**2**_ **LA** significantly increased the expression of NRF2 at 4% oxygen (Figure 2B), and **9-NO**_**2**_ **OA** and **10-NO**_**2**_ **OA** significantly increased the expression of NRF2 at 1% oxygen (Figure 2C). **9-NO**_**2**_ **cLA** only produced a nonsignificant increase at both 4 and 1% oxygen. On the other hand, the saturated derivative **10-NO**_**2**_ **SA** produced no effect under all three oxygen conditions (Figure 2A–C). Based on the above results and with respect to physiological oxygen concentrations in bone marrow, we performed further analyses under hypoxic conditions. For the selected results, comparison with atmospheric conditions were performed.

**Figure 2:**
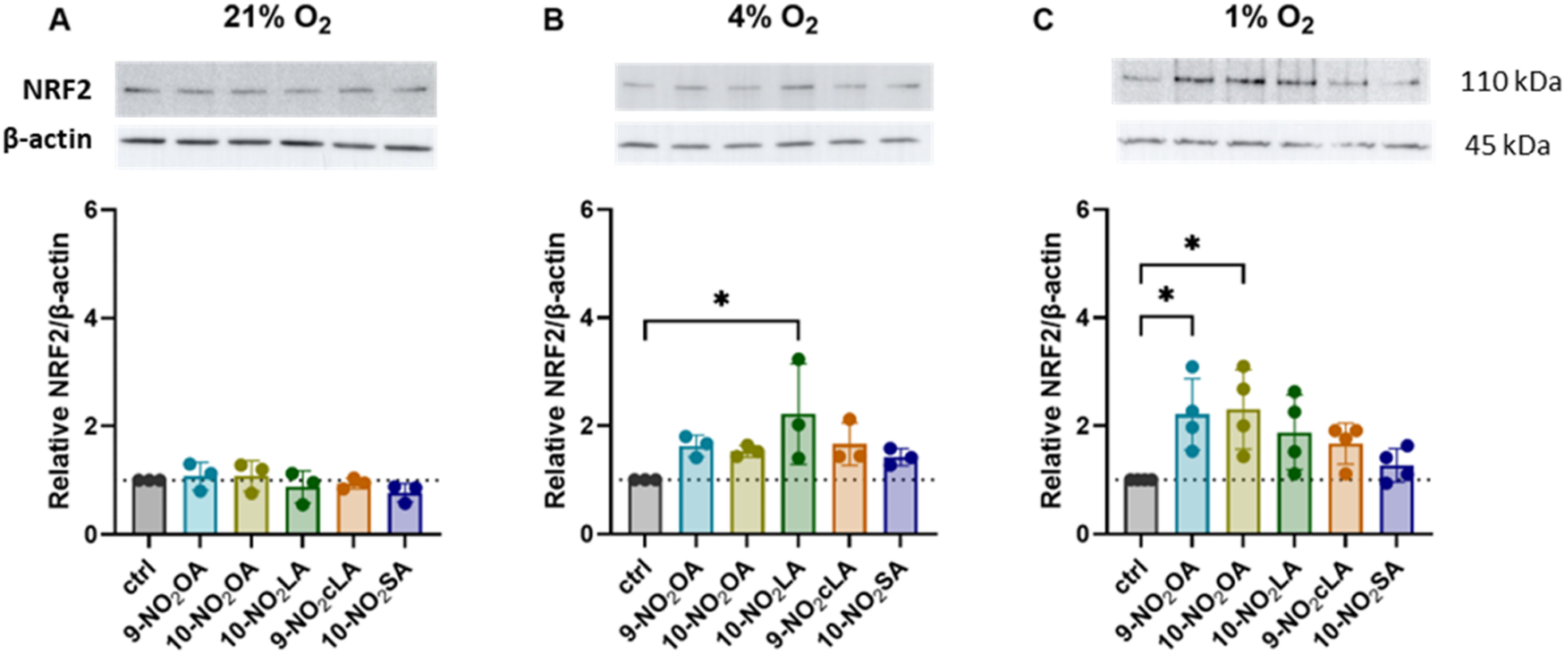
Stabilization of NRF2 by tested NO_2_FA derivatives. *NO*_*2*_ *FA derivatives stabilize NRF2 in mouse bone marrow cells (BMCs). Nucleated BMCs were cultured under varying oxygen conditions (21%, 4%, and 1%) and treated for 1 h with 2 μM of the tested NO*_*2*_ FAs, followed by analysis of NRF2 expression in whole-cell lysates. Results are shown as the fold change relative to control cells across different oxygen culture conditions (A–C). Data are expressed as mean ± SD from 3–4 independent experiments (n = 3–4). Statistical analysis was performed using one-way ANOVA with Dunnett’s multiple comparisons test (* p < 0.05).

### Expression of *Nfe2l2* and NRF2-Target Genes

We evaluated the expression of the NRF2 gene (encoded by *Nfe2l2*) and its target genes (*Hmox1* and *Gclm*) under 4% and 1% oxygen conditions. As shown in Figure 3A, the tested compounds did not alter *Nfe2l2* gene expression after 4 h of incubation, except for **9-NO**_**2**_ **OA**, which significantly increased *Nfe2l2* expression in 4% oxygen. In contrast, both NRF2-responsive genes *Hmox1* and *Gclm* were significantly upregulated after incubation with the tested NO_2_FAs (Figure 3B,C). Specifically, **9-NO**_**2**_ **OA** and **10-NO**_**2**_ **OA** produced significant upregulation across different oxygen levels in both genes, whereas **9-NO**_**2**_ **cLA** or **10-NO**_**2**_ **LA** exhibited upregulation that was dependent on oxygen levels or specific target genes, respectively. On the other hand, **10-NO**_**2**_ **SA** had no effects on *Nfe2l2, Hmox1* and *Gclm* under both oxygen conditions. Dimethyl fumarate was added as a control (Figure 3). Notably, when analyzing the expression of *Hmox1* across varying oxygen conditions, including atmospheric levels, **10-NO**_**2**_ **OA** significantly upregulated the expression of *Hmox1* at 1% oxygen compared to 21% (Supporting Information Figure S2A). This effect, however, was not observed for *Gclm* (Supporting Information Figure S2B).

**Figure 3:**
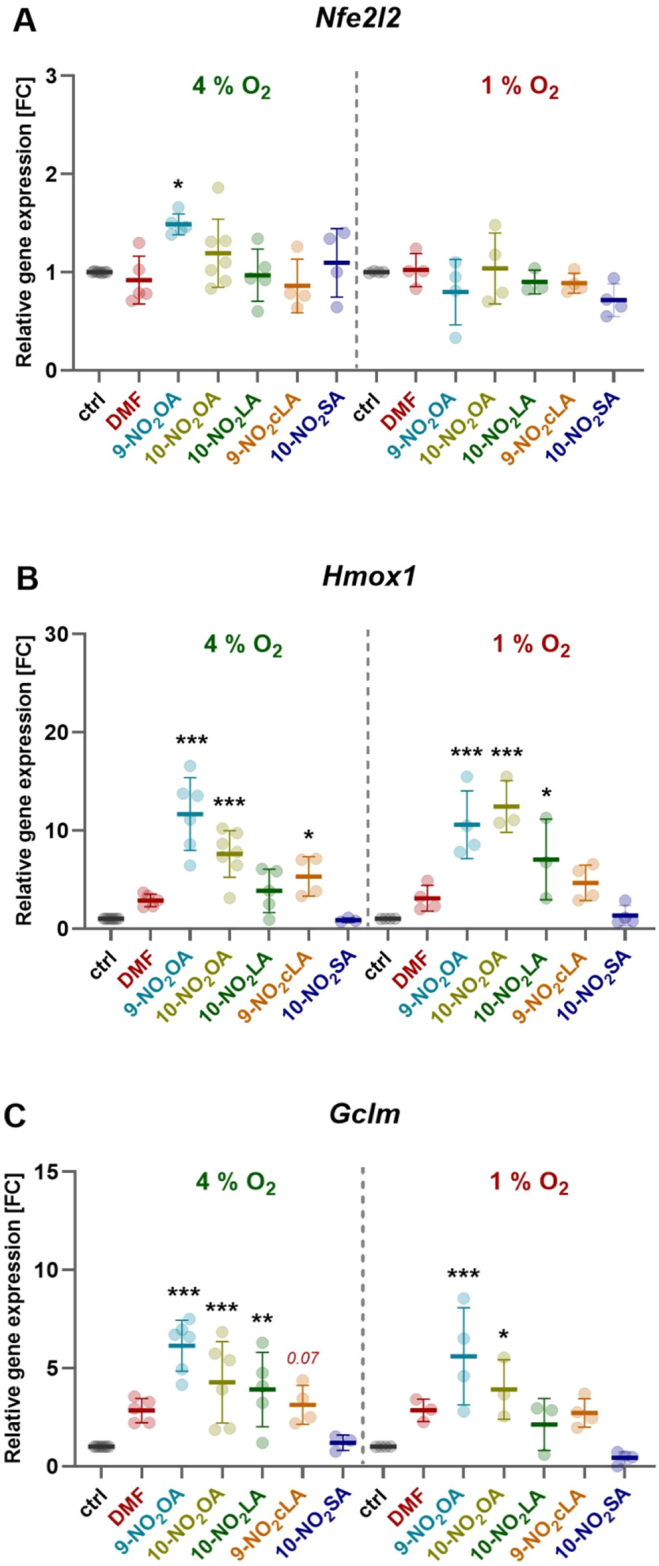
Expression of NRF2 and its target genes in response to NO_2_FA derivatives. *BMCs were cultured under 4% or 1% oxygen conditions and treated for 4 h with the tested NO*_*2*_ *FAs (2 μM) or medium followed by quantification of (A) Nfe2l2, (B) Hmox1, and (C) Gclm with PCR. Results are presented as relative expression across different oxygen culture conditions. Dimethyl fumarate (DMF)* was added as a control. Data are expressed as mean ± SD from 3 to 7 independent experiments (*n* = 3–7), analyzed with one-way ANOVA with Dunnett’s multiple comparisons test compared to the controls (*p < 0.05; **p < 0.01; ***p < 0.001).

### Effects of NO_2_FAs on NRF2-Target Protein Level and Nuclear Translocation

To further test the NRF2 stabilization and activation, we analyzed the level of NRF2-target proteins in BMCs. We selected HO-1 and GCLM, which were also significantly upregulated at the gene level (above). The protein level of HO-1 was evaluated after 4 h of incubation, and GCLM was evaluated after 7 h of incubation. Under atmospheric conditions, **9-NO**_**2**_ **OA** and **10-NO**_**2**_ **OA** significantly increased HO-1 levels (Figure 4A). At 1% oxygen, these effects were roughly 1.8-fold and 2.2-fold higher, respectively, while **10-NO**_**2**_ **LA** only increased HO-1 under hypoxic conditions (Figure 4B).

**Figure 4:**
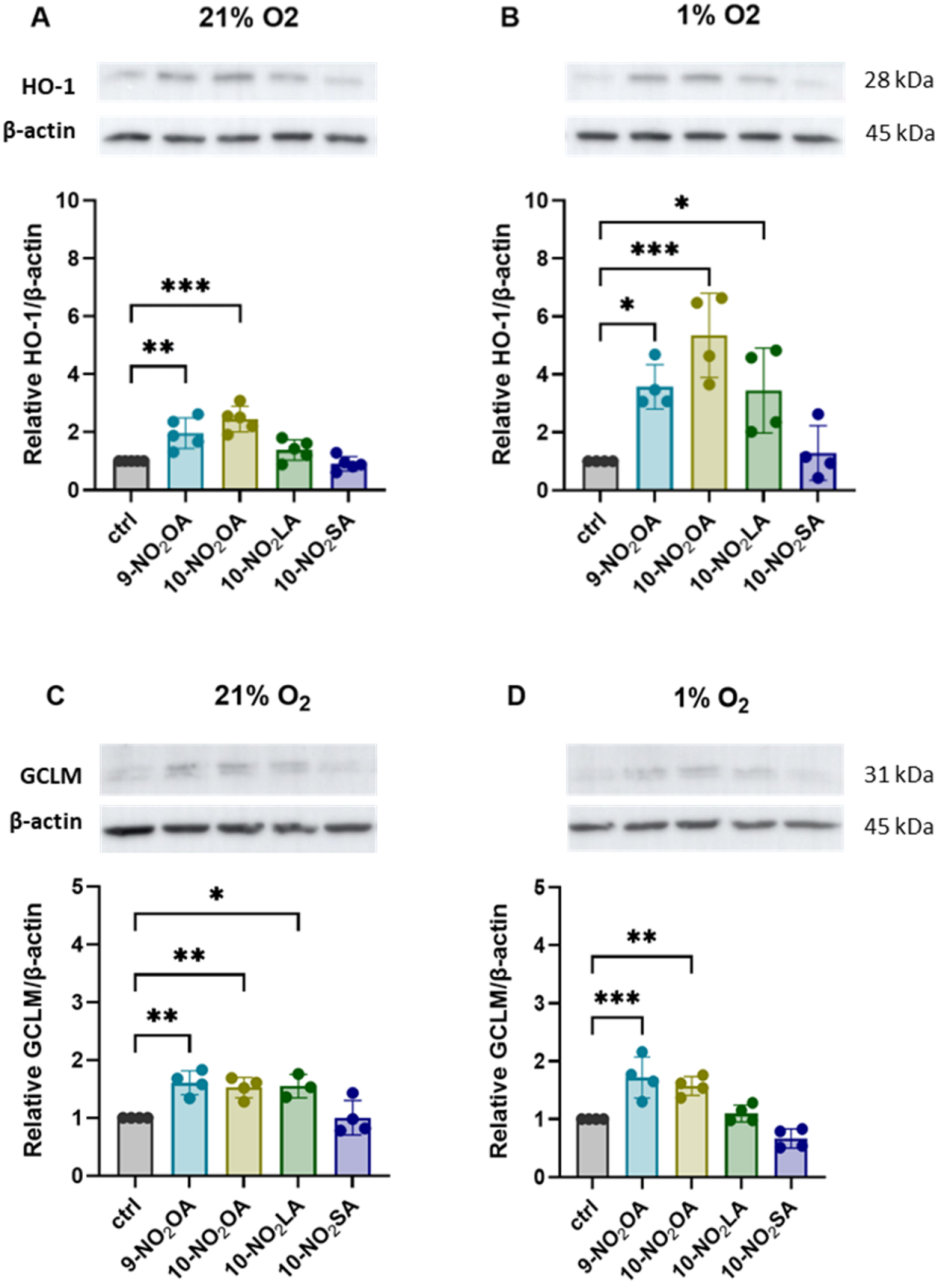
Expression of NRF2-target proteins HO-1 and GCLM. *NO*_*2*_ *FA derivatives upregulate the expression of NRF2-target proteins in mouse BMCs. BMCs were cultured under 21% or 1% oxygen conditions and treated with 2 μM of the tested NO*_*2*_ *FAs for 4 h (A, B) or 7 h (C, D). HO-1 (A, B) and GCLM (C, D) expression in whole-cell lysates was subsequently analyzed. Results are presented as the fold change relative to control cells across different oxygen culture conditions.* Data are expressed as mean ± SD from 3–5 independent experiments (*n* = 3–5). Statistical analysis was performed using one-way ANOVA with Dunnett’s multiple comparisons test (*p < 0.05; **p < 0.01; ***p < 0.001).

On the other hand, the protein level of GCLM in BMCs showed nearly the same increase after incubation with **9-NO**_**2**_ **OA** or **10-NO**_**2**_ **OA** in either 21% or 1% oxygen. The effect of **10-NO**_**2**_ **LA** was only significant at 21% oxygen (Figure 4C,D). As in previous experiments, the saturated derivative **10-NO**_**2**_ **SA** had no significant effects at either oxygen level on both evaluated proteins (Figure 4A–D).

Based on the above results, we next analyzed the translocation of NRF2 protein to the nucleus in BMCs. We only selected the 1% oxygen cultivation and the two most promising compounds. Because **9-NO**_**2**_ **OA** or **10-NO**_**2**_ **OA** exhibited similar effects, we chose **10-NO**_**2**_ **OA** and **10-NO**_**2**_ **LA** for the comparison of NRF2 translocation. As shown in Figure 5A, both tested compounds significantly increased the translocation of NRF2 to the nucleus, with **10-NO**_**2**_ **OA** producing a ca. 1.7-fold higher activity than **10-NO**_**2**_ **LA**. **10-NO**_**2**_ **SA** had no effects on NRF2 translocation. Similarly, by immunocytochemistry we observed an increased colocalization of NRF2 with the nucleus after the treatment of BMCs with **10-NO**_**2**_ **OA** (Figure 5B).

**Figure 5:**
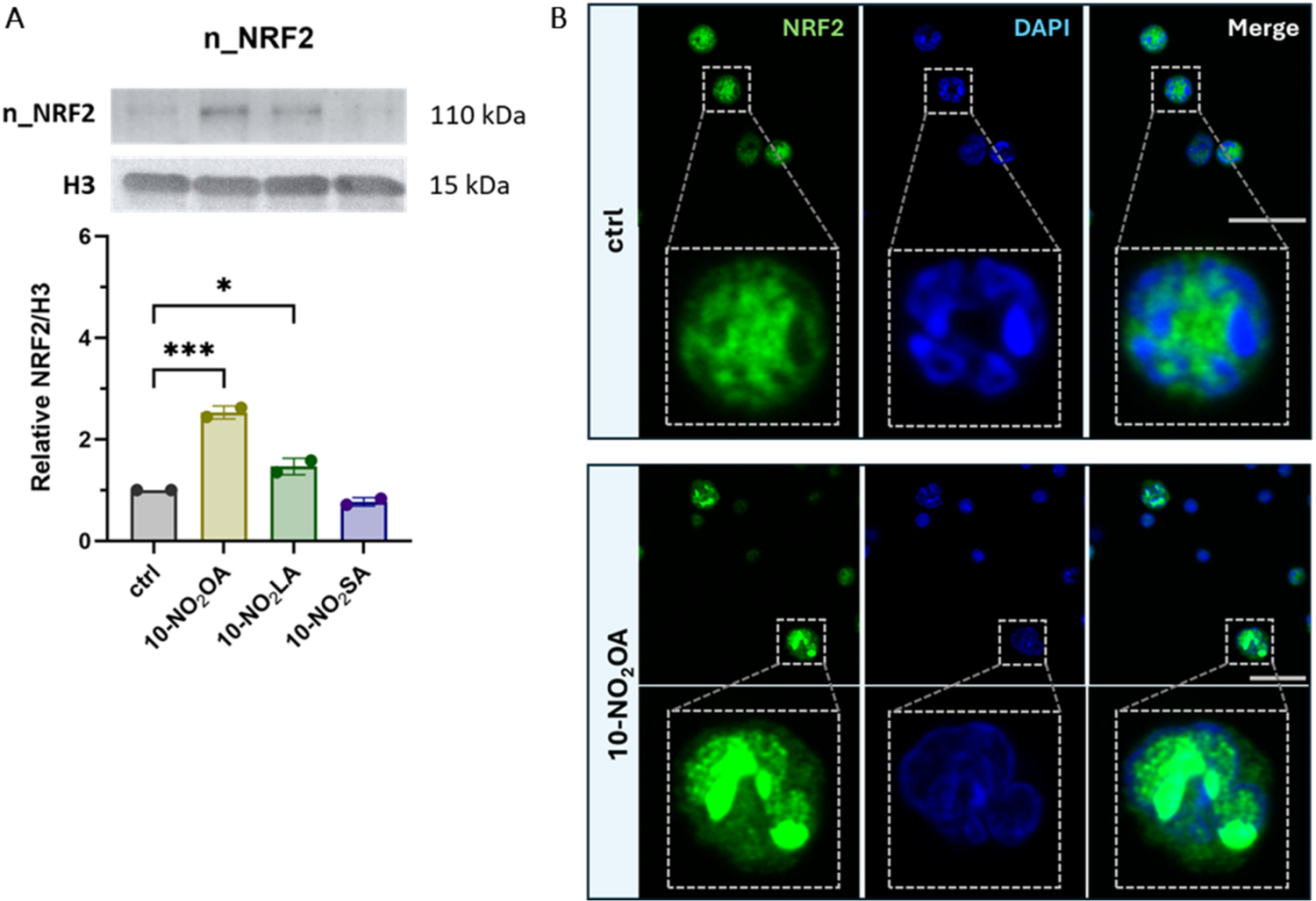
Subcellular localization of NRF2. *NO*_*2*_ *FA derivatives promote nuclear translocation of NRF2 in mouse BMCs. BMCs were cultured under 1% oxygen conditions and treated for 1 h with 2 μM of 10-NO*_*2*_ *OA, 10-NO*_*2*_ *LA, or 10-NO*_*2*_ SA. A cell fractionation kit was used to isolate the nuclear fraction (A), followed by the detection of NRF2 and histone H3 (used as a control nuclear protein). Western blot results (A) are shown as the fold change relative to control cells. Data are expressed as mean ± SD from two independent experiments (n = 2). Statistical analysis was performed using one-way ANOVA with Dunnett’s multiple comparisons test (* p < 0.05; *** p < 0.001). (B) Representative immunocytochemistry staining of BMCs with NRF2 antibody. BMCs were cultured in 1% oxygen followed by NRF2 and nuclei (DAPI) staining. Treatment with 10-NO_2_OA increased the colocalization of NRF2 with the nucleus (scale bar: 20 μm).

## Discussion

*Synthetic work.* Although there are several methods for the stereoselective synthesis of specific NO_2_FAs,^35,37,38^ most studies related to biological activity use mixtures of NO_2_FAs (both regio- and stereoisomers).^23,25^ For a detailed discussion about the synthetic approaches to NO_2_FAs, see Supporting Information. These mixtures are generated through biomimetic, nonstereo- and nonregioselective synthesis based on the addition of reactive nitrogen species to FAs. The developed stereoselective approaches then rely either on the use of a final-stage heavy-metal-catalyzed reaction step (NO_2_OA and NO_2_LA^39^ – possible heavy-metal contamination) or use a strong Lewis acid in the final step of the synthesis (NO_2_OA^38^ and cLA^35^ conditions were not applied to skipped diene systems as in NO_2_LA; possible olefin migration to thermodynamically stable conjugated diene was expected).

Since our goal was to apply stereo- and regio-defined NO_2_FAs in our experiments, we envisaged an efficient approach to synthesizing these molecules using a Henry reaction (coupling step, Figure 1A) followed by stereoselective elimination (Figure 1B). The choice of substrates in the Henry reaction (nitroalkane **1** and aldehyde **2**) determines the regioselectivity of the reaction (position of the NO_2_ group in NO_2_FAs). The reaction conditions applied during β-elimination (desired *E* olefin generation via a proper combination of acylating agent – activation of the β-hydroxy group; and a base – equilibration of the activated Henry adducts to the desired syn-intermediate) determine the stereochemistry of the reaction. This sequence of steps (Scheme 1 and Scheme 2) yielded the desired methyl ester of the targeted NO_2_FAs. At this stage, a selective lipase-mediated methyl ester hydrolysis was employed to yield the desired NO_2_FAs in good to very good yields. The enzymatic approach was selected due to its mild reaction conditions (pH = 7.4; room temperature), which avoid any undesired side reactions related to olefin migration and/or isomerization.

Using this approach, we achieved a short, efficient, selective, and heavy-metal-free synthesis of **9-NO**_**2**_ **OA** (6 steps, 33.3% overall yield), **10-NO**_**2**_ **OA** (4 steps, 18.5% overall yield), **10-NO**_**2**_ **LA** (9 steps, 7.6% overall yield), and **9-NO**_**2**_ ***c*** **LA** (10 steps, 2.5% overall yield). The reaction yields are competitive and, in some cases, even superior to previously published stereo- and regioselective heavy-metal-free methods (**9-NO**_**2**_ **OA**, 7.2% over 4 steps;^38^ **10-NO**_**2**_ **OA**, 23% over 5 steps;^38^ **10-NO**_**2**_ **LA**, 0.6% over 10 steps;^40^ **9-NO**_**2**_ **cLA**, 2.7% over 10 steps^35^). All prepared NO_2_FAs were prepared as single regioisomer and with >99:1 double bond stereoselectivity.

### Limitations and Further Directions

In principle, the developed method should enable us to prepare any NO_2_FA, considering the position of the NO_2_ group within the double bond(s) system of NO_2_FAs in both regio- and stereoselective manners. However, although the method itself is not problematic, the stability of the starting materials under basic conditions (especially readily enolizable aldehydes) hinders e.g., the stereoselective synthesis of **9-NO**_**2**_ **LA**. Enolization and subsequent olefin isomerization/migration in such aldehydes occurs readily under the coupling (Henry reaction) conditions. Despite substrate limitations, no further restrictions of our method are expected. To validate our approach, the stereo and regioselective synthesis of more complex NO_2_FAs, e.g., NO_2_-arachidonic acid,^41^ will be investigated in the future.

#### NRF2 Signaling Pathway

NO_2_FAs have been shown to target the NRF2 signaling pathway.^9,26^ As a master regulator of the antioxidant response, NRF2 plays a crucial role in hematopoiesis by controlling the differentiation,^27^ quiescence and self-renewal of HSPCs,^28^ and supporting their survival^42^ as well as migration, homing, and expansion (reviewed in^43^). This central role makes NRF2 a potential therapeutic target under conditions of oxidative stress.

HSPCs reside in a low-oxygen (1–4%) BM niche,^29^ however, BMC processing is generally carried out in ambient air (∼21% O_2_), exposing cells to hyperoxic conditions that may induce oxygen stress.^32^ To address these discrepancies, we investigated the effects of NO_2_FAs on the NRF2 pathway in mouse BMCs across a range of oxygen concentrations. Importantly, both ambient and hypoxic conditions have been reported to promote NRF2 stabilization; however, the cell type may have an impact on these effects.^44,45^ In our experiments, NRF2 protein stabilization in BMCs by NO_2_FAs was observed at 4% and 1% oxygen, but not at 21% oxygen. As for structure–activity relationships, **9-NO**_**2**_ **OA** and **10-NO**_**2**_ **OA** were more effective at 1% oxygen than NO_2_LA (in both unconjugated and conjugated forms) or the saturated stearic acid derivative. At 4% oxygen, only **10-NO**_**2**_ **LA** exhibited a significant effect. Our results confirm that NO_2_OA ranks among the most effective NO_2_FAs, with both positional isomers exhibiting similar efficacy.

Information on the effects of NO_2_FAs on NRF2 protein stabilization is limited. A few studies have directly examined NRF2 nuclear translocation via Western blot analysis. For example, a mixture of 9/10-NO_2_OA was used to induce NRF2 nuclear translocation in cancerous or endothelial and smooth muscle cells.^46,47^ In another study, the investigation of NO_2_LA positional isomers revealed NRF2 stabilization and nuclear translocation.^48^ Extending the acyl chain generally enhances NRF2 stabilization until the length exceeds 18 carbons—at which point efficacy diminishes—whereas short-chain NO_2_FAs are also less effective; notably, **9-NO**_**2**_ **OA** induced NRF2 more robustly than either shorter or longer chain derivatives in a colon cancer cell line.^49^ At 1% oxygen, our analysis of NRF2 nuclear translocation in BMCs shows that **10-NO**_**2**_ **OA** is more effective than **10-NO**_**2**_ **LA**, aligning with the findings of Bates et al. under ambient conditions.^46^

In contrast to these protein-level analyses, antioxidant response element (ARE)-reporter luciferase assays are used to evaluate NRF2 activation. However, NO_2_FAs were reported to interact with the luciferase enzyme.^49^ Interestingly, **9-NO**_**2**_ **OA** preferentially activated NRF2 over **10-NO**_**2**_ **OA**,^26^ highlighting the importance of distinguishing between these distinct isomers when evaluating NRF2 activation. A broader structure–activity study found that NO_2_FAs with longer carbon chains produced a more robust increase in ARE activity than **10-NO**_**2**_ **OA**.^50^

To further support NRF2 transcriptional activation, the expression of NRF2-target genes, e.g., HO-1 or GCLM, are used as surrogate markers. However, these proteins can be regulated by multiple transcription factors besides NRF2.^51^ In our study, we analyzed the NO_2_FAs-induced gene expression of *Nfel2l, Hmox1*, and *Gclm*, as well as the protein levels of HO-1 and GCLM, under varying oxygen concentrations. Consistent with previous reports,^9,47^ we observed no significant increase in *Nfel2l* expression except for a modest effect of **9-NO**_**2**_ **OA** at 4% oxygen. On the other hand, *Hmox1* and *Gclm* were significantly upregulated under different oxygen conditions, with the strongest effects seen for **9-** and **10-NO**_**2**_ **OA**, as well as **10-NO**_**2**_ **LA**. Supporting our findings, Khoo et al.^50^ reported that **10-NO_2_OA** induced a higher expression of *Gclm* and *Hmox1* compared to other derivatives. At the protein level, NO_2_FA-induced HO-1 expression was higher at 1% oxygen than under ambient conditions. Exposure to hypoxia increases the expression of HO-1,^52^ while GCLM levels remained relatively consistent across different oxygen tensions.^53^ Overall, both NO_2_OA isomers demonstrated similar activity, but **10-NO**_**2**_ **OA** exhibited greater potency at inducing HO-1.

*Limitations and Further Directions*: Nevertheless, some limitations of our study should be acknowledged. First, although bulk BMCs are a heterogeneous mixture of cells, our experiments did not allow us to determine which specific cell populations contributed to the observed effects. As a result, extrapolating these findings to the entire hematopoietic system remains challenging, and future studies should include more defined cell populations to better assess the effects. Second, the absence of experiments using *NRF2* knockout cell lines limits our ability to definitively attribute the observed effects solely to NRF2 activation. Third, *in vivo* studies using mouse models were not conducted and should be performed in future.

However, our previous work demonstrated that mixed-isomer NO_2_OA can mitigate hematopoietic acute radiation syndrome in sublethally irradiated mice,^54^ in line with earlier reports suggesting that targeting the NRF2 pathway may serve as an effective countermeasure against radiation-induced damage to the hematopoietic system.^55,56^ Together, these findings underscore the need for a more comprehensive characterization of strictly defined NO_2_FA derivatives on the NRF2 antioxidative response under physiological conditions.

## Conclusions

In this study, we investigated the effects of regio- and stereochemically defined NO_2_FA derivatives on NRF2 activation in primary bone marrow cells kept under physiologically relevant oxygen conditions. Our findings reveal that both **9-** and **10-NO**_**2**_ **OA**, as well as **10-NO**_**2**_ **LA**, effectively induce NRF2 target genes. Moreover, by addressing oxygen discrepancies typically overlooked in standard cell culture experiments, our approach provides a more accurate assessment of NRF2 activation. In summary, these results underscore the potential of highly pure NO_2_FA derivatives to modulate NRF2-mediated redox regulation and highlight the need for further studies to elucidate their roles in a physiologically relevant context.

## Supplementary Material





## Data Availability

The data that support the findings of this study related to chemical synthesis part are openly available in Zenodo at 10.5281/zenodo.14832958.

## Notes

Discussion about previous synthetic approaches to NO_2_FAs (Scheme S1, S2 and S3); detailed information on cytotoxicity (Figure S1 and Table S3) and the relative expression of *Hmox1* and *Gclm* under different oxygen conditions (Figure S2); key synthetic step optimization tables (Tables S1 and S2); experimental procedures and data characterization for all prepared compounds; copy of ^1^H NMR and ^13^C­{^1^H} NMR spectra for all prepared compounds; HPLC traces for lead compounds (PDF)
Molecular Formula Strings (CSV)

## Abbreviations Used

ARE: antioxidant response element
BM: bone marrow
BMC: bone-marrow cell
DAPI: 4′,6-diamidino-2-phenylindole
FBS: fetal bovine serum
GCLM: glutamate-cysteine ligase modifier subunit
HO-1/*Hmox1*: heme oxygenase-1
HRP: horse radish peroxidase
HSPC: hematopoietic stem and progenitor cell
IgG: immunoglobulin G
IL: interleukin
NO_2_FAs: nitro-fatty acids
NRF2/*Nfe2l2*: nuclear factor erythroid 2-related factor 2
Rpl32: ribosomal protein L32
SRB: sulforhodamine B
STAT: signal transducer and activator of transcription
TBST: tris-buffered saline-Tween
