# Supplementary material for: Regio- and Stereoselective Synthesis of Nitro-fatty Acids as NRF2 Pathway Activators Working under Ambient or Hypoxic Conditions
Source: J Med Chem. 2025 May 26;68(11):12172–84. doi: 10.1021/acs.jmedchem.5c00982 (PMC12169669; doi:10.1021/acs.jmedchem.5c00982)

Regio- and Stereoselective Synthesis of Nitro-fatty Acids as NRF2 Pathway Activators Working under Ambient or Hypoxic Conditions

Daniel Chrenko[a,#], Jana Pereckova[b,#], Martina Zatloukalova[c], Jan Vacek[c], Jiří Pospíšil[a,d,e,]*, Tomas Perecko[b,]*

Affiliation:

[a] Department of Chemical Biology, Faculty of Science, Palacky University, Šlechtitelů 27, CZ-783 71 Olomouc, Czech Republic.

[b] Department of Cell Biology and Radiobiology, Institute of Biophysics of the Czech Academy of Sciences, Kralovopolska 135, 612 00 Brno, Czech Republic

[c] Department of Medical Chemistry and Biochemistry, Faculty of Medicine and Dentistry, Palacky University, Hnevotinska 3, 775 15 Olomouc, Czech Republic

[d] Department of Organic Chemistry, Faculty of Science, Palacky University, tř. 17. listopadu 1192/12, CZ-771 46 Olomouc, Czech Republic

[e] Laboratory of Growth Regulators, Palacky University & Institute of Experimental Botany AS CR, Šlechtitelů 27, CZ-783 71 Olomouc, Czech Republic

Table of Contents

Supporting information 4
Nitro-fatty acids: synthesis and biological evaluation 4
Synthesis – Biomimetic approach 4
Synthesis – Henry reaction-based approach 5
Coupling step optimization (Table S1 and S2) 7
Cytotoxicity of tested NO_2FA derivatives (Figure S1) 9
Relative expression of (A) *Hmox1* and (B) *Gclm* in different oxygen conditions (Figure S2) 10
Calculated IC_{50} values of tested NO_2FA derivatives (Table S3) 11
Synthesis 12
General information 12
Nitro fatty acid synthesis 13
10-NO_2OA synthesis 13
9-NO_2OA synthesis 16
10-NO_2LA synthesis 19
9-NO_2cLA synthesis 26
10-NO_2SA synthesis 30
Literature 32
Copy of ^{1}H and ^{13}C{^{1}H} NMR spectra 34
Copy of ^{1}H and ^{13}C{^{1}H} spectra of **1a** 35
Copy of ^{1}H and ^{13}C{^{1}H} spectra of **3a** 36
Copy of ^{1}H and ^{13}C{^{1}H} spectra of (9*E*)-**10-NO_2MeOA** 37
Copy of ^{1}H and ^{13}C{^{1}H} spectra of **10-NO_2OA** 38
Copy of ^{1}H and ^{13}C{^{1}H} spectra of **S6** 39
Copy of ^{1}H and ^{13}C{^{1}H} spectra of **S7** 40
Copy of ^{1}H and ^{13}C{^{1}H} spectra of **1b** 41
Copy of ^{1}H and ^{13}C{^{1}H} spectra of **3b** 42
Copy of ^{1}H and ^{13}C{^{1}H} spectra of (9*E*)-**9-NO_2MeOA** 43
Copy of ^{1}H and ^{13}C{^{1}H} spectra of **9-NO_2OA** 44
Copy of ^{1}H and ^{13}C{^{1}H} spectra of **S9** 45
Copy of ^{1}H and ^{13}C{^{1}H} spectra of **S10** 46
Copy of ^{1}H and ^{13}C{^{1}H} spectra of **S11** 47
Copy of ^{1}H and ^{13}C{^{1}H} spectra of **4** 48

Copy of ^{1}H and $^{13}C\{^{1}H\}$ spectra of **S12** 49
Copy of ^{1}H and $^{13}C\{^{1}H\}$ spectra of **1c** 50
Copy of ^{1}H and $^{13}C\{^{1}H\}$ spectra of **2a** 51
Copy of ^{1}H and $^{13}C\{^{1}H\}$ spectra of **3c** 52
Copy of ^{1}H and $^{13}C\{^{1}H\}$ spectra of **10-NO$_2$MeLA** 53
Copy of ^{1}H and $^{13}C\{^{1}H\}$ spectra of **10-NO$_2$LA** 54
Copy of ^{1}H and $^{13}C\{^{1}H\}$ spectra of **5** 55
Copy of ^{1}H and $^{13}C\{^{1}H\}$ spectra of **S15** 56
Copy of ^{1}H and $^{13}C\{^{1}H\}$ spectra of **2c** 57
Copy of ^{1}H and $^{13}C\{^{1}H\}$ spectra of **9-NO$_2$MecLA** 58
Copy of ^{1}H and $^{13}C\{^{1}H\}$ spectra of **9-NO$_2$cLA** 59
Copy of ^{1}H and $^{13}C\{^{1}H\}$ spectra of **S16** 60
Copy of ^{1}H and $^{13}C\{^{1}H\}$ spectra of **10-nitrostearic acid (10-NO$_2$SA)** 61
HPLC traces for lead compounds 62
HPLC chromatogram of **10-NO$_2$LA** 62
HPLC chromatogram of **10-NO$_2$OA** 63
HPLC chromatogram of **9-NO$_2$OA** 64
HPLC chromatogram of **9-NO$_2$cLA** 65
HPLC chromatogram of **10-NO$_2$SA** 66

Supporting information

Nitro-fatty acids: synthesis and biological evaluation

Synthesis – Biomimetic approach

In general, two different synthetic approaches to nitro fatty acids are recognized: (a) biomimetic and (b) Henry reaction-based (for a recent overview of all synthetic sequences that yield NO_2FAs, see[1]). The first approach (biomimetic) is straightforward and similar to nature, is based on the transformation of fatty acids to the corresponding NO_2FAs. In *Scheme S 1*, the most commonly used approach is based on the nitro-anion addition/elimination protocol. In such cases, however, a mixture of regioisomers is formed (*Scheme S 1*).[2,3,3]

To demonstrate this approach, the reaction of oleic acid (OA) with $NaNO_2$ (source of the nitrite anion) in the presence of $HgCl_2$ and PhSeBr (electrophile) was performed. The depicted chromatogram clearly showed the presence of two NO_2OA regioisomers in a 42:58 ratio. The same can be observed from the ^{1}H NMR spectra (**9-NO₂OA** olefinic hydrogen, δ (ppm) = 7.0745 (t, J = 7.9 Hz, 1H); **10-NO₂OA** olefinic hydrogen, δ (ppm) = 7.0693 (t, J = 7.9 Hz, 1H)).

Similarly, if a radical-based biomimetic approach is used (*Scheme S 2*),[4] a roughly equimolar mixture of **9-NO₂OA** and **10-NO₂OA** is formed. However, in this case, two additional products, **S3** and **S4**, originating from the allylic radical addition, are formed, albeit in minor amounts. It should be pointed out that compounds **S3** and **S4** are presented in the form of two enantiomers and are formed as racemates.

It is important to pointed out that several even recent studies reported in the literature are using mixtures of various NO_2FAs, generally a mixture of 9- and 10-NO_2OA, in their biological studies (for recent examples see [4–9]).

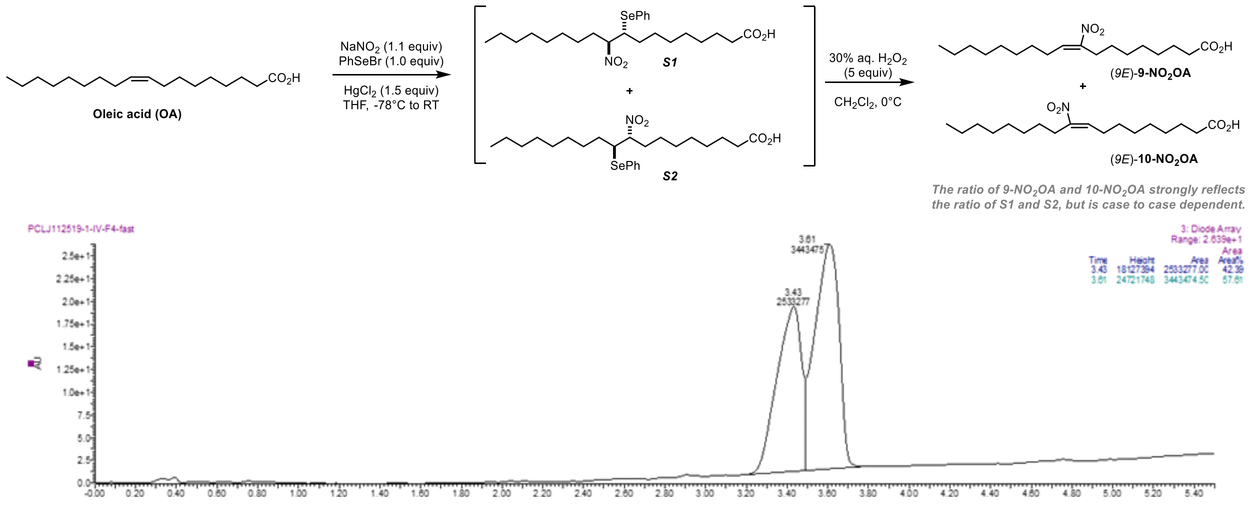

Scheme S 1. Biomimetic nitro-selenation/elimination protocol. *THF = tetrahydrofuran.*

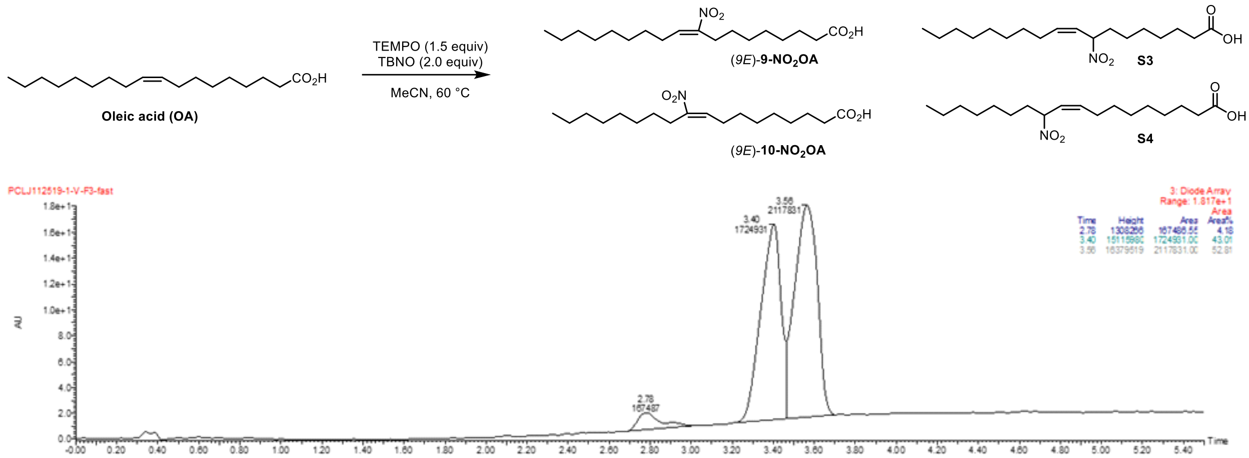

Scheme S 2. Nitroxylic radical-based approach to **9-NO₂OA** and **10-NO₂OA** accompanied by the formation of several side products. Compounds **S3** and **S4** side products are the most abundant. *TEMPO = (2,2,6,6-tetramethylpiperidin-1-yl)oxidanyl; TBNO = tert-Butyl nitrite; MeCN = acetonitrile.*

Synthesis – Henry reaction-based approach

The second approach is based on step-by-step synthesis, which usually involves Henry reaction (C-C making coupling step) followed by the elimination of a (generally) modified hydroxy group (*Scheme S 3*A). In general, such elimination furnishes the desired nitroolefin in various degrees of *E*/*Z* ratio. The key feature of this approach is the ability to control the regioselectivity (selection of the coupling partners for the Henry reaction; *Scheme S 3*A) as well as stereoselectivity (the elimination process is stereoselective and proceeds via *antiperiplanar* elimination; *Scheme S 3*B). This approach has been used in several cases in the synthesis of NO_2FAs (*Scheme S 3*C). First regio and stereospecific synthesis of 9 and 10-NO_2OA was reported in 2006 by Branchaud,[10] and the same approach was re-discovered in 2013 by Schopfer (*Scheme S 3*C1).[11] Both reported approaches yielded the desired NO_2OAs in good yields and with regio- and stereoselectivity. However, similar to another approach reported by Manolikakes in 2019[12] (*Scheme S 3*C2), they suffered from one crucial drawback: in the last step of the synthesis, Pd^0 catalysis is used to liberate the protected (allylic group) carboxylic acid of the targeted NO_2OA. Since in most cases, only one operation is used before the compounds are potentially used for biological evaluation (usually simple work-up), the presence of heavy metals in evaluated samples cannot be excluded.

It is important to point out that several studies reported in the literature have used one of the above-mentioned syntheses in SAR studies (for recent examples, see[13–17]). Only recently two new approaches that allow regio- and stereoselective synthesis of NO_2OA[18] and cLA[19] (*Scheme S 3*C3 and *Scheme S 3*C4, respectively) and that are heavy-metal free in the last two steps of synthesis appeared.

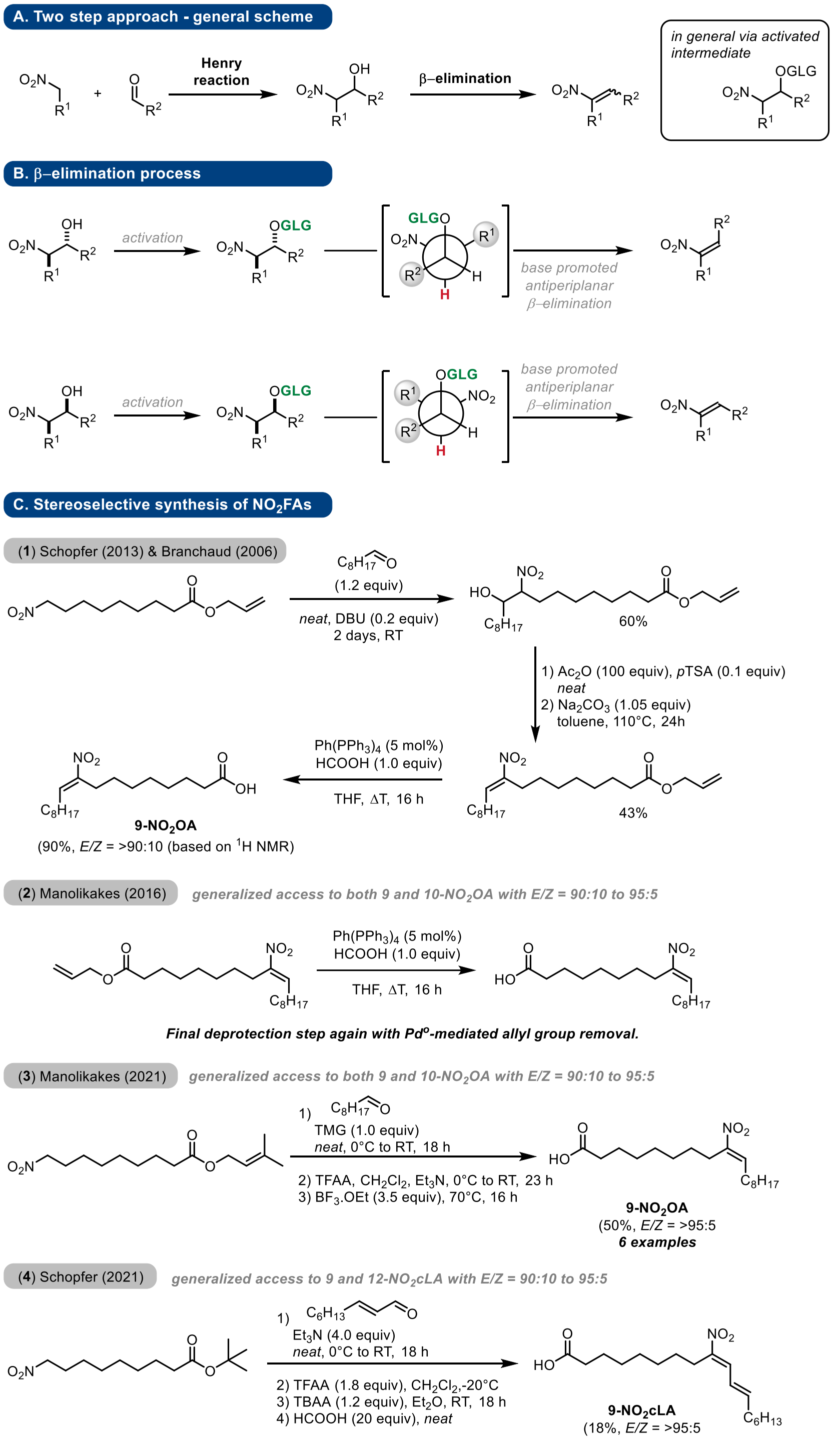

Scheme S 3. Non-biomimetic approach to nitroolefins based on the two-step protocol. **A.** Schematic view of the approach that consists of C-C bond connective Henry reaction (to fix the regioselectivity) and β-elimination (stereoselectivity) step to introduce the olefin. **B.** Antiperiplanar elimination process of activated β-hydroxy nitroalkane is responsible for the stereochemistry observed in final olefin. **C**. Stereoselective synthesis of NO_2FAs in literature. *cLA = conjugated linolic acid; DBU = 1,8-diazabicyclo[5.4.0]undec-7-ene; GLG = good leaving group; OA = Oleic acid; pTSA = 4-methylbenzene-1-sulfonic acid; TBAA = tetrabutylammonium acetate; TFAA = trifluoroacetic anhydride; THF = tetrahydrofuran; TMG = 1,1,3,3-tetramethylguanidine.*

Coupling step optimization (Table S1 and S2)

*Table S 1. Henry reaction optimization**

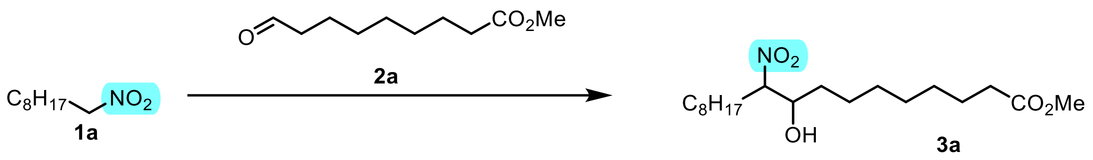

Entry	Conditions	2a [equiv]	Yield[a)] [%]	*d.r. ratio*[b)]
**1**	Et_3N (1.0 equiv), CH_2Cl_2 (0.1M), 0°C (1h) then RT (24h)	1.0	15	1.25:1
**2**	Et_3N (2.0 equiv), CH_2Cl_2 (0.1M), 0°C (1h) then RT (24h)	1.5	19	1.30:1
**3**	Et_3N (10.0 equiv), CH_2Cl_2 (0.1M), 0°C (1h) then RT (24h)	2.0	21	1.42:1
**4**	Et_3N (10.0 equiv), CH_2Cl_2 (0.1M), 0°C (1h) then RT (24h)	1.25	18	1.38:1
**5**	Et_3N (10.0 equiv), *neat*, 0°C (1h) then RT (24h)	1.2	23	1.55:1
**6**	DBU (1.0 equiv), *neat*, 0°C (1h) then RT (24h)	1.0	29	1.48:1
**7**	DBU (0.5 equiv), *neat*, 0°C (1h) then RT (12h)	1.25	34	1.50:1
**8**	TMG (1.0 equiv), *neat*, 0°C (1h) then RT (15h)	1.1	45	1.45:1
**9**	TMG (1.0 equiv), *neat*, RT (16h)	1.1	46	1.46:1
**10**	TMG (0.5 equiv), *neat*, RT (12h)	1.05	51	1.47:1
**11**	TMG (0.2 equiv), *neat*, RT (12h)	1.05	67	1.48:1
**12**	TMG (0.1 equiv), *neat*, RT (24h)	1.05	66	1.45:1

[a)] Refers to the pure isolated compound. [b)] The ratio of diastereoisomers was determined by ^1H NMR spectroscopy and refers to the ratio in isolated compound **3a**.
DBU = 1,8-diazabicyclo[5.4.0]undec-7-ene; TMG = 1,1,3,3-tetramethylguanidine; RT = room temperature (23 °C).

* The reaction was carried out on a 2 mmol scale of **1a**. Yields are reported as the average yield of three independent experiments. The lowest detected *E/Z* ratio within these three experiments is reported.

Table S 2. β-elimination step optimization.†

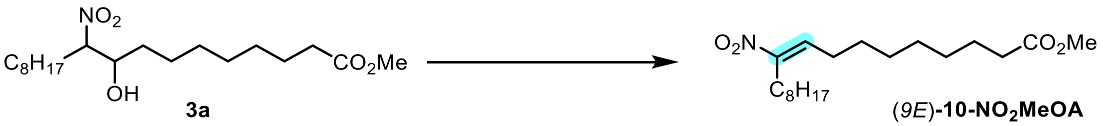

Entry	Conditions	Yield[a)] [%]	*E/Z ratio*[b)]
**1**	Ac_2O (1.0 equiv), AcONa (1.5 equiv), THF, 0°C (5h)	11	2:1
**2**	Ac_2O (1.0 equiv), AcONa (1.5 equiv), CH_2Cl_2, 0°C (8h)	13	3:1
**3**	Ac_2O (2.0 equiv), AcONa (2.1 equiv), CH_2Cl_2, 0°C (1h) then RT (12h)	17	4:1
**4**	Ac_2O (2.0 equiv), Et_3N (2.1 equiv), CH_2Cl_2, 0°C (1h) then RT (12h)	15	7:1
**5**	Ac_2O (2.0 equiv), Et_3N (3.0 equiv), CH_2Cl_2, 0°C (1h) then RT (12h)	19	6:1
**6**	Ac_2O (1.5 equiv), Et_3N (3.0 equiv), CH_2Cl_2, 0°C (1h) then RT (12h)	20	7:1
**7**	Ac_2O (1.5 equiv), *i*Pr_2NEt (3.0 equiv), CH_2Cl_2, 0°C (1h) then RT (12h)	12	10:1
**8**	TFAA (1.5 equiv), *i*Pr_2NEt (3.0 equiv), CH_2Cl_2, 0°C (1h) then RT (12h)	45	15:1
**9**	TFAA (1.5 equiv), Et_3N (3.0 equiv), CH_2Cl_2, 0°C (1h) then RT (12h)	69	30:1
**10**	TFAA (1.5 equiv), Et_3N (3.0 equiv), CH_2Cl_2, 0°C (4h) then RT (24h)	88	40:1
**11**	TFAA (1.5 equiv), Et_3N (3.0 equiv), DCE, 0°C (4h) then RT (24h)	85	99:1 (99:1)
**12**	TFAA (1.5 equiv), Et_3N (3.0 equiv), DCE, 0°C (1h) then RT (24h)	75	96:1 (98:1)

[a)] Refers to the pure isolated compound. [b)] Based on ^1H NMR spectra analysis of the crude reaction mixture. [c)] The ratio in brackets refers to the E/Z ratio of isolated and purified (9*E*)-**10-NO₂MeOA**, based on ^1H NMR spectra.
DBU = 1,8-diazabicyclo[5.4.0]undec-7-ene; DCE = 1,2-dichloroethan; TMG = 1,1,3,3-tetramethylguanidine; RT = room temperature (23 °C).

† The reaction was carried out on a 2 mmol scale of **3a**. Yields are reported as the average yield of three independent experiments. The lowest detected *E/Z* ratio within these three experiments is reported.

Cytotoxicity of tested NO_2FA derivatives (Figure S1)

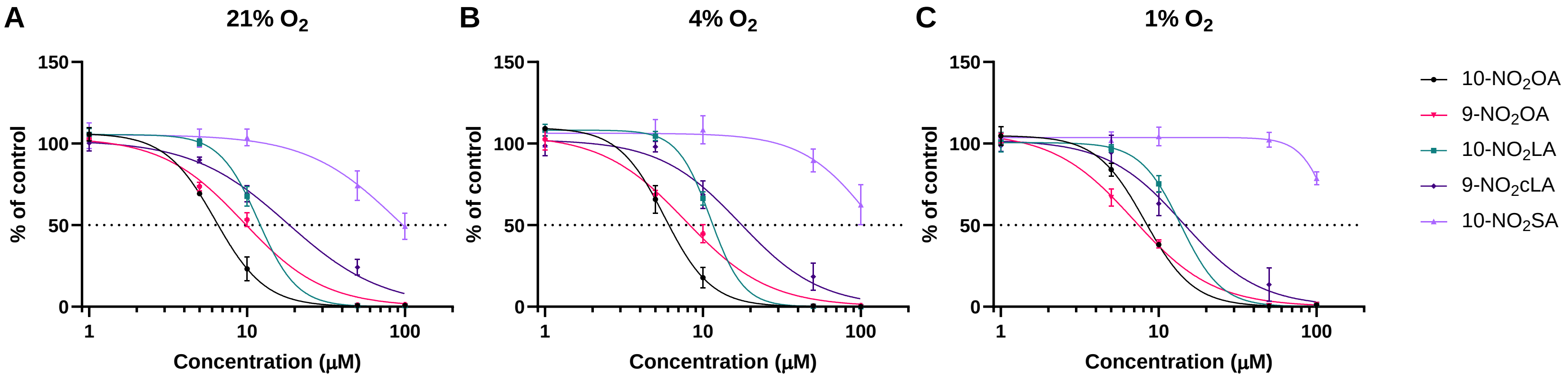

Figure S 1. ***Cytotoxicity of tested NO_2FA derivatives.*** *Mouse embryonic fibroblasts (MEFs) were cultured under varying oxygen conditions (21, 4, and 1%) and treated for 24 hours with the tested NO_2FAs, after which adherent cells were stained with sulforhodamine B. Results are presented as a percentage of control cells across different oxygen culture conditions (A–C). IC_{50} values were determined from dose-response curves (see Supplementary Table S3). Data are expressed as mean ± SD from three independent experiments (n = 3).*

Relative expression of (A) *Hmox1* and (B) *Gclm* in different oxygen conditions (Figure S2)

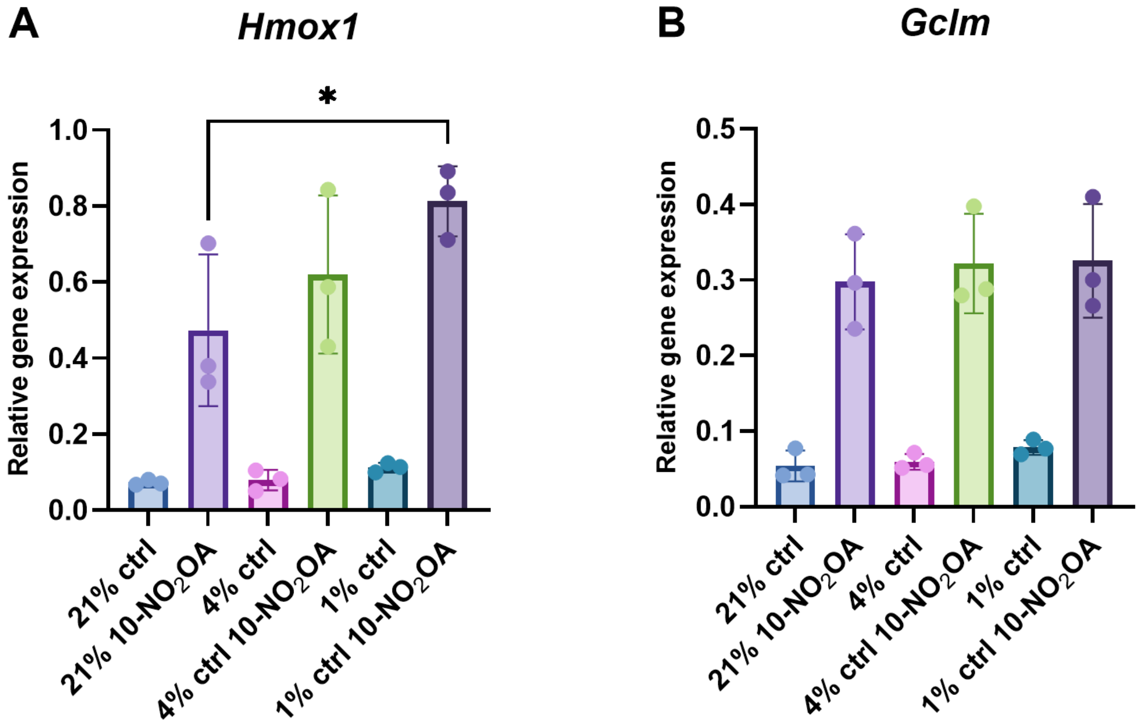

Figure S 2. ***Relative expression of (A) Hmox1 and (B) Gclm in different oxygen conditions.*** *BMCs were cultured under varying oxygen conditions (21, 4, and 1%) and treated for 4 hours with the tested 10-NO_2OA (2 µM) or medium followed by quantification with PCR. Results are presented as relative expression across different oxygen culture conditions. Beside upregulation of Hmox1 (A) and Gclm (B) expression compared to respective controls (significances depicted in the main text), there was significant upregulation comparing the effects of 10-NO_2OA in 21 and 1% oxygen, respectively in Hmox1 gene. This was not observed in Gclm. Data are expressed as mean ± SD from three independent experiments (n = 3), analyzed with one-way ANOVA with Bonferroni's multiple comparisons test.*

Calculated IC_{50} values of tested NO_2FA derivatives (Table S3)

Table S 3. Calculated IC_{50} values of tested NO_2FA derivatives.

IC_{50}	21% O_2		4% O_2		1% O_2	
	**Value**	**SEM**	**Value**	**SEM**	**Value**	**SEM**
10-NO_2OA	6.28	0.19	5.74	0.20	8.19	0.21
9-NO_2OA	9.47	0.57	7.75	0.54	6.93	0.32
10-NO_2LA	11.78	0.41	11.17	0.26	13.79	1.02
9-NO_2cLA	18.03	1.96	17.19	2.26	14.31	1.82
10-NO_2SA	≈100		>100		>100	

IC_{50} values were derived from dose-response curves using four-parameter logistic regression across different oxygen culture conditions. Data are presented as mean and SEM values from three independent experiments (n = 3).

The IC_{50} values calculated for cultures in 21% oxygen indicate an increasing cytotoxic potential in the order: 10-NO_2OA > 9-NO_2OA > 10-NO_2LA > 9-NO_2cLA. The same order of cytotoxic potential was observed in 4% oxygen, with nearly identical IC_{50} values. In 1% oxygen, the order of cytotoxic potential shifted slightly to 9-NO_2OA > 10-NO_2OA > 10-NO_2LA > 9-NO_2cLA. However, differences in IC_{50} values across the oxygen conditions for each NO_2FA derivative ranged from ca. 1 to 3 µM. In contrast, the IC_{50} values of the saturated derivative 10-NO_2SA were ≥100 µM under all three oxygen conditions.

Synthesis

General information

All starting materials were purchased from commercial suppliers and used without further purification, unless otherwise stated. Anhydrous Tetrahydrofuran (THF), diethylether (Et_2O), and dichloromethane (DCM) were purchased from Sigma-Aldrich. All reactions were performed in round-bottom flasks fitted with rubber septa using standard laboratory techniques under positive pressure of argon (Air Liquide, >99.5% purity). Anhydrous solvents were purchased from Sigma-Aldrich. Purification of reaction products was carried out by column chromatography using standard grade silica gel (60 Å, 230–400 mesh), or by semiprep HPLC Agilent 1290 Infinity II with UV-VIS with a mass detector Agilent InfinityLab LC/MSD using the C18 reverse-phase column (Agilent 5Prep-C18 10x21.2 mm). The gradient was formed from water and methanol with a flow rate of 15 mL/mid or 20 mL/min. Analytical thin-layer chromatography was performed on a thin-layer chromatography (TLC) aluminum plates pre-coated with silica gel (silica gel 60 F_{254}). Visualization was accomplished with UV light, phosphomolybdic acid, and potassium permanganate stains, followed by heating. The ^{1}H NMR and $^{13}C\{^{1}H\}$ NMR spectra were measured on Jeol ECA400II (400 and 101 MHz) or Jeol 500 ECA (500 and 126 MHz) in Chloroform-*d*. Chemical shifts are reported in ppm, and their calibration was performed (a) in the case of ^{1}H NMR experiments on the residual peak of non-deuterated solvent δ ($CHCl_3$) = 7.26 ppm; in the case of ^{13}C NMR experiments on the middle peak of the ^{13}C signal in deuterated solvent δ ($CDCl_3$) = 77.16 ppm. The proton coupling patterns are represented as a singlet (s), a doublet (d), a doublet of a doublet (dd), a triplet (t), a triplet of a triplet (tt), and a multiplet (m). High-resolution mass spectrometry (HRMS) on Agilent 6230 high-resolution mass spectrometer with electrospray ionization (ESI) and a time-of-flight analyzer operating in a positive or negative full scan mode in the range of 100 − 1700 m/z. High-performance liquid chromatography (HPLC) was performed using an Agilent 1290 Infinity II system with UV-VIS detector and an Agilent InfinityLab LC/MSD mass detector.

Lipase B *Candida antarctica* (CAL-B Lipase) immobilized from *Candida antarctica*, beads, white, >2 units/g, EC number 232-619-9, batch BCCH3575, was purchased from Sigma-Aldrich. Aqueous phosphate buffer (pH = 7.4) was prepared using "Quest Calculate™ PBS (Phosphate Buffered Saline) (1X, pH 7.4) Preparation and Recipe" protocol (https://www.aatbio.com/resources/buffer-preparations-and-recipes/pbs-phosphate-buffered-saline)

Nitro fatty acid synthesis

10-NO_2OA synthesis

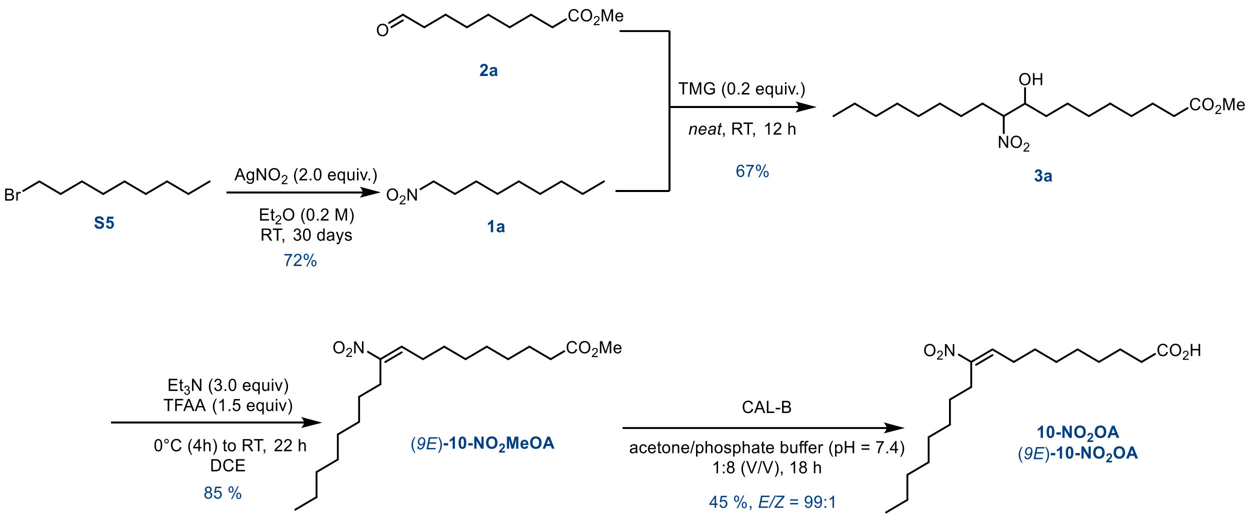

*1-nitrononane (**1a**)*

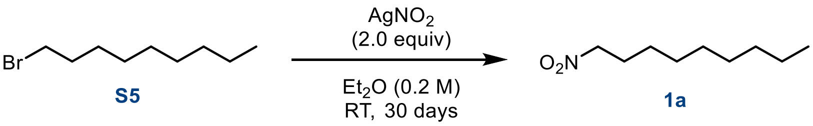

Reaction flasks must be covered in aluminum foil to protect the reaction mixture against the sunlight.

To a solution of bromide **S5** (4 g, 19.3 mmol, 1.0 equiv.) in Et_2O (0.2 M, 96 mL), $AgNO_2$ (6 g, 38.6 mmol, 2.0 equiv.) was added. The flask was closed with a stopper and covered with aluminum foil to avoid the sunlight. The resulting suspension was stirred for 30 days at room temperature (RT). The reaction mixture was diluted with EtOAc (50 mL) and the resulting mixture was filtered through a short plug of Celite®. The filter pad was rinsed with EtOAc (2 × 100 mL), and the combined filtrates were concentrated under reduced pressure. The crude product was purified by flash column chromatography (SiO_2; petroleum ether:EtOAc = 1000:1) and yielded product **1a** (2.43 g, 72%) as a colourless oil.

^{1}H NMR (500 MHz, Chloroform-*d*) *δ* (ppm): 4.37 (t, J = 7.1 Hz, 2H), 2.03 – 1.96 (m, 2H), 1.41 – 1.19 (m, 12 h), 0.87 (t, J = 6.9 Hz, 3H).

^{13}C {^{1}H} NMR (126 MHz, Chloroform-*d*) *δ* (ppm): 75.9, 31.9, 29.4, 29.3, 29.0, 27.6, 26.4, 22.8, 14.2.

*Methyl 9-hydroxy-10-nitrooctadecanoate (**3a**)*

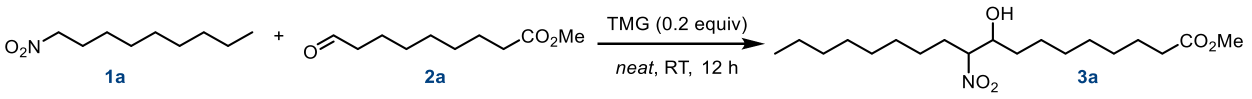

A neat mixture of 1-nitrononane **1a** (0.17 g, 1 mmol, 1.0 equiv.) and aldehyde **2a** (0.23 g, 1.2 mmol, 1.2 equiv.) was cooled to 0°C and 1,1,3,3-tetramethylguanidine (0.02 g, 0.2 mmol, 0.2 equiv.) was added. The resulting mixture was allowed to stir at room temperature for 12 h, before it was cooled to 0 °C, and the reaction was terminated by H_2O (5 mL) addition. The resulting mixture was extracted with EtOAc

(3×15 mL) and the combined organic layers were dried over Na_2SO_4, filtered and the solvents were removed under reduced pressure. The crude product was purified by flash column chromatography (SiO_2; petroleum ether:EtOAc = 50:1) and yielded the adduct **3a** (0.24 g, 67 %; *d.r.* = 1.48:1) as a pale-yellow oil. Product **3a** was obtained as a mixture of two diastereoisomers in a 1.48:1 *d.r.* ratio (based on the ^{1}H NMR spectra analysis).

^{1}H NMR (500 MHz, Chloroform-*d*) δ (ppm): 4.43 (ttd, *J* = 9.3, 4.0, 1.7 Hz, 1H), 4.00 (ddq, *J* = 8.7, 4.7, 2.0 Hz, 0.5H), 3.85 (pd, *J* = 7.3, 6.4, 2.8 Hz, 0.5H), 3.66 (s, 3H), 2.34 (dd, *J* = 4.9, 3.0 Hz, 0.5H), 2.30 (t, *J* = 7.5 Hz, 2H), 2.18 (dd, *J* = 8.0, 3.4 Hz, 0.5H), 2.14 – 1.95 (m, 1H), 1.83 – 1.72 (m, 1H), 1.63 – 1.59 (m, 2H), 1.53 – 1.40 (m, 3H), 1.33 – 1.23 (m, 18H), 0.87 (t, *J* = 7.0 Hz, 3H).

^{13}C {^{1}H} NMR (126 MHz, Chloroform-*d*) δ (ppm): 174.5, 93.1, 92.5, 72.4, 72.1, 51.6, 34.1, 33.6, 33.2, 31.9, 30.5, 29.29, 29.20, 29.18, 29.11, 29.0, 28.1, 26.1, 25.8, 25.6, 25.2, 24.9, 22.7, 14.2.

MS (ESI) *m/z* (%): 361 $[M+H]^+$.

HRMS (ESI) *m/z:* $[M+H]^+$ calculated for $C_{19}H_{38}NO_5$: 360.2744; found: 360.2748.

R_f = 0.19 (PMA, *n*-Hexane:EtOAc = 10:1)

*Methyl (9E)-10-nitrooctadec-9-enoate (**(9E)-10-NO₂MeOA**)*

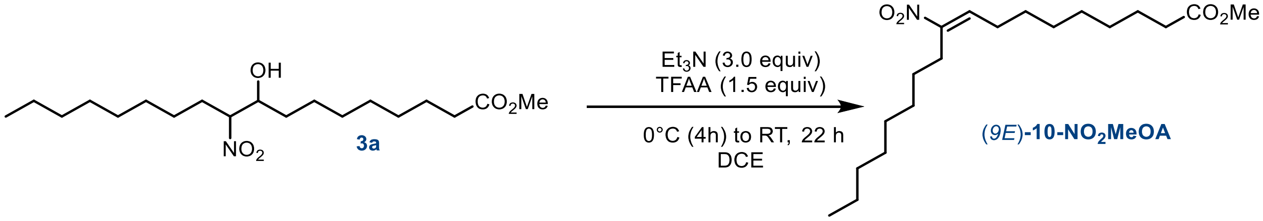

A mixture of diastereoisomers **3a** (0.24 g, 0.66 mmol, 1.0 equiv.) was dissolved in DCE (3.3 mL, 0.2M) and the resulting mixture was cooled to 0 °C. Et_3N (0.28 mL, 1.98 mmol, 3.0 equiv.) followed by trifluoroacetic anhydride (TFAA) (0.14 mL, 0.99 mmol, 1.5 equiv.) were sequentially added. The whole mixture was then stirred at 0 °C for 4 h and at room temperature for next 22 h.

The whole mixture was cooled to 0 °C and H_2O (10 mL) was added to terminate the reaction. The resulting mixture was extracted with EtOAc (3×15 mL), and organic phases were combined, dried over $MgSO_4$, and the volatiles were removed *in vacuo*. The crude product was purified by semipreparative HPLC chromatography (C18 reverse-phase column; MeOH:H_2O) and yielded the desired nitro olefin (9*E*)-**10-NO₂MeOA** (0.194g, 85 %; *E/Z* = >99:1 based on the ^{1}H NMR spectra analysis) in the form of a colorless oil.

^{1}H NMR (500 MHz, Chloroform-*d*) δ (ppm): 7.07 (t, *J* = 7.9 Hz, 1H), 3.67 (d, *J* = 1.0 Hz, 3H), 2.60 – 2.53 (m, 2H), 2.31 (t, *J* = 7.5 Hz, 2H), 2.21 (q, *J* = 7.6 Hz, 2H), 1.62 (p, *J* = 7.2 Hz, 2H), 1.48 (h, *J* = 8.8, 8.2 Hz, 4H), 1.38 – 1.23 (m, 16H), 0.88 (t, *J* = 7.0 Hz, 3H).

^{13}C {^{1}H} NMR (126 MHz, Chloroform-*d*) δ (ppm): 174.4, 152.1, 136.4, 51.6, 34.1, 32.0, 29.4, 29.32, 29.29, 29.14, 29.11, 28.6, 28.12, 28.05, 26.5, 25.0, 22.8, 14.2.

MS (ESI) *m/z* (%): 343 $[M+H]^+$.

HRMS (ESI) ***m/z:*** $[M+H]^+$ calculated for $C_{19}H_{36}NO_4$: 342.2639; found: 342.2642.

*(E)-9-nitrooctadec-9-enoic acid (**10-NO₂OA**)*

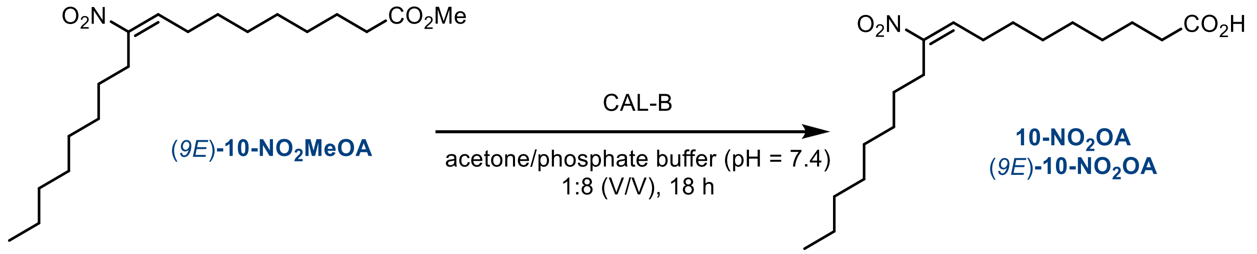

CAL-B (400 mg) was added to a solution of methyl ester (9*E*)-**10-NO₂MeOA** (0.3 g, 0.88 mmol, 1.0 equiv.) in acetone (11 mL, 0.08 M) and aqueous phosphate buffer (88 mL, 0.01 M, pH 7.4). The resulting solution was vigorously stirred (magnetic stirrer, 1000 rpm) at room temperature for 18 h, before the pH of the solution was adjusted to pH = 3 by 1.0 M aq. HCl. The whole mixture was then extracted with EtOAc (5x20 mL), and the combined organic layers were dried over $MgSO_4$, filtered, and the volatiles were removed *in vacuo*. The crude product was purified by semipreparative HPLC chromatography (C18 reverse-phase column; $MeOH:H_2O$) and yielded the desired acid **10-NO₂OA** (0.13 g; 45 %, *E/Z* = >99:1 based on the ^1H NMR spectra analysis).

^{1}H NMR (500 MHz, Chloroform-*d*) *δ* (ppm): 10.50 (broad s, 1H), 7.07 (t, J = 7.9 Hz, 1H), 2.59 – 2.54 (m, 2H), 2.36 (t, J = 7.5 Hz, 2H), 2.21 (q, J = 7.6 Hz, 2H), 1.67 – 1.60 (m, 2H), 1.53 – 1.44 (m, 4H), 1.39 – 1.32 (m, 6H), 1.32 – 1.24 (m, 10H), 0.88 (t, J = 6.8 Hz, 3H).

^{13}C {^{1}H} NMR (126 MHz, Chloroform-*d*) *δ* (ppm): 152.1, 136.4, 33.8, 32.0, 29.4, 29.38, 29.32, 29.1, 29.0, 28.6, 28.12, 28.05, 26.5, 24.7, 22.8, 14.2.

MS (ESI) ***m/z*** **(%):** 327 $[M-H]^-$.

HRMS (ESI) ***m/z:*** $[M-H]^-$ calculated for $C_{18}H_{32}NO_4$: 326.2337; found: 326.2340.

9-NO$_2$OA synthesis

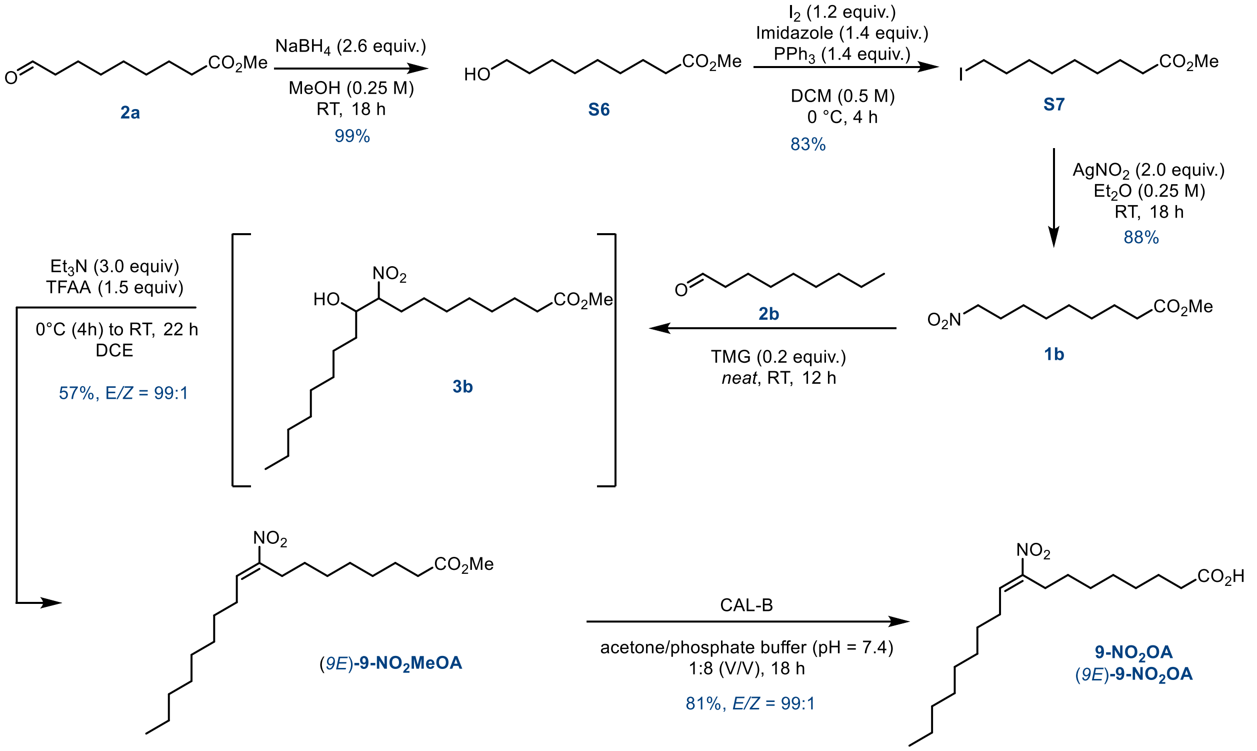

*Methyl 9-hydroxynonanoate (**S6**)*

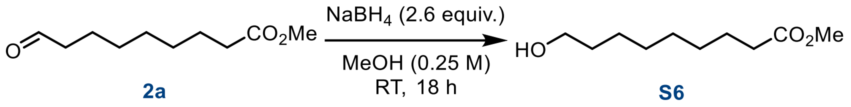

$NaBH_4$ (1.7 g, 44.2 mmol, 2.6 equiv.) was added portion wise to a cold (0 °C, water/ice) solution of aldehyde **2a** (3.2 g, 17 mmol, 1.0 equiv.) in MeOH (68 mL, 0.25 mL) and the resulting mixture was stirred at RT for 18h. Water (50 mL), and EtOAc (100 mL) were added and the resulting phases were separated. The aqueous layer was extracted with EtOAc (3x50 mL), and the combined organic layers were dried over $MgSO_4$, filtered, and the volatiles were removed under reduced pressure. The crude product was purified by flash column chromatography (SiO_2; petroleum ether:EtOAc = 10:1) and yielded the desired alcohol **S6** (3.17 g, 99 %) as a colorless oil.

^{1}H NMR (500 MHz, Chloroform-*d*) *δ* (ppm): 3.66 (d, *J* = 2.0 Hz, 3H), 3.63 (t, *J* = 6.6 Hz, 2H), 2.30 (td, *J* = 7.4, 2.0 Hz, 2H), 1.64 – 1.59 (m, 2H), 1.59 – 1.52 (m, 2H), 1.37 – 1.26 (m, 8H).

^{13}C {^{1}H} NMR (126 MHz, Chloroform-*d*) *δ* (ppm): 174.5, 63.2, 51.6, 34.2, 32.9, 29.32, 29.28, 29.17, 25.8, 25.0.

*Methyl 9-iodononanoate (**S7**)*

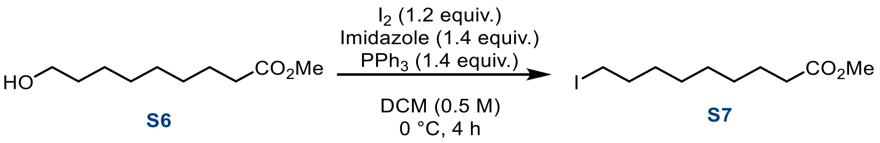

Reaction flasks must be covered in aluminum foil to protect the reaction mixture against the sunlight.

To a cold (0°C, ice/water) solution of alcohol **S6** (3.1 g, 16.5 mmol) in CH_2Cl_2 (0.5 M, 33 mL), imidazole (1.57 g, 22.8 mmol, 1.4 equiv.), PPh_3 (6.05 g, 22.8 mmol, 1.4 equiv.), and I_2 (5 g, 19.6 mmol, 1.2 equiv.) were sequentially added (the reaction flask was covered by aluminum foil to avoid the direct sun light). The resulting mixture was stirred at 0°C for 3.5 h before it was diluted with EtOAc (100 mL). The resulting mixture was filtered through a pad of silica gel and the filtrate was concentrated under reduced pressure. The crude product was purified by flash column chromatography (SiO_2; petroleum ether:EtOAc = 10:1) and yielded the desired iodide **S7** (4.08 g, 83 %) as a pale orange oil.

^{1}H NMR (500 MHz, Chloroform-*d*) *δ* (ppm): 3.67 (s, 3H), 3.18 (t, J = 7.0 Hz, 2H), 2.30 (t, J = 7.5 Hz, 2H), 1.81 (p, J = 7.1 Hz, 2H), 1.64 – 1.59 (m, 2H), 1.41 – 1.35 (m, 2H), 1.34 – 1.23 (m, 6H).

^{13}C {^{1}H} NMR (126 MHz, Chloroform-*d*) *δ* (ppm): 174.4, 51.6, 34.2, 33.6, 30.5, 29.17, 29.15, 28.5, 25.0, 7.4.

*Methyl 9-nitrononanoate (**1b**)*

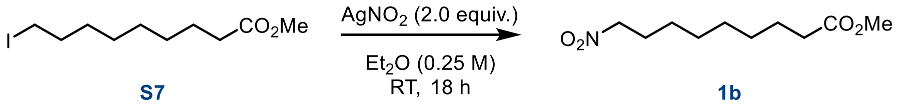

Reaction flasks must be covered in aluminum foil to protect the reaction mixture against the sunlight.

To a solution of iodide **S7** (4.0 g, 13.3 mmol, 1.0 equiv.) in Et_2O (0.2 M, 66 mL), $AgNO_2$ (4.1 g, 26.6 mmol, 2.0 equiv.) was added. The reaction flask was closed with a stopper and the resulting suspension was stirred overnight at RT. The mixture was diluted with EtOAc (100 mL) and filtered through a short plug of Celite®. The filter cake was washed with EtOAc (2x100 mL), and the combined filtrates were concentrated under reduced pressure. The crude product was purified by flash column chromatography (SiO_2; petroleum ether:EtOAc = 4:1) and yielded the desired nitro alkane **1b** (2.54 g, 88 %) in the form of colorless oil.

^{1}H NMR (500 MHz, Chloroform-*d*) *δ* (ppm): 4.37 (t, *J* = 7.0 Hz, 2H), 3.66 (s, 3H), 2.30 (t, *J* = 7.5 Hz, 2H), 2.00 (p, *J* = 7.1 Hz, 2H), 1.62 (q, *J* = 7.4 Hz, 2H), 1.41 – 1.26 (m, 8H).

^{13}C {^{1}H} NMR (126 MHz, Chloroform-*d*) *δ* (ppm): 174.4, 75.8, 51.6, 51.6, 34.1, 29.03, 29.01, 28.8, 27.5, 26.3, 24.9.

*Methyl (E)-9-nitrooctadec-9-enoate ((9E)-**9-NO$_2$MeOA**)*

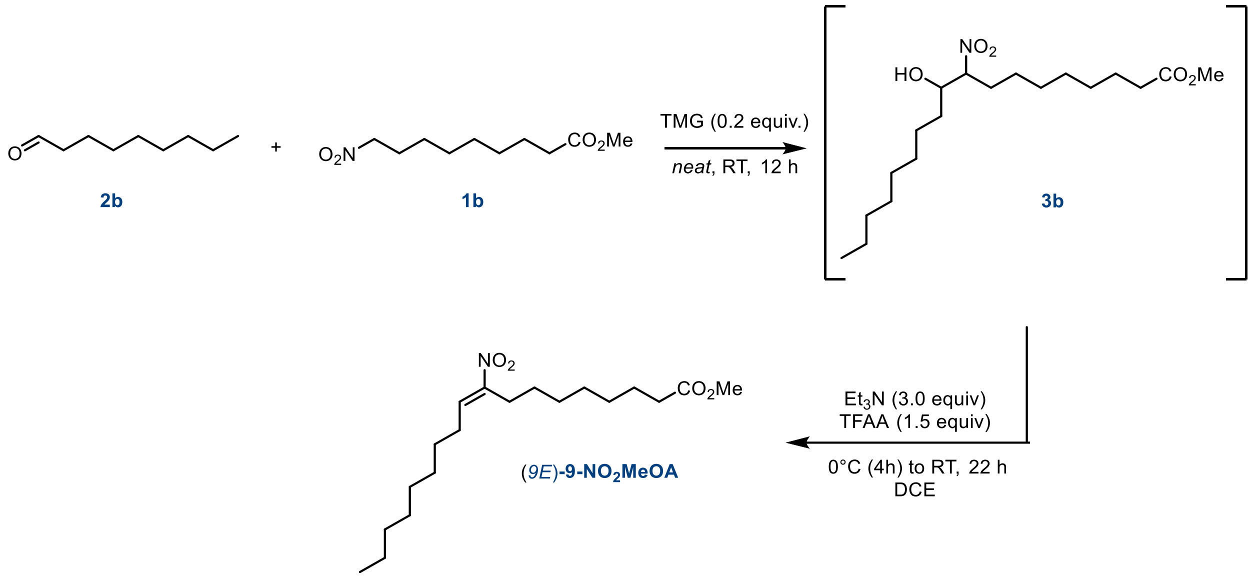

A mixture of nitroalkane **1b** (1.2 g, 5.47 mmol, 1.0 equiv.) and nonanal **1b** (0.96 g, 6.56 mmol, 1.2 equiv.) was cooled to 0 °C and 1,1,3,3-tetramethylguanidine (TMG) (0.14 g, 1.1 mmol, 0.2 equiv.) was added. The resulting mixture was stirred at RT for 12 h, after which time the reaction mixture was cooled to 0 °C and terminated with H_2O (25 mL). The resulting mixture was extracted with EtOAc (3×30 mL), and the combined organic phases were dried over $MgSO_4$. The solvent was removed *in vacuo* and the crude product **3b** was used directly without further purification in the next step.

Crude adduct **3b** was dissolved in DCE (27 mL, 0.2 M) and cooled to 0 °C. Et_3N (2.28 mL, 16.4 mmol, 3.0 equiv.) followed by TFAA (1.14 mL, 8.2 mmol, 1.5 equiv.) were sequentially added and the resulting mixture was stirred at 0 °C for 4 h and then additional 22h at RT. The whole mixture was cooled to 0 °C (ice/water) and H_2O (75 mL) was added. The resulting mixture was extracted with EtOAc (3×100 mL), and collected organic phases were combined, dried over $MgSO_4$, and the volatiles were removed *in vacuo*. The crude product was purified by the semipreparative HPLC chromatography (C18 reverse-phase column; MeOH:H_2O) to yield the desired methyl 9-nitro oleate (9*E*)-**9-NO$_2$MeOA**) (1.06 g, 57 %, *E/Z* = >99:1 based on the ^1H NMR spectra analysis).

^1H NMR (500 MHz, Chloroform-*d*) *δ* (ppm): 7.08 (t, *J* = 7.9 Hz, 1H), 3.66 (s, 3H), 2.60 – 2.53 (m, 2H), 2.30 (t, *J* = 7.5 Hz, 2H), 2.21 (q, *J* = 7.6 Hz, 2H), 1.61 (p, *J* = 7.2 Hz, 2H), 1.48 (h, *J* = 7.5 Hz, 4H), 1.37 – 1.22 (m, 16H), 0.90 – 0.83 (m, 3H).

^{13}C {^1H} NMR (126 MHz, Chloroform-*d*) *δ* (ppm): 174.4, 151.9, 136.7, 51.6, 34.2, 31.9, 29.5, 29.4, 29.3, 29.2, 29.12, 29.05, 28.7, 28.2, 28.0, 26.5, 25.0, 22.8, 14.2.

MS (ESI) *m/z* (%): 343 $[M+H]^+$.

HRMS (ESI) *m/z:* $[M+H]^+$ calculated for $C_{19}H_{36}NO_4$: 342.2639; found: 342.2638.

*(E)-9-nitrooctadec-9-enoic acid (**9-NO₂OA**)*

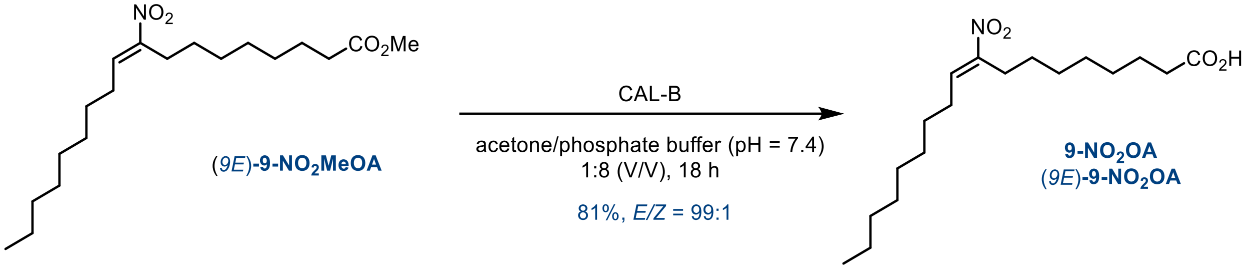

CAL-B (400 mg) was added to a solution of methyl ester (9*E*)-**9-NO$_2$MeOA** (0.13 g, 0.38 mmol, 1.0 equiv.) in acetone (4.7 mL, 0.08 M) and aqueous phosphate buffer (38 mL, 0.01 M, pH 7.4). The solution was vigorously stirred (magnetic stirrer, 1000 rpm) at room temperature for 18 h. The whole mixture was adjusted by 1 M aq. HCl to pH = 3. The whole mixture was then extracted with EtOAc (5x20 mL), and the combined organic layers were dried over $MgSO_4$, filtered, and organic solvents were removed under reduced pressure. The crude product was purified by semipreparative HPLC chromatography (C18 reverse-phase column; MeOH:H_2O) to yield the desired acid **9-NO$_2$OA** (0.1 g, 81 %, *E/Z* ≥ 99:1 based on the ^{1}H NMR spectra analysis).

^{1}H NMR (500 MHz, Chloroform-*d*) *δ* (ppm): 11.53 (broad s, 1H), 7.07 (t, *J* = 7.9 Hz, 1H), 2.60 – 2.51 (m, 2H), 2.34 (t, *J* = 7.5 Hz, 2H), 2.20 (q, *J* = 7.6 Hz, 2H), 1.62 (p, *J* = 7.4 Hz, 2H), 1.48 (h, *J* = 7.2 Hz, 4H), 1.37 – 1.29 (m, 6H), 1.32 – 1.21 (m, 10H), 0.92 – 0.83 (m, 3H).

^{13}C {^{1}H} NMR (126 MHz, Chloroform-*d*) *δ* (ppm):152.1, 136.4, 33.9, 32.0, 29.4, 29.32, 29.27, 29.1, 29.0, 28.6, 28.11, 28.05, 26.5, 24.7, 22.8, 14.2.

HRMS (ESI) *m/z:* [M-H]$^-$ calculated for $C_{18}H_{32}NO_4$: 326.2337; found: 326.2326.

10-NO$_2$LA synthesis

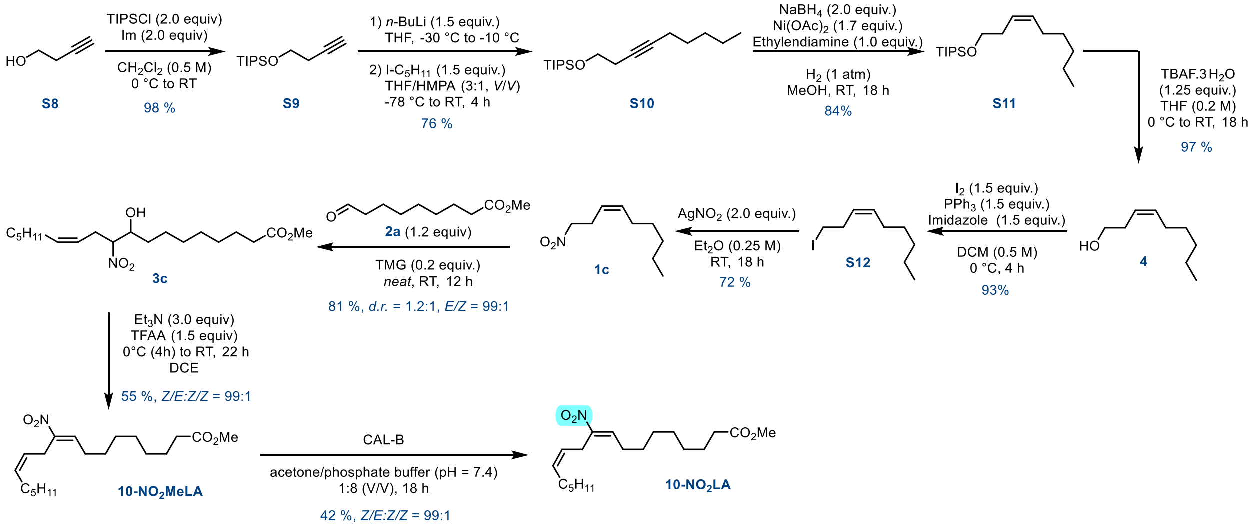

*(But-3-yn-1-yloxy)triisopropylsilane (**S9**)*

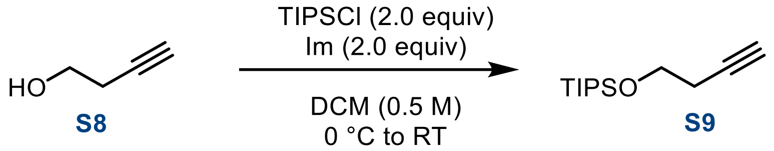

A solution of imidazole (18.2 g, 263 mmol, 2.0 equiv.) in DCM (250 mL, 0.5 M) was cooled to 0 °C and but-3-yn-1-ol **S8** (10 mL, 131.0 mmol, 1.0 equiv.) was added. After 5 min, TIPSCl (59 mL, 263 mmol, 2.0 equiv.) was added within a period of 5 min, and the whole mixture was stirred at room temperature for 16h. H_2O (50 mL) was added, and the resulting layers were separated. The aqueous layer was extracted with CH_2Cl_2 (3x150 mL) and resulting organic layers were combined, washed with brine (100 mL), dried over Na_2SO_4, filtered, and solvents were removed under reduced pressure. The crude product was purified by flash column chromatography (SiO_2; petroleum ether:EtOAc = 100:1) and yielded the desired silyl ether **S9** (29.1 g, 98 %) in the form of a colorless oil.

^{1}H NMR (500 MHz, Chloroform-*d*) δ (ppm): 3.82 (t, *J* = 7.3 Hz, 2H), 2.44 (td, *J* = 7.3, 2.7 Hz, 2H), 1.96 (t, *J* = 2.7 Hz, 1H), 1.10 – 1.03 (m, 21H).

^{13}C {^{1}H} NMR (126 MHz, Chloroform-*d*) δ (ppm): 81.7, 69.4, 62.1, 23.0, 18.1, 12.1.

MS (ESI) *m/z* (%): 227 $[M+H]^+$.

R*f* = 0.45 (vanillin, green spot; petroleum ether:EtOAc = 10:1)

Data matched to those previously reported.[2]

*Triisopropyl(oct-3-yn-1-yloxy)silane (**S10**)*

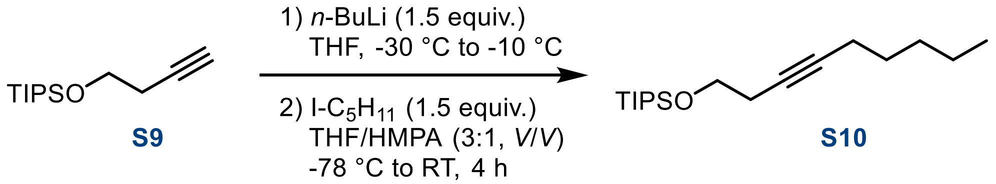

A solution of TIPS acetylene **S9** (10 g, 43.7 mmol, 1.0 equiv.) in THF (109 mL, 0.4 M) was cooled to -30 °C (dry ice/acetone, external temperature) and *n*-BuLi (41 mL, 64 mmol, 1.5 equiv.; 1.6 M sol. in hexane) was added dropwise. The resulting mixture was brought to -10 °C (cooling bath exchange, external temperature) and stirred for 1 hour. The whole mixture was then cooled to -78 °C (dry ice/acetone, external temperature) and a mixture of 1-iodopentane (8.7 mL, 43.7 mmol, 1.5 equiv.) in THF/HMPA = 3:1 (*V*/*V*; 88 mL) was added dropwise. The whole mixture was stirred at -78 °C for 1 h and then it was allowed to warm up to room temperature over a period of 4 h. H_2O (50 mL) was added and the whole mixture was extracted with Et_2O (3x100 mL). The combined organic layers were washed with brine (50 mL), dried over Na_2SO_4, filtered and the solvents were removed under reduced pressure. The crude product was purified by flash column chromatography (SiO_2; petroleum ether:EtOAc = 100:1) and yielded disubstituted alkyne **S10** (9.86 g, 76 %) as a colorless oil.

^{1}H NMR (500 MHz, Chloroform-*d*) δ (ppm): 3.77 (t, *J* = 7.4 Hz, 2H), 2.42 – 2.36 (m, 2H), 2.13 (tt, *J* = 7.2, 2.4 Hz, 2H), 1.48 (p, *J* = 7.0 Hz, 2H), 1.39 – 1.27 (m, 3H), 1.14 – 1.01 (m, 20H), 0.90 (t, *J* = 7.1 Hz, 3H).

^{13}C {^{1}H} NMR (126 MHz, Chloroform-*d*) δ (ppm): 81.6, 77.1, 62.9, 31.3, 28.95, 23.5, 22.5, 19,0, 18.2, 14.2, 12.2.

MS (ESI) *m/z* (%): 298 $[M+H]^+$.

HRMS (ESI) *m/z:* $[M+H]^+$ calculated for $C_{18}H_{39}OSi$: 297.2608; found: 297.2605.

R_f = 0.45 (vanillin, orange spot; petroleum ether:EtOAc = 10:1)

*(Z)-triisopropyl(non-3-en-1-yloxy)silane (**S11**)*

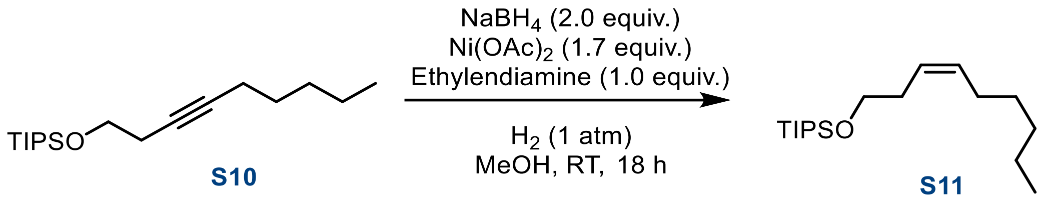

$NaBH_4$ (9.86 g, 65.8 mmol, 2.0 equiv.) was added to a stirred solution of $Ni(OAc)_2$ (14.2 g, 56 mmol, 1.7 equiv.) in dry MeOH (330 mL, 0.1 M) at room temperature and the whole mixture was kept under argon atmosphere. Argon atmosphere (balloon) was replaced with hydrogen (double layer balloon) and ethylenediamine (3.29 mL, 32.9 mmol, 10 M, 1.0 equiv.) was added. After 5 min, alkyne **S10** (9.86 g, 32.9 mmol, 1.0 equiv.) in MeOH (20 mL) was added via cannula, and the resulting mixture was stirred for 4 h. The whole mixture was filtered through a pad of Celite®, and the filter cake was washed with Et_2O (200 mL). Combined filtrates were diluted with brine (150 mL), and EtOAc (150 mL), and the resulting layers were separated. The aqueous phase was extracted with EtOAc (3x150 mL), and the combined organic layers were washed with brine (50 mL), dried over $MgSO_4$, filtered, and organic solvents were removed under reduced pressure. The crude product was purified by flash column chromatography (SiO_2; petroleum ether:EtOAc = 100:1) and yielded an alkene **S11** (8.25 g, 84 %, *E/Z* = 1:>99) in the form of colorless oil.

^{1}H NMR (500 MHz, Chloroform-*d*) δ (ppm): 5.48 – 5.35 (m, 2H), 3.68 – 3.65 (m, 2H), 2.30 (q, *J* = 7.2 Hz, 2H), 2.04 (q, *J* = 7.3 Hz, 2H), 1.35 – 1.27 (m, 6H), 1.06 (dd, *J* = 5.5, 2.3 Hz, 21H), 0.90 – 0.86 (m, 3H).

^{13}C{^{1}H} NMR (126 MHz, Chloroform-*d*) δ (ppm): 132.0, 125.7, 63.4, 31.7, 31.4, 29.6, 27.5, 22.7, 18.2, 14.2, 12.2.

MS (ESI) *m/z* (%): 300 $[M+H]^+$.

HRMS (ESI) *m/z:* $[M+H]^+$ calculated for $C_{18}H_{39}OSi$: 299.2765; found: 299.2768.

R_f = 0.55 (PMA, petroleum ether:EtOAc = 10:1)

*(Z)-non-3-en-1-ol (**4**)*

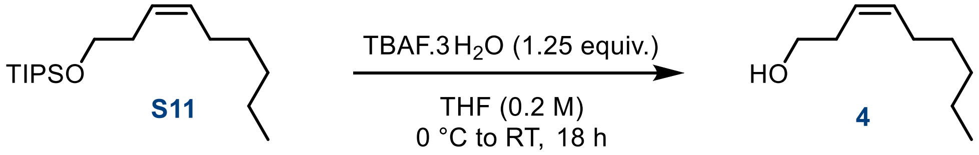

Alkene **S11** (5.0 g, 16.6 mmol, 1.0 equiv.) was dissolved in THF (83 mL, 0.2 mol/L) and the resulting mixture was cooled to 0 °C (ice/water, external temperature). SolidTBAF.3H_2O (6.67 g, 20.7 mmol, 1.25 equiv.) was

added dropwise and the resulting mixture was stirred at 0 °C for 3 h. Sat. aq. NH_4Cl (50mL) was added, and the resulting mixture was extracted with EtOAc (3x100 mL). Combined organic layers were washed with brine (50 mL), dried over $MgSO_4$, filtered and the solvents were removed under reduced pressure. The crude product was purified by column flash column chromatography (SiO_2; petroleum ether:EtOAc = 10:1) and gave alcohol **4** (2.3 g; 97 %) in form of colorless oil.

^{1}H NMR (500 MHz, Chloroform-*d*) δ (ppm): 5.62 – 5.51 (m, 1H), 5.42 – 5.29 (m, 1H), 3.67 – 3.57 (m, 2H), 2.33 (qd, *J* = 6.6, 1.5 Hz, 2H), 2.06 (qd, *J* = 7.3, 1.6 Hz, 2H), 1.39 – 1.23 (m, 7H), 0.88 (t, *J* = 6.9 Hz, 3H).

^{13}C {^{1}H} NMR (126 MHz, Chloroform-*d*) δ (ppm): 133.8, 125.1, 62.5, 31.6, 30.9, 29.5, 27.5, 22.7, 18.7, 14.2.

MS (ESI) *m/z* (%): 143 $[M+H]^+$.

HRMS (ESI) *m/z:* $[M+H]^+$ calculated for $C_9H_{19}O$: 143.1430; found: 1431429.

R*f* = 0.3 (vanillin, blue-green spot; petroleum ether:EtOAc = 4:1)

*(Z)-1-iodonon-3-ene (**S12**)*

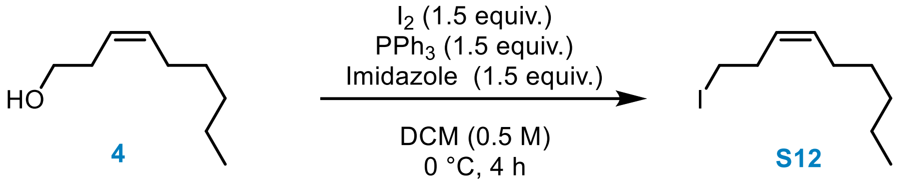

Reaction flasks must be covered in aluminum foil to protect the reaction mixture against the sunlight.

A solution of **4** (2.11 g, 14.7 mmol, 1.0 equiv.) in CH_2Cl_2 (147 mL, 0.1 M) was cooled to 0 °C (ice/water, external temperature), and PPh_3 (5.84 g, 22 mmol, 1.5 equiv.), imidazole (1.51 g, 22 mmol, 1.5 equiv.), and iodine (5.65 g, 22 mmol, 1.5 equiv.) were sequentially added. The resulting mixture was allowed to warm to room temperature, and the reaction progress was monitored by TLC (4h). Water (50 mL) was added to terminate the reaction, and the resulting phases were separated. The aqueous phase was extracted with EtOAc (3x50 mL), and the combined organic layers were washed with brine (50 mL), dried over $MgSO_4$ and the solvents were removed under reduced pressure. The crude product was purified by flash column chromatography (SiO_2; petroleum ether:EtOAc = 10:1) and yielded **S12** (3.45, 93 %) as colorless oil.

^{1}H NMR (500 MHz, Chloroform-*d*) δ (ppm): 5.57 – 5.50 (m, 1H), 5.34 – 5.28 (m, 1H), 3.13 (t, *J* = 7.3 Hz, 2H), 2.63 (q, *J* = 7.3 Hz, 2H), 2.02 (q, *J* = 7.4 Hz, 2H), 1.38 – 1.25 (m, 6H), 0.89 (t, *J* = 6.8 Hz, 3H).

^{13}C{^{1}H} NMR (126 MHz, Chloroform-*d*) δ (ppm): 132.9, 127.8, 31.7, 31.6, 29.3, 27.6, 22.7, 14.2, 5.7.

MS (ESI) *m/z* (%): 253 $[M+H]^+$.

HRMS (ESI) *m/z:* $[M+H]^+$ calculated for $C_9H_{18}I$: 253.0448; found: 253.0450.

R*f* = 0.8 (vanillin; petroleum ether:EtOAc = 10:1)

*(Z)-1-nitronon-3-ene (**1c**)*

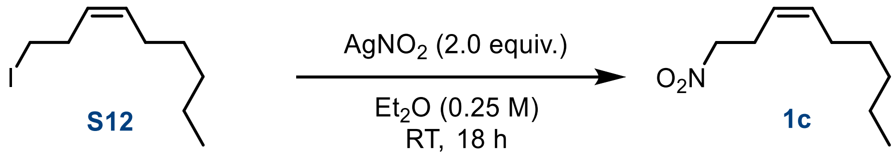

Reaction flasks must be covered in aluminum foil to protect the reaction mixture against the sunlight.

At room temperature, a silver nitrite (4.2 g, 27.1 mmol, 2.0 equiv.) was added in one portion to an aluminum foil-covered flask containing a stirred solution of **S12** (3.45 g, 13.5 mmol, 1.0 equiv.) in Et_2O (54 mL, 0.25 M). The resulting mixture was stirred at room temperature for 18 h, and then filtered through a short pad of Celite® The filter cake was washed with EtOAc (3x50 mL), and the resulting layers were separated. The aqueous layer was extracted with EtOAc (3x50 mL), and the organic layers were combined, washed with brine, dried over $MgSO_4$ and the solvents were removed under reduced pressure. The crude product was purified by flash column chromatography (SiO_2; petroleum ether:EtOAc =10:1) and yielded the desired nitrite **1c** (1.67 g, 72 %).

^{1}H NMR (500 MHz, Chloroform-*d*) *δ* (ppm): 5.58 (dtt, *J* = 16.4, 7.5, 1.5 Hz, 1H), 5.29 (dtt, *J* = 16.5, 7.5, 1.7 Hz, 1H), 4.37 (t, *J* = 7.2 Hz, 2H), 2.83 – 2.68 (m, 2H), 2.08 – 2.01 (m, 2H), 1.36 – 1.25 (m, 6H), 0.89 (t, *J* = 6.9 Hz, 3H).

^{13}C {^{1}H} NMR (126 MHz, Chloroform-*d*) *δ* (ppm): 135.1, 122.2, 75.3, 31.6, 29.2, 27.4, 25.6, 22.7, 14.2.

MS (ESI) *m/z* (%): 172 $[M+H]^+$.

HRMS (ESI) *m/z:* $[M+H]^+$ calculated for $C_9H_{18}NO_2$: 172.1332; found: 172.1335.

R*f* = 0.4 (petroleum ether:EtOAc = 10:1)

*Methyl 9-oxononanoate (**2a**)*

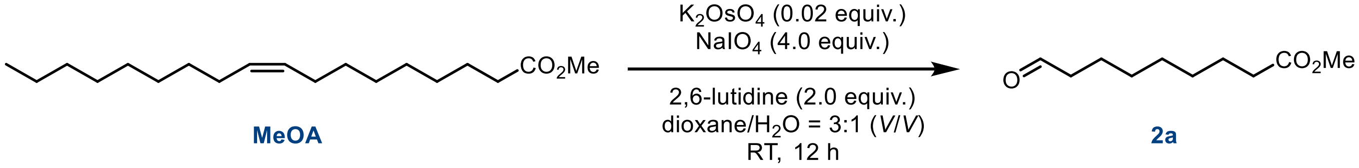

2,6-lutidine (7.83 mL, 66.8 mmol, 2.0 equiv.), K_2OsO_4 (250 mg, 0.67 mmol, 0.02 equiv.) and $NaIO_4$ (28.8 g, 134 mmol, 4.0 equiv.) were sequentially added at room temperature to a stirred solution of **MeOA** (10 g, 33.4 mmol, 1.0 equiv.) in dioxane/water = 3:1 (*V/V*, 300 mL). The reaction mixture was stirred for 16h at room temperature, filtered through a pad of Celite®, and the filter cake was washed with $CHCl_3$ (3x 200 mL). The combined filtrates were washed with water (2x150 mL), brine (150 mL), dried over $MgSO_4$ and the solvents were removed under reduced pressure. The crude product was purified by flash column chromatography (SiO_2; petroleum ether:EtOAc = 1000:1) and gave aldehyde **2a** (4.36 g, 70 %) in the form of a colorless oil.

^{1}H NMR (500 MHz, Chloroform-*d*) *δ* (ppm): 9.76 (s, 1H), 3.66 (s, 3H), 2.42 (td, *J* = 7.3, 1.7 Hz, 2H), 2.30 (t, *J* = 7.5 Hz, 2H), 1.66 – 1.59 (m, 4H), 1.35 – 1.30 (m, 6H).

^{13}C {^{1}H} NMR (126 MHz, Chloroform-*d*) δ (ppm): 203.0, 174.4, 67.2, 60.6, 51.6, 44.0, 34.2, 29.1, 29.1, 29.0, 25.0, 22.1, 14.3.

R_f = 0.7 (PMA; petroleum ether:EtOAc = 10:1)

Data matched to those previously reported.[3]

*Methyl (Z)-9-hydroxy-10-nitrooctadec-12-enoate (**3c**)*

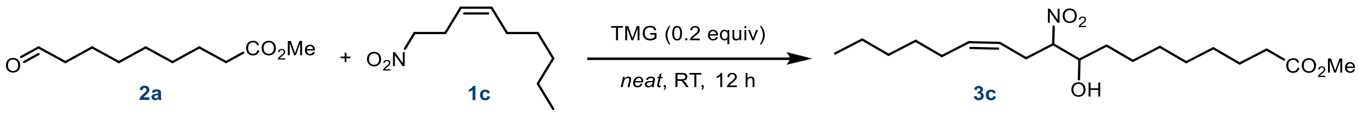

Neat mixture of nitroalkane **1c** (0.3 g, 1.73 mmol, 1.0 equiv.) and aldehyde **2a** (0.49 g, 2.6 mmol, 1.2 equiv.) was cooled to 0 °C (ice/water; external temperature) and 1,1,3,3-tetramethylguanidine (**TMG**) (0.04 g, 0.35 mmol, 0.2 equiv.) was added. The resulting mixture was stirred for 12 h at room temperature before it was cooled back to 0 °C (ice/water; external temperature), and the reaction was terminated with the addition of H_2O (5 mL). The resulting mixture was extracted with EtOAc (3×15 mL), and combined organic layers were dried over $MgSO_4$. Organic solvents were removed under reduced pressure, and the crude product was purified by flash column chromatography (SiO_2; petroleum ether:EtOAc = 50:1) and yielded the adduct **3c** (0.5 g, 81 %; *d.r.* = 1.2:1). Adduct **3c** was obtained as a mixture of two diastereoisomers in a 1.2:1 *d.r.* ratio according to the ^{1}H NMR spectra analysis.

^{1}H NMR (500 MHz, Chloroform-*d*) δ (ppm): 5.62 – 5.53 (m, 1H), 5.33 – 5.24 (m, 1H), 4.48 – 4.39 (m, 1H), 4.05 (dq, *J* = 8.5, 4.2 Hz, 0.5H, *major diastereoisomer*), 3.91 – 3.85 (m, 0.5H, *minor diastereoisomer*), 3.66 (s, 3H), 2.94 – 2.76 (m, 1H), 2.62 – 2.52 (m, 1H), 2.36 (d, *J* = 4.8 Hz, 0.5H), 2.30 (t, *J* = 7.5 Hz, 2H), 2.21 (d, *J* = 8.2 Hz, 0.5H), 2.02 (q, *J* = 7.4 Hz, 2H), 1.62 (p, *J* = 7.3 Hz, 2H), 1.58 – 1.39 (m, 4H), 1.34 – 1.26 (m, 12H), 0.88 (t, *J* = 6.9 Hz, 3H).

^{13}C {^{1}H} NMR (126 MHz, Chloroform-*d*) δ (ppm): 174.4, 135.5, 135.2, 122.3, 121.6, 92.3, 92.0, 72.2, 71.7, 51.6, 34.2, 33.7, 33.3, 31.6, 29.25, 29.22, 29.17, 29.07, 28.7, 27.4, 26.3, 25.6, 25.4, 25.0, 22.7, 14.2.

MS (ESI) *m/z* (%): 359 $[M+H]^+$.

HRMS (ESI) *m/z:* $[M+H]^+$ calculated for $C_{19}H_{36}NO_5$: 358.2588; found: 358.2590.

R_f = 0.2 (PMA; petroleum ether:EtOAc = 10:1)

*Methyl (9E,12Z)-10-nitrooctadeca-9,12-dienoate (**10-NO_2MeLA**)*

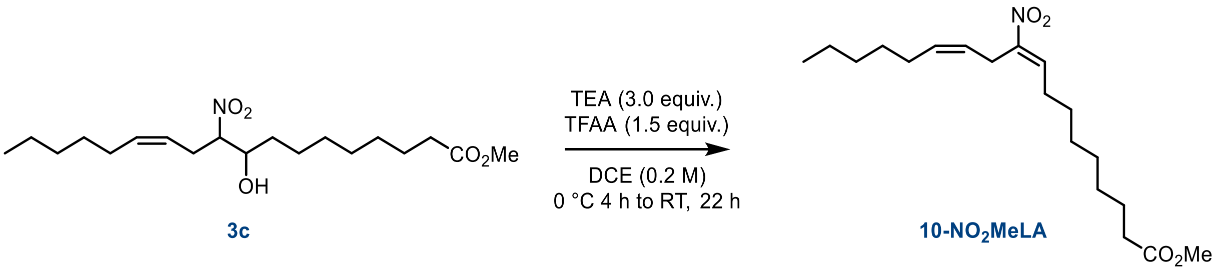

Adduct **3c** (0.5 g, 1.4 mmol, 1.0 equiv.) was dissolved in DCE (7 mL, 0.2 M) and cooled to 0 °C (ice/water, external temperature). Et_3N (0.59 mL, 4.2 mmol, 3.0 equiv.) and TFAA (0.3 mL, 2.1 mmol, 1.5 equiv.) were sequentially added, and the resulting mixture was stirred for 4 h at 0 °C, and then for 22h at room temperature.

The whole mixture was cooled to 0 °C (ice/water, external temperature), and H_2O (10 mL) was added. The resulting mixture was extracted with EtOAc (3×25 mL), and combined organic phases were dried over $MgSO_4$. Organic solvents were removed under reduced pressure, and the residue was purified by flash column chromatography (SiO_2; petroleum ether:EtOAc = 20:1) and yielded the desired nitro olefin **10-NO₂MeLA** (0.26 g, 55 %; *E/Z* ≥ 99:1 based on the ^{1}H NMR spectra analysis) in a form of a colorless oil.

^{1}H NMR (500 MHz, Chloroform-*d*) δ (ppm): 7.08 (t, J = 7.9 Hz, 1H), 5.49 (dtt, J = 10.8, 7.3, 1.8 Hz, 1H), 5.25 (dtt, J = 10.5, 7.0, 1.7 Hz, 1H), 3.66 (s, 3H), 3.33 (dd, J = 7.0, 1.8 Hz, 2H), 2.30 (t, J = 7.5 Hz, 2H), 2.24 (q, J = 7.7 Hz, 2H), 2.12 (qd, J = 7.3, 1.6 Hz, 2H), 1.62 (dd, J = 9.9, 4.6 Hz, 2H), 1.49 (dt, J = 11.5, 7.2 Hz, 2H), 1.41 – 1.23 (m, 12H), 0.91 – 0.87 (m, 3H).

^{13}C {^{1}H} NMR (126 MHz, Chloroform-*d*) δ (ppm): 174.3, 150.8, 136.6, 133.2, 123.4, 51.6, 34.1, 31.7, 29.29, 29.23, 29.12, 29.10, 28.5, 28.1, 27.5, 25.0, 22.7, 14.2.

MS (ESI) *m/z* (%): 340 $[M+H]^+$ (100).

HRMS (ESI) *m/z:* $[M+H]^+$ calculated for $C_{19}H_{34}NO_4$: 340.2482; found: 340.2486.

*(9E,12Z)-10-nitrooctadeca-9,12-dienoic acid (**10-NO₂LA**)*

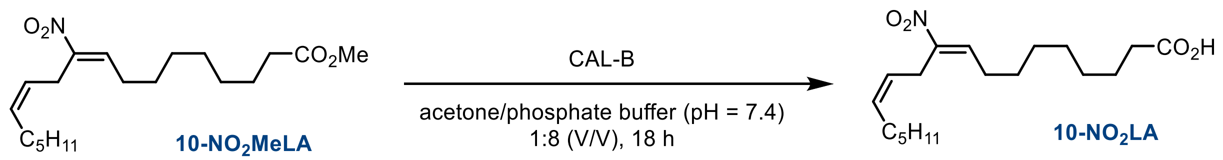

CAL-B (400 mg) was added to a solution of methyl ester **10-NO₂MeLA** (0.3 g, 0.24 mmol, 1.0 equiv.) in acetone (11 mL, 0.08 M) and aqueous phosphate buffer (88 mL, 0.01 M, pH 7.4). The solution was vigorously stirred (magnetic stirrer, 1000 rpm) for 18 h at room temperature. The pH of the mixture was adjusted to pH = 3 by 1 M aq. HCl, and the whole reaction mixture was extracted with EtOAc (5x20 mL). Combined organic layers were dried over $MgSO_4$, filtered, and the organic solvents were removed under reduced pressure. The residue was purified by semipreparative HPLC chromatography (C18 reverse-phase column; MeOH:H_2O) to yield the desired acid **10-NO₂LA** (0.12 g, 42 %, *E:Z/Z:Z* = ≥99:1 based on the ^{1}H NMR spectra analysis).

^{1}H NMR (500 MHz, Chloroform-*d*) δ (ppm): 11.14 (bs, 1H), 7.08 (t, *J* = 7.9 Hz, 1H), 5.49 (dtt, *J* = 10.9, 7.3, 1.8 Hz, 1H), 5.25 (dtt, *J* = 10.7, 6.9, 1.7 Hz, 1H), 3.34 (dd, *J* = 7.0, 1.8 Hz, 2H), 2.35 (t, *J* = 7.4 Hz, 2H), 2.24 (q, *J* = 7.6 Hz, 2H), 2.12 (q, *J* = 7.3 Hz, 2H), 1.65 – 1.61 (m, 2H), 1.53 – 1.47 (m, 2H), 1.38 – 1.29 (m, 12H), 0.90 (t, *J* = 6.8 Hz, 3H).

^{13}C {^{1}H} NMR (126 MHz, Chloroform-*d*) δ (ppm): 179.6, 150.8, 136.6, 133.2, 123.4, 34.0, 31.7, 29.3, 29.2, 29.1, 29.0, 28.5, 28.1, 27.6, 25.0, 24.7, 22.7, 14.2.

MS (ESI) *m/z* (%): 324 $[M-H]^-$ (100).

HRMS (ESI) *m/z:* $[M-H]^-$ calculated for $C_{18}H_{30}NO_4$: 324.2169; found: 324.2179.

9-NO₂cLA synthesis

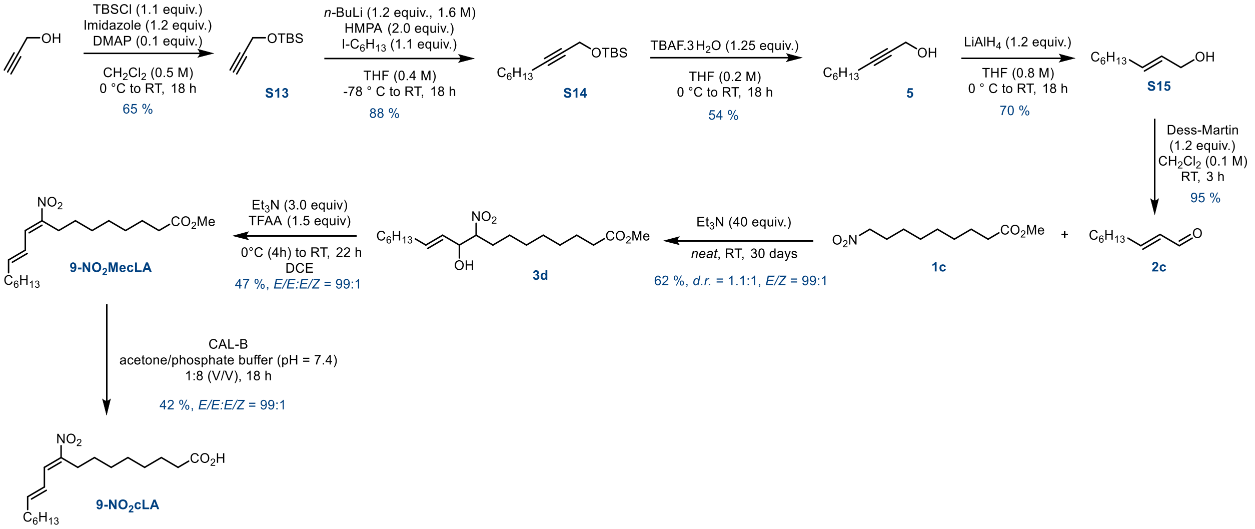

*tert-butyldimethyl(prop-2-yn-1-yloxy)silane (**S13**)*

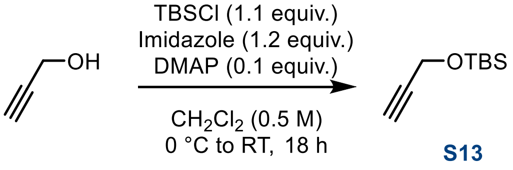

A solution of imidazole (22.0 g, 323 mmol, 1.2 equiv.) in CH_2Cl_2 (539 mL, 0.5 M) was cooled to 0 °C (ice/water, external temperature) and propargyl alcohol (15.1 g, 269 mmol, 1.0 equiv.) was added. DMAP (3.36 g, 26.9 mmol, 0.1 equiv.) and TBSCl (51.3 mL, 296 mmol, 1.1 equiv.) were added and the whole mixture was stirred for 18h at room temperature. Sat. aq. NH_4Cl (150 mL) was added, and the resulting layers were separated. The aqueous layer was extracted with CH_2Cl_2 (3x150 mL) and combined organic layers were washed with brine (100 mL), dried over Na_2SO_4, filtered, and concentrated under reduced pressure. Crude product was purified by flash column chromatography (SiO_2; petroleum ether:EtOAc = 100:1) and yielded TBS-ether **S13** (30.0 g, 65 %) in the form of a colorless oil.

^{1}H NMR (500 MHz, Chloroform-*d*) *δ* (ppm): 4.31 (m, 2H), 2.39 (td, *J* = 2.4, 0.8 Hz, 1H), 0.91 (m, 9H), 0.13 – 0.12 (m, 6H).

^{13}C {^{1}H} NMR (126 MHz, Chloroform-*d*) *δ* (ppm): 82.5, 73.0, 51.7, 26.1, 25.9, 18.4, -5.1.

MS (ESI) *m/z* (%): 171 $[M+H]^+$ (100).

R*f* = 0.55 (hexane:EtOAc, 10:1)

*tert-butyldimethyl(oct-2-yn-1-yloxy)silane (**S14**)*

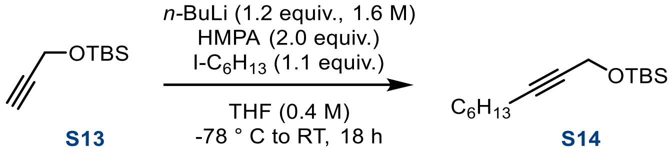

A solution of TBS protected alcohol **S13** (6.85 g, 40 mmol, 1.0 equiv.) in THF (100 mL, 0.4 M) was cooled to -78 °C (dry ice/acetone, external temperature) and *n*-BuLi (29.9 mL, 47.8 mmol, 1.6 M in hexane) was added dropwise. The resulting mixture was stirred at -78 °C for 1 h, and 1-iodohexane (6.46 mL, 43.8 mmol, 1.1 equiv.) followed by HMPA (13.9 mL, 79.6 mmol, 2.0 equiv.) were added. The reaction mixture was allowed to warm to room temperature and stirred for 18h. Sat. aq. NH_4Cl (100 mL) was added at RT and the resulting layers were separated. The aqueous phase was extracted by EtOAc (3x100 mL) and combined organic layers were dried over $MgSO_4$, filtered and concentrated under reduced pressure. The crude product was purified by flash column chromatography (SiO_2; petroleum ether:EtOAc = 100:1) and yielded a product **S14** (8.45 g, 88 %) in the form of a colorless oil.

^{1}H NMR (500 MHz, Chloroform-*d*) *δ* (ppm): 4.30 (t, *J* = 2.2 Hz, 2H), 2.19 (tt, *J* = 7.1, 2.2 Hz, 2H), 1.53 – 1.45 (m, 2H), 1.42 – 1.34 (m, 2H), 1.33 – 1.24 (m, 4H), 0.91 (d, *J* = 0.5 Hz, 9H), 0.88 (t, *J* = 7.0 Hz, 3H), 0.12 (d, *J* = 0.5 Hz, 6H).

^{13}C {^{1}H} NMR (126 MHz, Chloroform-*d*) *δ* (ppm): 85.7, 78.7, 52.2, 31.5, 28.7, 26.0, 25.9, 22.7, 18.9, 18.5, 14.2, -5.0.

MS (ESI) *m/z* (%): 256 $[M+H]^+$.

*non-2-yn-1-ol (**5**)*

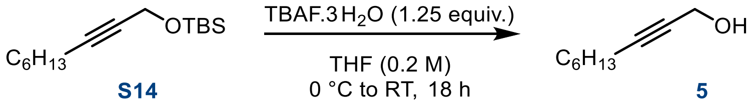

Alkyne **S14** (8.45 g, 32.9 mmol, 1.0 equiv.) was dissolved in THF (164 mL, 0.2 M) and the resulting mixture was cooled to 0 °C (ice/water, external temperature). Solid TBAF.3H_2O (13.2 g, 41.1 mmol, 1.25 equiv.) was added and the resulting mixture was stirred at 0 °C for 3 h. Sat. aq. NH_4Cl (100 mL) was added, and the resulting mixture was extracted with EtOAc (5x50 mL). Combined organic layers were washed with brine (25 mL), dried over $MgSO_4$, filtered, and the solvents were removed under reduced pressure. The crude product was purified by column flash column chromatography (SiO_2; petroleum ether:EtOAc = 40:1) and yielded alcohol **5** (2.45 g, 54 %) as a yellowish oil.

^{1}H NMR (500 MHz, Chloroform-*d*) *δ* (ppm): 4.25 (dtd, *J* = 5.9, 2.2, 1.3 Hz, 2H), 2.21 (dddd, *J* = 7.2, 5.1, 2.2, 1.1 Hz, 2H), 1.59 – 1.55 (m, 2H), 1.54 – 1.45 (m, 3H), 1.40 – 1.34 (m, 2H), 1.33 – 1.24 (m, 4H), 0.91 (d, *J* = 1.2 Hz, 3H), 0.90 – 0.86 (m, 3H).

^{13}C {^{1}H} NMR (126 MHz, Chloroform-*d*) *δ* (ppm): 86.9, 78.4, 51.6, 31.5, 28.7, 25.8, 22.7, 18.9, 14.2.

MS (ESI) *m/z* (%): 141 $[M+H]^+$.

*(E)-non-2-en-1-ol (**S15**)*

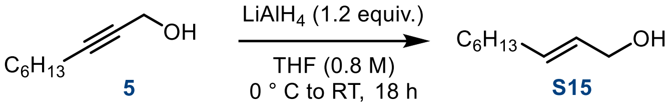

A suspension of $LiAlH_4$ (0.89 g, 23.3 mmol, 1.2 equiv.) in dry THF (24.3 mL, 0.8 M) was cooled to 0°C (ice/water, external temperature) and alcohol **5** (2.75 g, 19.4 mmol) dissolved in dry THF (19.4 mL, 1 M) was slowly added. The reaction mixture was stirred for 10 min at 0 °C and then it was allowed to warm to RT (cooling bath was removed) and stirred at RT for 18h.

Resulting mixture was again cooled to 0°C (ice/water, external temperature), and the excess of $LiAlH_4$ was destroyed by careful addition of H_2O (2 mL) and 3 M aq. NaOH (10 mL). Next, additional H_2O (3x15 mL) was added and the whole slurry was filtered. The filter cake was washed with Et_2O (5x20 mL) and CH_2Cl_2 (3x50 mL) and the resulting organic layers were collected, combined and concentrated under reduced pressure. Resulting crude product was purified by column flash chromatography (SiO_2; petroleum ether:EtOAc = 4:1) and yielded an alcohol **S15** (1.75 g, 70 %, *E/Z* = >99:1 based on the ^{1}H NMR spectra) as a yellowish oil.

^{1}H NMR (500 MHz, Chloroform-*d*) *δ* (ppm): 5.66 (qt, *J* = 15.4, 6.0 Hz, 2H), 4.08 (d, *J* = 5.5 Hz, 2H), 2.04 (q, *J* = 7.0 Hz, 2H), 1.40 – 1.33 (m, 2H), 1.32 – 1.22 (m, 6H), 0.88 (t, *J* = 6.7 Hz, 3H).

^{13}C {^{1}H} NMR (126 MHz, Chloroform-*d*) *δ* (ppm): 133.8, 128.9, 64.0, 32.4, 31.9, 29.2, 29.0, 22.8, 14.2.

MS (ESI) *m/z* (%): 143 $[M+H]^+$.

R_f = =0.69 (hexane:EtOAc, 4:1).

*(E)-non-2-enal (**2c**)*

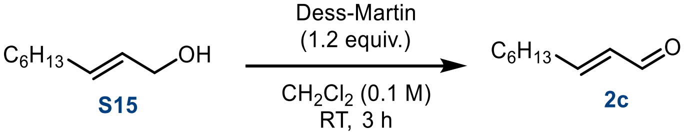

To a stirred solution of the alcohol **S15** (1.5 g, 11.7 mmol, 1.0 equiv.) in CH_2Cl_2 (117 mL, 0.1 M), Dess-Martin periodinane (6.37 g, 14.3 mmol, 1.22 equiv.) was added at RT. The resulting milky suspension was stirred for 3 h at RT. Sat. aq. $NaHCO_3$ (50 mL) was added and the whole mixture was allowed to stir for 25 min. During such time, the reaction mixture become clear and two separated phases were formed. Resulting layers were separated and the aqueous layer was extracted by CH_2Cl_2 (3x50 mL). Combined organic layers were dried over $MgSO_4$, filtered, and solvents were removed under reduced pressure. The crude product was purified by flash column chromatography (SiO_2; petroleum ether:EtOAc = 4:1) and yielded a desired product **2c** (1.5 g, 95 %, *E/Z* = >99:1 based on ^{1}H NMR spectra) as a yellowish oil.

^{1}H NMR (500 MHz, Chloroform-*d*) *δ* (ppm): 9.51 (d, *J* = 7.9 Hz, 1H), 6.85 (dt, *J* = 15.6, 6.8 Hz, 1H), 6.12 (ddt, *J* = 15.6, 7.9, 1.6 Hz, 1H), 2.38 – 2.29 (m, 2H), 1.51 (p, *J* = 7.3 Hz, 2H), 1.37 – 1.24 (m, 6H), 0.90 – 0.87 (m, 3H).

^{13}C {^{1}H} NMR (126 MHz, Chloroform-*d*) *δ* (ppm): 194.4, 159.3, 133.1, 32.9, 31.7, 28.9, 27.9, 22.7, 14.2.

HRMS (ESI) *m/z:* $[M+H]^+$ calculated for $C_9H_{16}O$: 141.1274; found: 141.1277.

*Methyl (9E,11Z)-9-nitrooctadeca-9,11-dienoate (**9-NO_2MecLA**)*

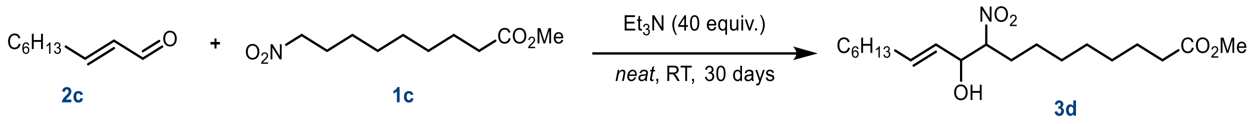

Aldehyde **2c** (0.95 g, 6.7 mmol, 1.7 equiv.) and nitro ester **2c** (0.86 g, 3.94 mmol, 1.0 equiv.) were placed to the reaction flask equipped with magnetic stirrer and Et_3N (22 mL, 158 mmol, 40 equiv.) was added. The reaction was monitored until the starting materials were consumed. After 30 days, an excess of Et_3N was removed from the reaction mixture with a stream of nitrogen, and the resulting crude product was dissolved in Et_2O (20 mL). Organic layer was placed in a separatory funnel and sequentially washed with 0.1 M aq. HCl (30×2 mL), water (30×2 mL), and brine (30 mL). The resulting organic phase was dried over Na_2SO_4, filtered through a plug of silica gel and Celite® (3g, SiO_2:Celite® = 50:50 (w/w), and the filtrate was concentrated under reduced pressure. The crude product was purified by gradient flash column chromatography (SiO_2; petroleum ether:EtOAc = 30:1 → 1:1) to yield adduct **3d** (0.873 g, 62 %, *d.r.* = 1.1:1, *E/Z* = >99:1 based on ^{1}H NMR spectra) together with reisolated aldehyde **2c** (0.2 g) and nitro ester **1c** (0.18 g). The product **3d** was then immediately used in the next step.

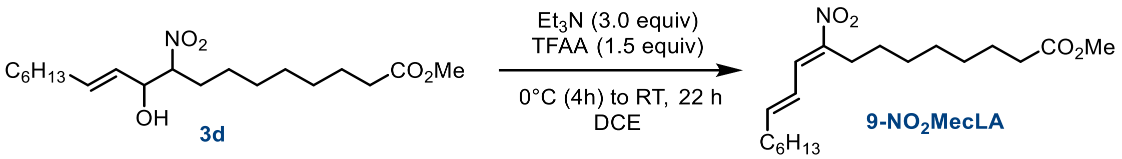

Adduct **3d** (1.15 g, 3.2 mmol, 1.0 equiv.) was dissolved in DCE (16 mL, 0.2 M) and the whole mixture was cooled to 0 °C (ice/water, external temperature). Et_3N (0.98 mL, 9.6 mmol, 3.0 equiv.) and TFAA (1.01 mL, 4.8 mmol, 1.5 equiv.) were sequentially added, and the resulting mixture was stirred at 0 °C for 4 h and then at room temperature for next 22 h.

The whole mixture was cooled to 0 °C (ice/water, external temperature) and H_2O (20 mL) was added. The resulting mixture was extracted with EtOAc (3×25 mL), and organic layers were combined, dried over $MgSO_4$, and the solvents were removed under reduced pressure. The crude product was purified by flash column chromatography (SiO_2; petroleum ether:EtOAc = 20:1) to yield the desired nitro olefin **9-NO_2MecLA** (0.54 g, 47 %; *E/Z* ≥ 99:1 based on the ^{1}H NMR spectra analysis).

^{1}H NMR (500 MHz, Chloroform-*d*) *δ* (ppm): 7.54 (d, *J* = 11.3 Hz, 1H), 6.34 (dt, *J* = 14.5, 7.0 Hz, 1H), 6.19 (ddt, *J* = 14.8, 11.4, 1.4 Hz, 1H), 3.66 (s, 3H), 2.71 – 2.60 (m, 2H), 2.30 (t, *J* = 7.5 Hz, 2H), 2.25 (q, *J* = 7.8, 7.2 Hz, 2H), 1.62 (dd, *J* = 10.1, 4.5 Hz, 2H), 1.50 (q, *J* = 6.3, 5.4 Hz, 2H), 1.50 – 1.37 (m, 2H), 1.39 – 1.22 (m, 12H), 0.89 (t, *J* = 6.8 Hz, 3H).

^{13}C {^{1}H} NMR (126 MHz, Chloroform-*d*) *δ* (ppm): 174.4, 149.5, 149.2, 134.1, 123.7, 51.6, 34.2, 33.8, 31.7, 29.1, 29.1, 29.1, 29.0, 28.7, 28.2, 26.7, 25.0, 22.7, 14.2.

HRMS (ESI) *m/z:* $[M+H]^+$ calculated for $C_{19}H_{33}NO_4$: 340.2482; found: 340.2480.

*(9E,11Z)-9-nitrooctadeca-9,11-dienoic acid (**9-NO₂cLA**)*

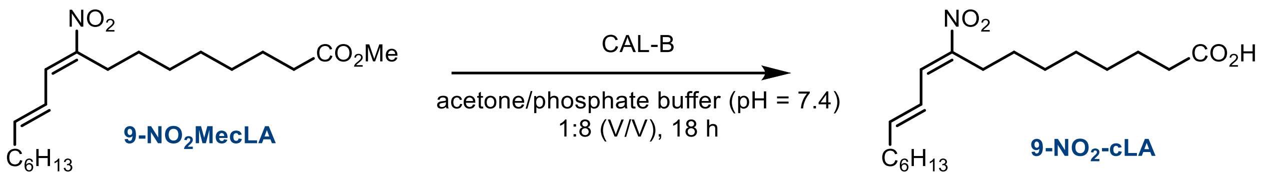

CAL-B (600 mg) was added to a solution of **9-NO$_2$MecLA** (1.115 g, 3.2 mmol, 1.0 equiv.) in acetone (11 mL, 0.08 M) and aqueous phosphate buffer (88 mL, 0.01 M, pH 7.4). The solution was vigorously stirred (magnetic stirrer, 1000 rpm) at room temperature for 18 h. The pH of the reaction mixture was adjusted with help of 1 M aq. HCl to pH = 3, and the whole mixture was extracted with EtOAc (5x20 mL). Combined organic layers were dried over $MgSO_4$, filtered, and organic solvents were removed under reduced pressure. The crude product was purified by semipreparative HPLC chromatography (C18 reverse-phase column; MeOH:H_2O) and yielded the desired acid **9-NO$_2$cLA** (0.44 g, 42 %, *E/Z* ≥ 99:1 based on the ^{1}H NMR spectra analysis).

^{1}H NMR (500 MHz, Chloroform-*d*) *δ* (ppm): 7.56 – 7.52 (m, 1H), 6.35 (dt, *J* = 14.5, 7.0 Hz, 1H), 6.19 (ddt, *J* = 15.0, 11.4, 1.4 Hz, 1H), 2.69 – 2.62 (m, 2H), 2.35 (t, *J* = 7.5 Hz, 2H), 2.27 – 2.22 (m, 2H), 1.63 (p, *J* = 7.9, 7.1 Hz, 2H), 1.52 (d, *J* = 7.3 Hz, 2H), 1.48 – 1.42 (m, 2H), 1.37 – 1.25 (m, 12H), 0.94 – 0.85 (m, 3H).

^{13}C {^{1}H} NMR (126 MHz, Chloroform-*d*) *δ* (ppm): 178.3, 149.5, 149.2, 134.1, 123.7, 33.83, 33.79, 31.8, 29.12, 29.06, 29.04, 28.99, 28.7, 28.2, 26.7, 24.7, 22.7, 14.2.

HRMS (ESI) *m/z:* [M-H]$^-$ calculated for $C_{18}H_{30}NO_4$: 324.2180; found: 324.2178.

R$_f$ = 0.33 (hexane/EtOAc; 3:1)

10-NO₂SA synthesis

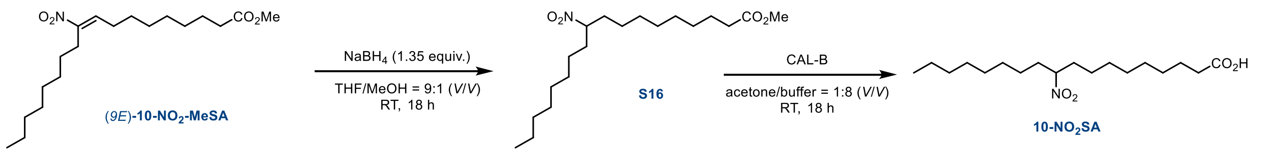

*Methyl 10-nitrooctadecanoate (**S16**)*

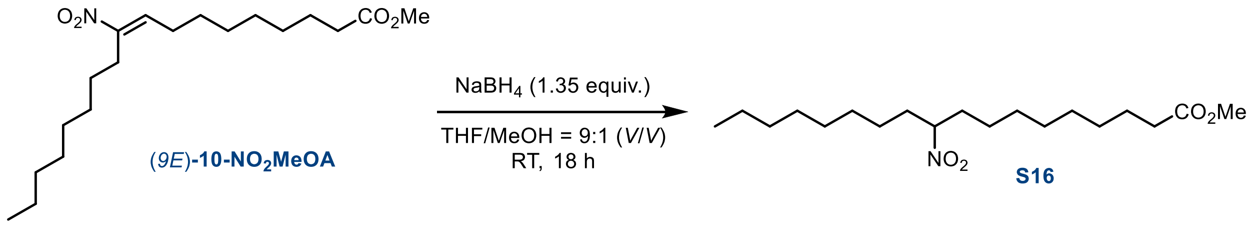

Solid $NaBH_4$ (0.058 g, 1.5 mmol, 1.35 equiv.) was added to a cold (0 °C, ice/water, external temperature) solution of (9*E*)-**10-NO$_2$MeOA** (0.400 g, 1.1 mmol, 1.0 equiv.) in THF/MeOH (5.5 mL, 9:1 (*V*/*V*)). The cooling bath was removed and the resulting mixture was stirred at RT for 14 h. H_2O (5 mL) and EtOAc (5 mL) were added and the resulting layers were separated. The aqueous layer was extracted with EtOAc (5x20 mL), and the combined organic layers were dried over $MgSO_4$, filtered, and solvents were removed under reduced pressure. The crude product was purified by flash column chromatography (SiO_2; petroleum ether:EtOAc = 10:1) and yielded the desired nitro alkane **S16** (0.276 g, 73 %).

^{1}H NMR (500 MHz, Chloroform-*d*) *δ* (ppm): 4.45 (tt, *J* = 9.3, 4.5 Hz, 1H), 3.66 (s, 3H), 2.30 (t, *J* = 7.5 Hz, 2H), 1.94 (dtd, *J* = 14.0, 8.9, 4.3 Hz, 2H), 1.67 (dp, *J* = 15.2, 5.4, 5.0 Hz, 2H), 1.59 (dt, *J* = 12.4, 6.1 Hz, 2H), 1.34 – 1.20 (m, 22H), 0.87 (t, *J* = 6.9 Hz, 3H).

^{13}C {^{1}H} NMR (126 MHz, Chloroform-*d*) *δ* (ppm): 174.5, 89.2, 51.6, 34.2, 34.1, 34.0, 31.9, 29.4, 29.3, 29.20, 29.17, 29.11, 29.0, 25.93, 25.91, 25.0, 22.8, 14.2.

MS (ESI) *m/z* (%): 345 [M+H]$^+$.

HRMS (ESI) *m/z*: [M+H]$^+$ calculated for $C_{19}H_{38}NO_4$: 344.2795; found: 344.2796.

*10-nitrooctadecanoic acid (**10-NO₂SA**)*

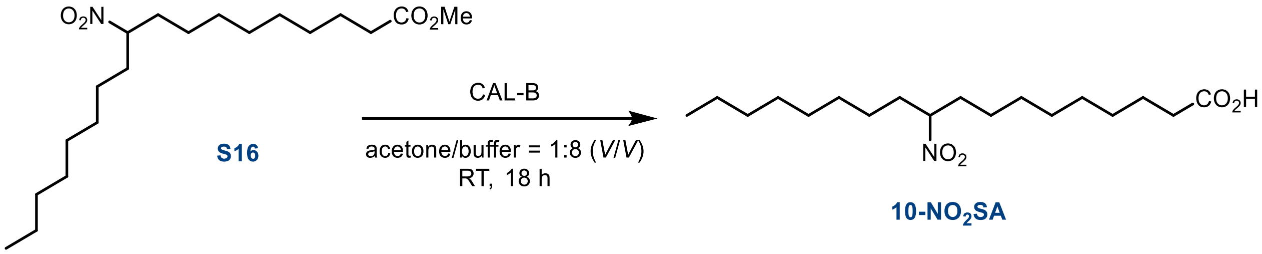

CAL-B (0.400 g) was added to a solution of methyl ester **S16** (0.18 g, 0.52 mmol, 1.0 equiv.) in acetone (6.50 mL, 0.08 mol/L) and aqueous phosphate buffer (52.4 mL, 0.01 mol/L, pH 7.4). The solution was vigorously stirred (magnetic stirrer, 1000 rpm) at room temperature for 18 h.

The pH of the reaction mixture was adjusted to pH = 3 by 1.0 M aq. HCl, and the whole mixture was extracted with EtOAc (5x20 mL). Combined organic layers were dried over $MgSO_4$, filtered, and the volatiles were removed under reduced pressure. The crude product was purified by flash column chromatography (SiO_2; petroleum ether:EtOAc = 10:1) to yield **10-NO$_2$SA** (0.17 g, 96 %) in the form of a colorless oil.

^{1}H NMR (500 MHz, Chloroform-*d*) *δ* (ppm): 11.15 (bs, 1H), 4.45 (tt, *J* = 9.3, 4.5 Hz, 1H), 2.34 (t, *J* = 7.5 Hz, 2H), 1.99 – 1.89 (m, 2H), 1.72 – 1.58 (m, 4H), 1.36 – 1.22 (m, 22H), 0.87 (t, *J* = 7.0 Hz, 3H).

^{13}C {^{1}H} NMR (126 MHz, Chloroform-*d*) *δ* (ppm): 179.7, 89.2, 34.1, 34.0, 31.9, 29.4, 29.3, 29.19, 29.17, 29.11, 29.06, 29.02, 25.93, 25.90, 24.7, 22.8, 14.2.

MS (ESI) *m/z* (%): 329 [M-H]$^-$.

HRMS (ESI) *m/z*: [M-H]$^-$ calculated for $C_{18}H_{34}NO_4$: 328.2493; found: 328.2496.

Literature

(1) Coles, B.; Bloodsworth, A.; Clark, S. R.; Lewis, M. J.; Cross, A. R.; Freeman, B. A.; O'donnell, V. B. Nitro Fatty Acids (NO2-FAs): An Emerging Class of Bioactive Fatty Acids. *Molecules* **2021**, *26* (24), 7536. https://doi.org/10.3390/MOLECULES26247536.

(2) Maity, S.; Manna, S.; Rana, S.; Naveen, T.; Mallick, A.; Maiti, D. Efficient and Stereoselective Nitration of Mono- and Disubstituted Olefins with AgNO2 and TEMPO. *J Am Chem Soc* **2013**, *135* (9), 3355–3358. https://doi.org/10.1021/JA311942E/SUPPL_FILE/JA311942E_SI_003.CIF.

(3) Woodcock, S. R.; Bonacci, G.; Gelhaus, S. L.; Schopfer, F. J. Nitrated Fatty Acids: Synthesis and Measurement. **2012**. https://doi.org/10.1016/j.freeradbiomed.2012.11.015.

(4) Hernychova, L.; Alexandri, E.; Tzakos, A. G.; Zatloukalová, M.; Primikyri, A.; Gerothanassis, I. P.; Uhrik, L.; Marekˇmarekšebela, M.; Kopečný, D.; Jedinák, L.; Vacek, J. Serum Albumin as a Primary Non-Covalent Binding Protein for Nitro-Oleic Acid. *Int J Biol Macromol* **2022**, *203*, 116–129. https://doi.org/10.1016/j.ijbiomac.2022.01.050.

(5) Mollenhauer, M.; Mehrkens, D.; Klinke, A.; Lange, M.; Remane, L.; Friedrichs, K.; Braumann, S.; Geißen, S.; Simsekyilmaz, S.; Nettersheim, F. S.; Lee, S.; Peinkofer, G.; Geisler, A. C.; Geis, B.; Schwoerer, A. P.; Carrier, L.; Freeman, B. A.; Dewenter, M.; Luo, X.; El-Armouche, A.; Wagner, M.; Adam, M.; Baldus, S.; Rudolph, V. Nitro-Fatty Acids Suppress Ischemic Ventricular Arrhythmias by Preserving Calcium Homeostasis. *Sci Rep* **2020**, *10* (1), 1–11. https://doi.org/10.1038/s41598-020-71870-6.

(6) Pereckova, J.; Pekarova, M.; Szamecova, N.; Hoferova, Z.; Kamarytova, K.; Falk, M.; Perecko, T. Nitro-oleic Acid Inhibits Stemness Maintenance and Enhances Neural Differentiation of Mouse Embryonic Stem Cells via Stat3 Signaling. *Int J Mol Sci* **2021**, *22* (18), 9981. https://doi.org/10.3390/IJMS22189981/S1.

(7) Fang, M. Y.; Huang, K. H.; Tu, W. J.; Chen, Y. T.; Pan, P. Y.; Hsiao, W. C.; Ke, Y. Y.; Tsou, L. K.; Zhang, M. M. Chemoproteomic Profiling Reveals Cellular Targets of Nitro-Fatty Acids. *Redox Biol* **2021**, *46*, 102126. https://doi.org/10.1016/J.REDOX.2021.102126.

(8) Zatloukalová, M.; Jedinák, L.; Riman, D.; Franková, J.; Novák, D.; Cytryniak, A.; Nazaruk, E.; Bilewicz, R.; Vrba, J.; Papoušková, B.; Kabeláč, M.; Vacek, J. Cubosomal Lipid Formulation of Nitroalkene Fatty Acids: Preparation, Stability and Biological Effects. *Redox Biol* **2021**, *46*, 102097. https://doi.org/10.1016/J.REDOX.2021.102097.

(9) Novák, D.; Vrba, J.; Zatloukalová, M.; Roubalová, L.; Stolarczyk, K.; Dorčák, V.; Vacek, J. Cysteamine Assay for the Evaluation of Bioactive Electrophiles. *Free Radic Biol Med* **2021**, *164*, 381–389. https://doi.org/10.1016/j.freeradbiomed.2021.01.007.

(10) Woodcock, S. R.; Marwitz, A. J. V.; Bruno, P.; Branchaud, B. P. Synthesis of Nitrolipids. All Four Possible Diastereomers of Nitrooleic Acids: (E)- and (Z)-, 9- and 10-Nitro-Octadec-9-Enoic Acids. *Org Lett* **2006**, *8* (18), 3931–3934. https://doi.org/10.1021/OL0613463/SUPPL_FILE/OL0613463SI20060720_095604.PDF.

(11) Woodcock, S. R.; Bonacci, G.; Gelhaus, S. L.; Schopfer, F. J. Nitrated Fatty Acids: Synthesis and Measurement. *Free Radic Biol Med* **2013**, *59*, 14–26. https://doi.org/10.1016/J.FREERADBIOMED.2012.11.015.

(12) Hock, K. J.; Grimmer, J.; Göbel, D.; Gasaya, G. G. T.; Roos, J.; Maucher, I. V.; Kühn, B.; Fettel, J.; Maier, T. J.; Manolikakes, G. Modular Regiospecific Synthesis of Nitrated Fatty Acids. *Synthesis (Stuttg)* **2017**, *49* (03), 615–636. https://doi.org/10.1055/S-0036-1588314.

(13) Wang, P.; Killeen, M. E.; Sumpter, T. L.; Ferris, L. K.; Falo, L. D.; Freeman, B. A.; Schopfer, F. J.; Mathers, A. R. Electrophilic Nitro-Fatty Acids Suppress Psoriasiform Dermatitis: STAT3 Inhibition as a Contributory Mechanism. *Redox Biol* **2021**, *43*, 101987. https://doi.org/10.1016/J.REDOX.2021.101987.

(14) Mollenhauer, M.; Mehrkens, D.; Klinke, A.; Lange, M.; Remane, L.; Friedrichs, K.; Braumann, S.; Geißen, S.; Simsekyilmaz, S.; Nettersheim, F. S.; Lee, S.; Peinkofer, G.; Geisler, A. C.; Geis, B.; Schwoerer, A. P.; Carrier, L.; Freeman, B. A.; Dewenter, M.; Luo, X.; El-Armouche, A.; Wagner, M.; Adam, M.; Baldus, S.; Rudolph, V. Nitro-Fatty Acids Suppress Ischemic Ventricular Arrhythmias by Preserving Calcium Homeostasis. *Sci Rep* **2020**, *10* (1), 1–11. https://doi.org/10.1038/s41598-020-71870-6.

(15) Wilkinson, M. L.; Abramova, E.; Guo, C.; Gow, J. G.; Murray, A.; Koudelka, A.; Cechova, V.; Freeman, B. A.; Gow, A. J. Fatty Acid Nitroalkenes Inhibit the Inflammatory Response to Bleomycin-Mediated Lung Injury. *Toxicol Appl Pharmacol* **2020**, *407*, 115236. https://doi.org/10.1016/J.TAAP.2020.115236.

(16) Carreño, M.; Bresque, M.; Machado, M. R.; Santos, L.; Durán, R.; Vitturi, D. A.; Escande, C.; Denicola, A. Nitro-Fatty Acids as Activators of HSIRT6 Deacetylase Activity. *Journal of Biological Chemistry* **2020**, *295* (52), 18355–18366. https://doi.org/10.1074/JBC.RA120.014883.

(17) Hellmuth, N.; Brat, C.; Awad, O.; George, S.; Kahnt, A.; Bauer, T.; Huynh Phuoc, H. P.; Steinhilber, D.; Angioni, C.; Hassan, M.; Hock, K. J.; Manolikakes, G.; Zacharowski, K.; Roos, J.; Maier, T. J. Structural Modifications Yield Novel Insights Into the Intriguing Pharmacodynamic Potential of Anti-Inflammatory Nitro-Fatty Acids. *Front Pharmacol* **2021**, *12*, 715076. https://doi.org/10.3389/FPHAR.2021.715076/BIBTEX.

(18) Hassan, M.; Krieg, S. C.; Ndefo Nde, C.; Roos, J.; Maier, T. J.; El Rady, E. A.; Raslan, M. A.; Sadek, K. U.; Manolikakes, G. Streamlined One-Pot Synthesis of Nitro Fatty Acids. *European J Org Chem* **2021**, *2021* (15), 2239–2252. https://doi.org/10.1002/ejoc.202100247.

(19) Woodcock, S. R.; Salvatore, S. R.; Freeman, B. A.; Schopfer, F. J. Synthesis of 9- and 12-Nitro Conjugated Linoleic Acid: Regiospecific Isomers of Naturally Occurring Conjugated Nitrodienes. *Tetrahedron Lett* **2021**, *81*, 153371. https://doi.org/10.1016/J.TETLET.2021.153371.

Copy of ^{1}H and $^{13}C\{^{1}H\}$ NMR spectra

Copy of ^{1}H and $^{13}C\{^{1}H\}$ spectra of **1a**

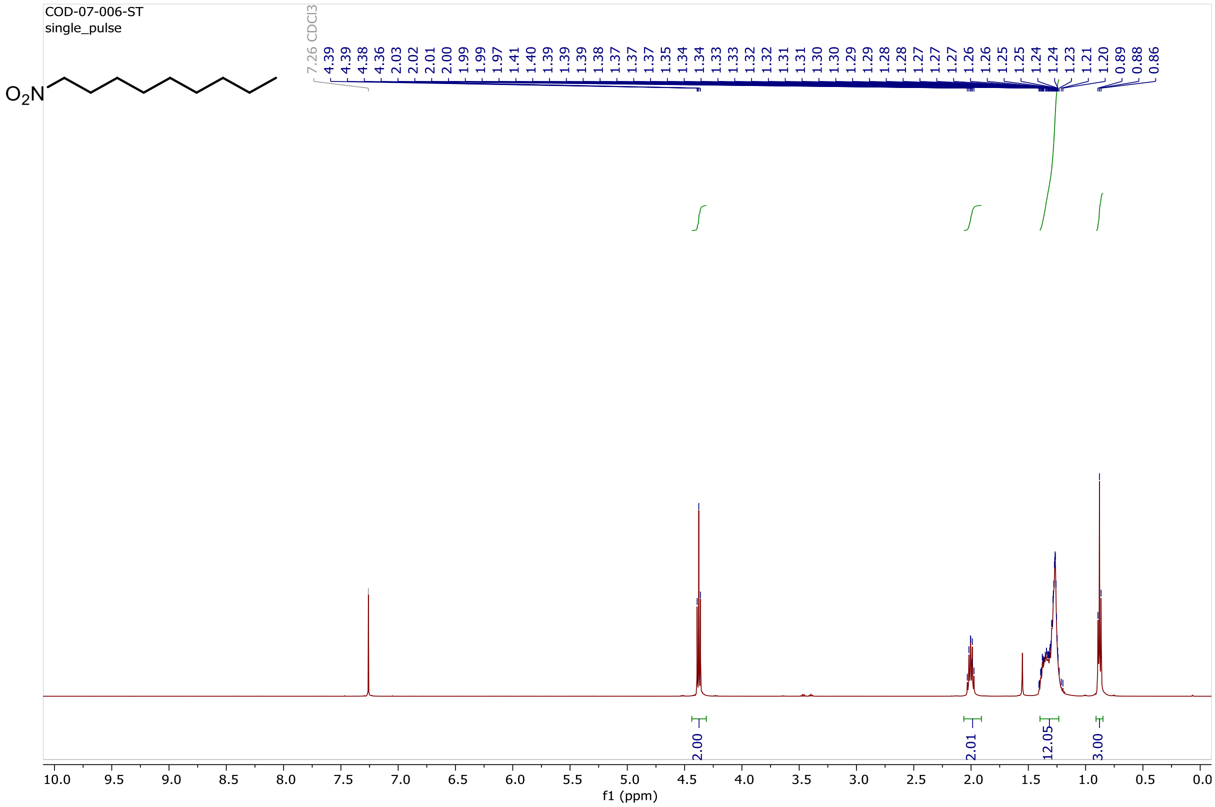

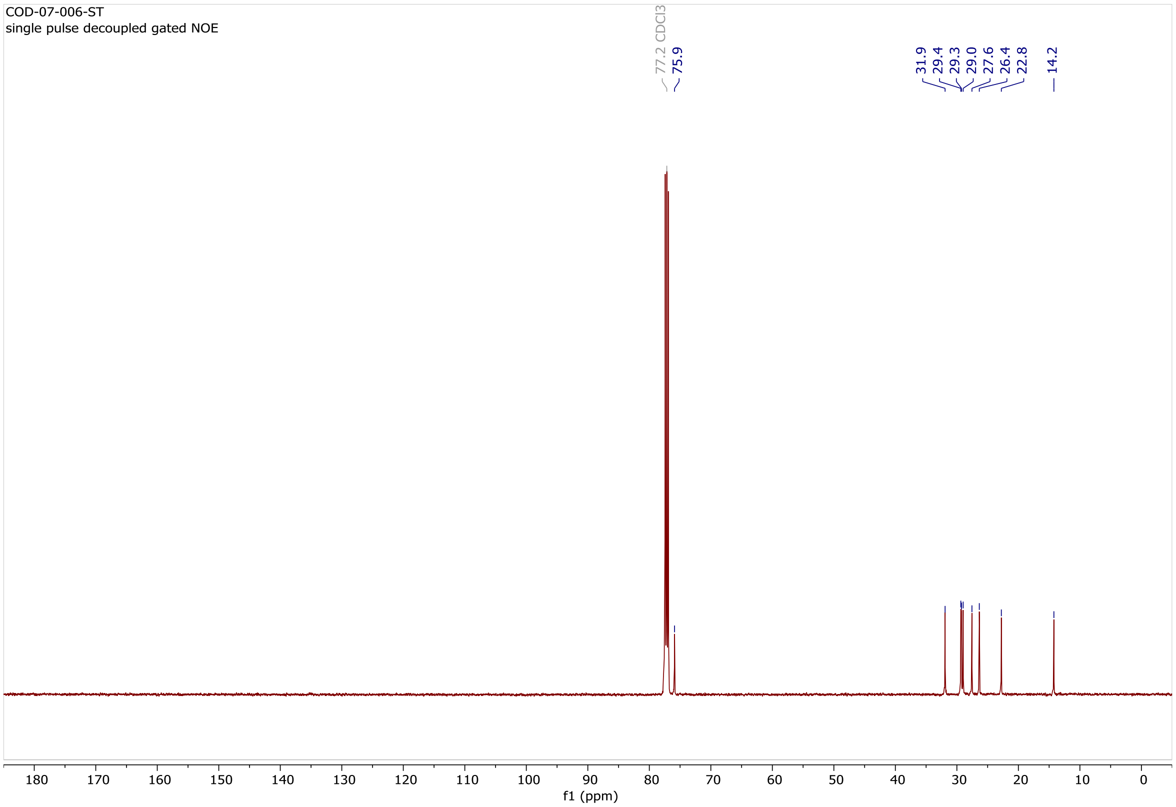

Copy of ^{1}H and ^{13}C{^{1}H} spectra of **3a**

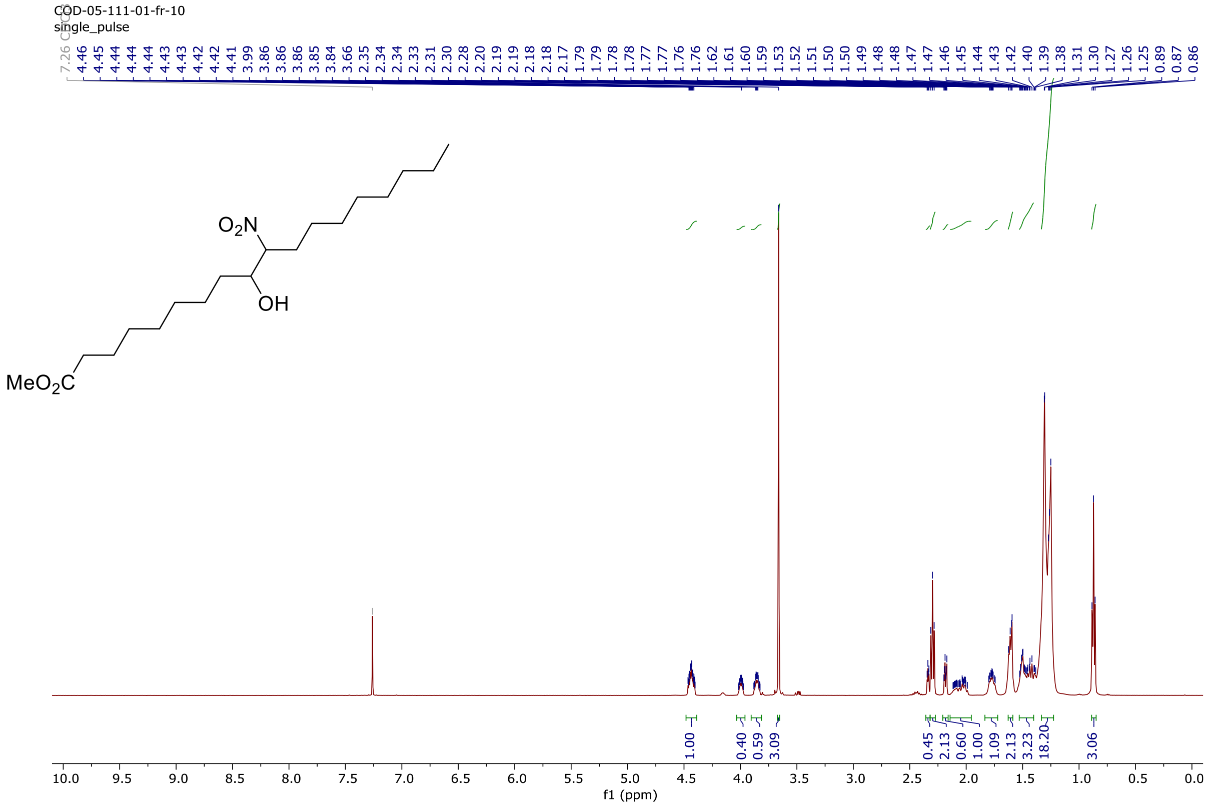

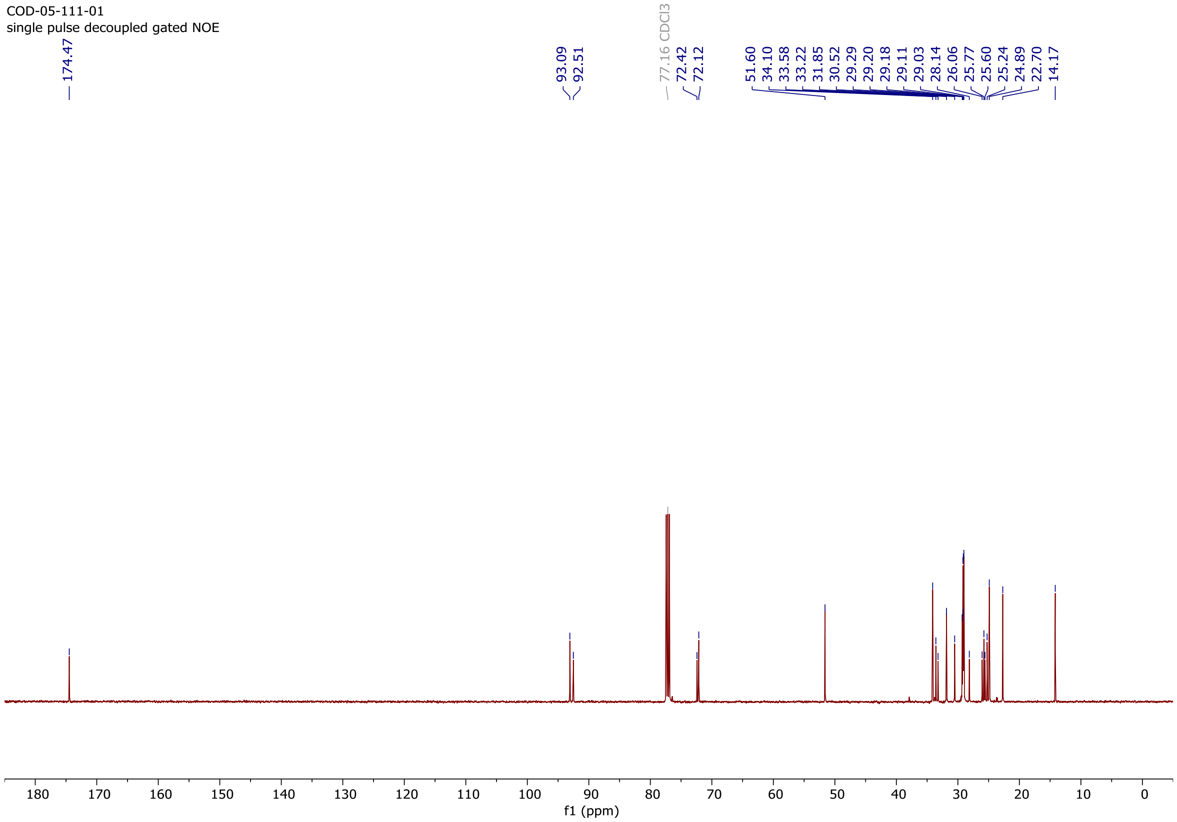

Copy of ^{1}H and $^{13}C\{^{1}H\}$ spectra of (9*E*)-**10-NO₂MeOA**

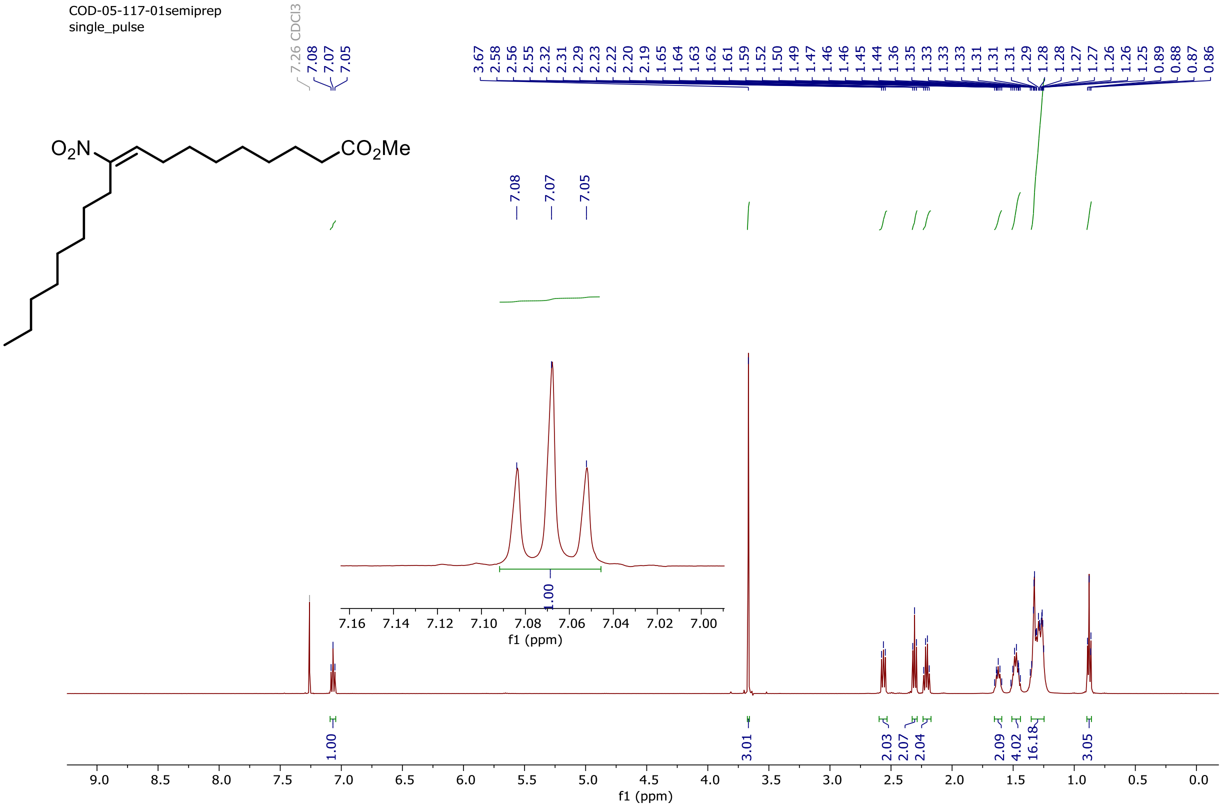

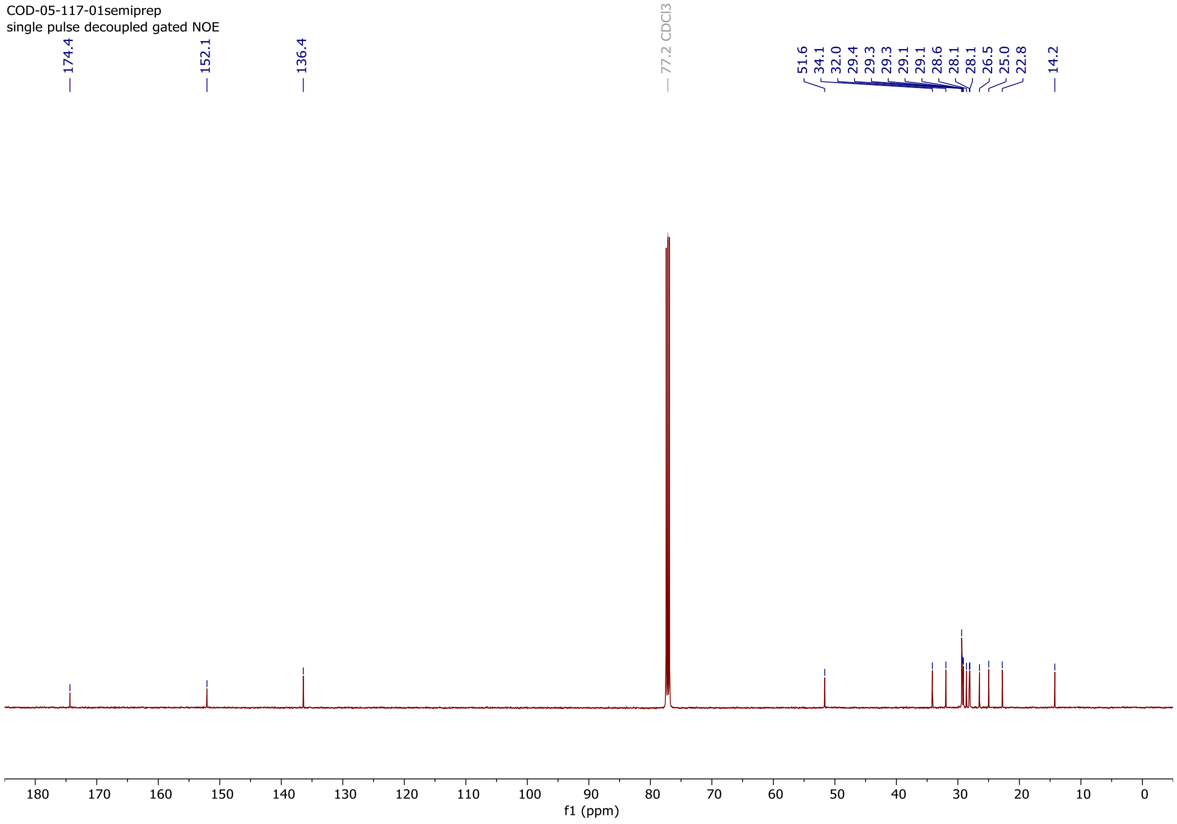

Copy of ^1H and $^{13}C\{^1H\}$ spectra of **10-NO₂OA**

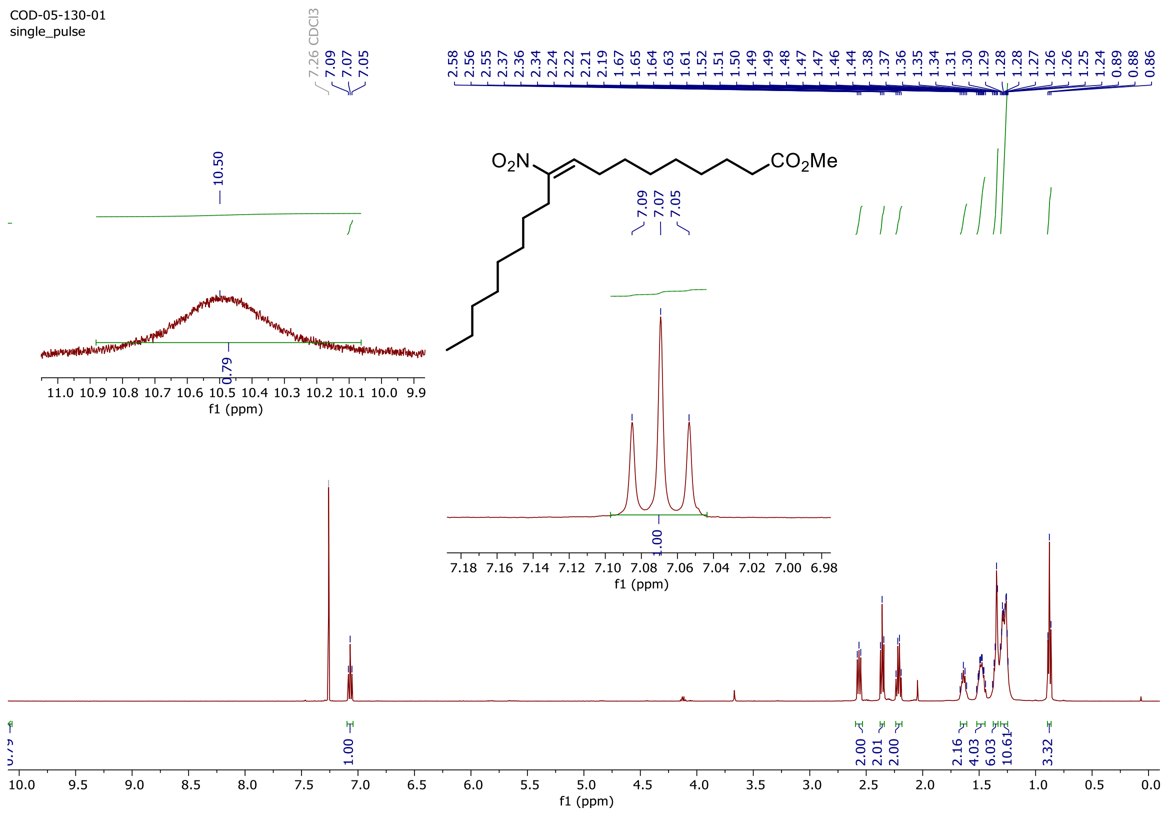

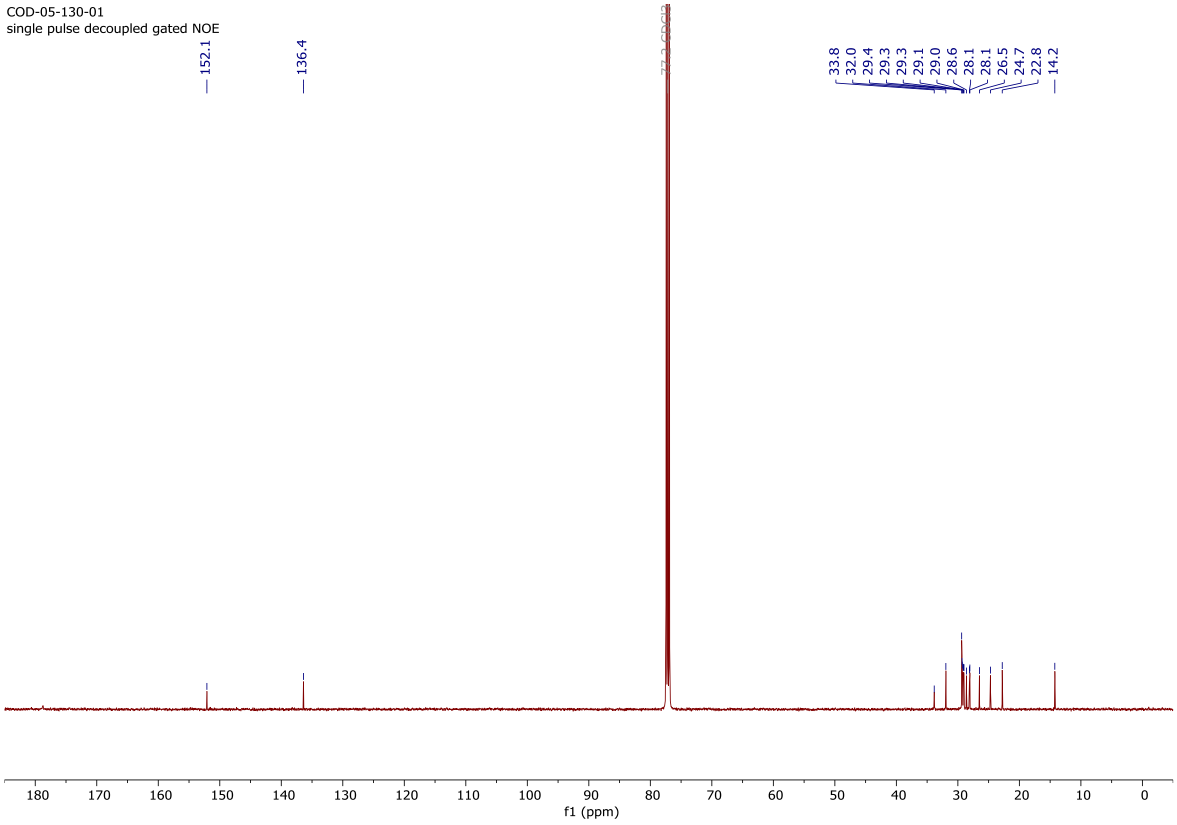

Copy of ^{1}H and $^{13}C\{^{1}H\}$ spectra of **S6**

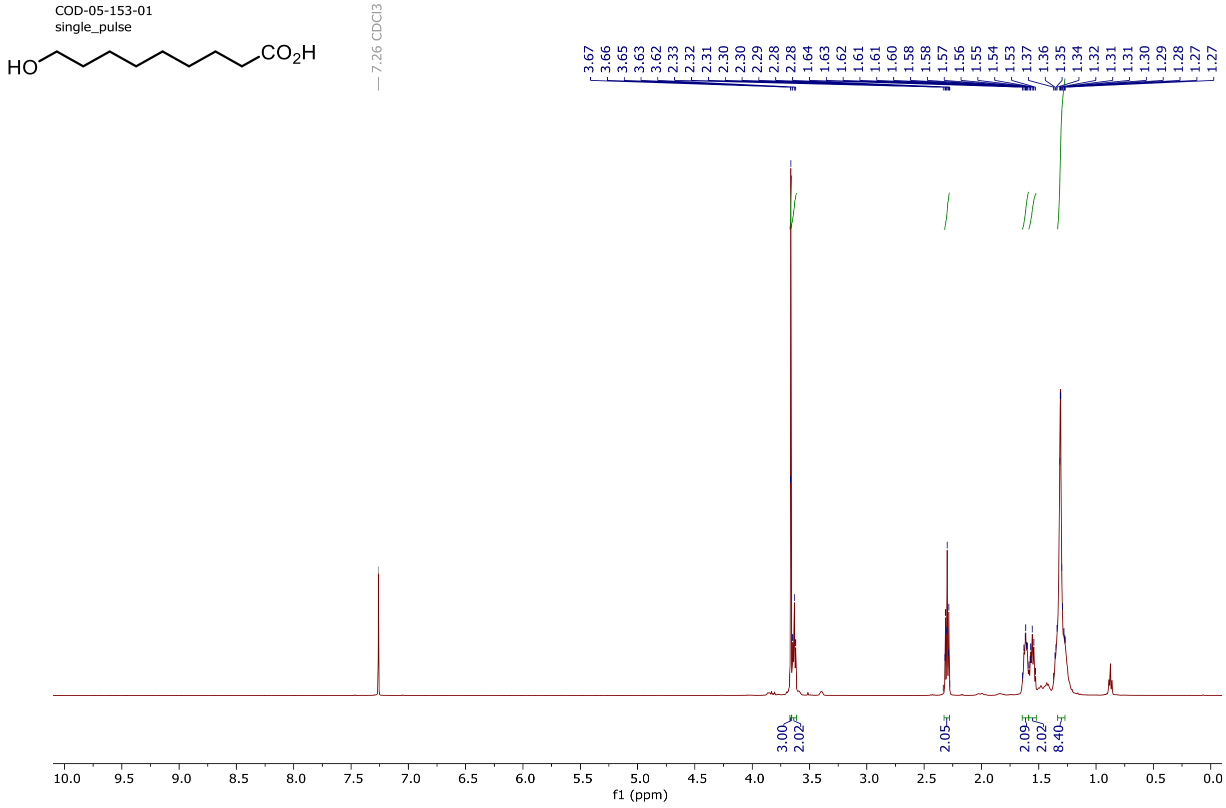

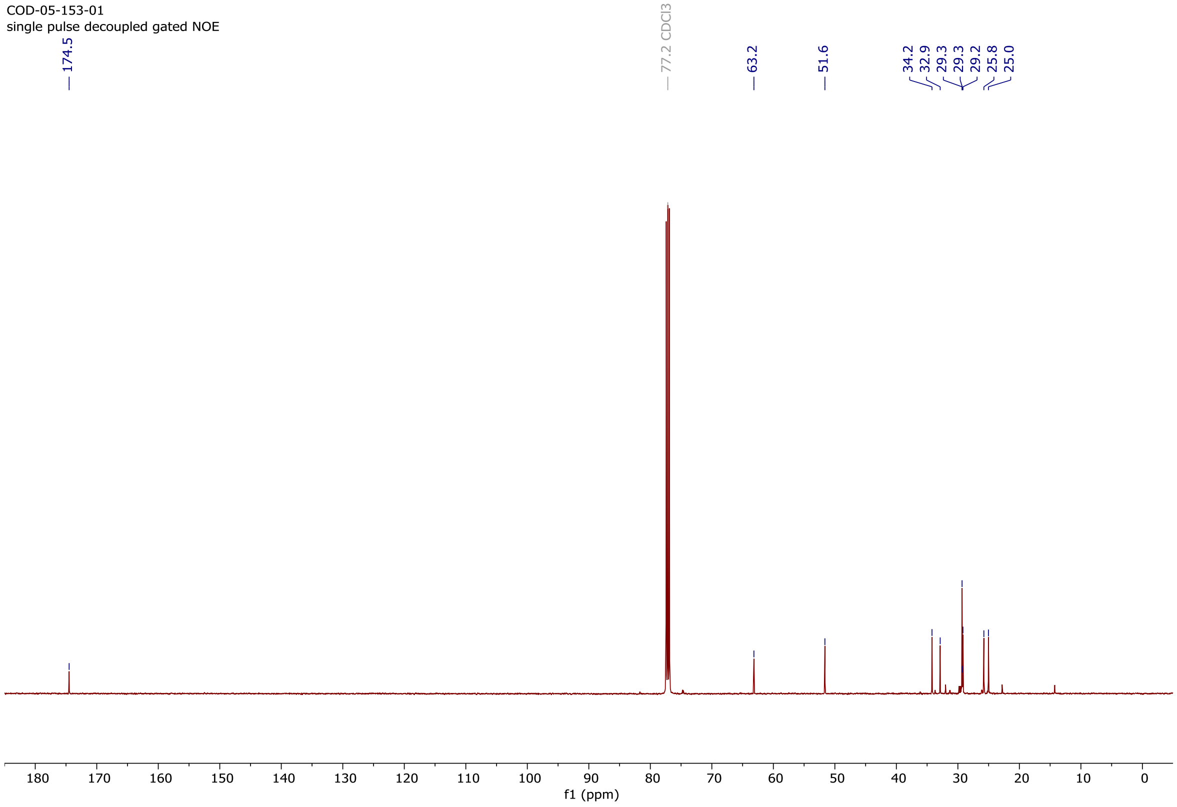

Copy of ^{1}H and $^{13}C\{^{1}H\}$ spectra of **S7**

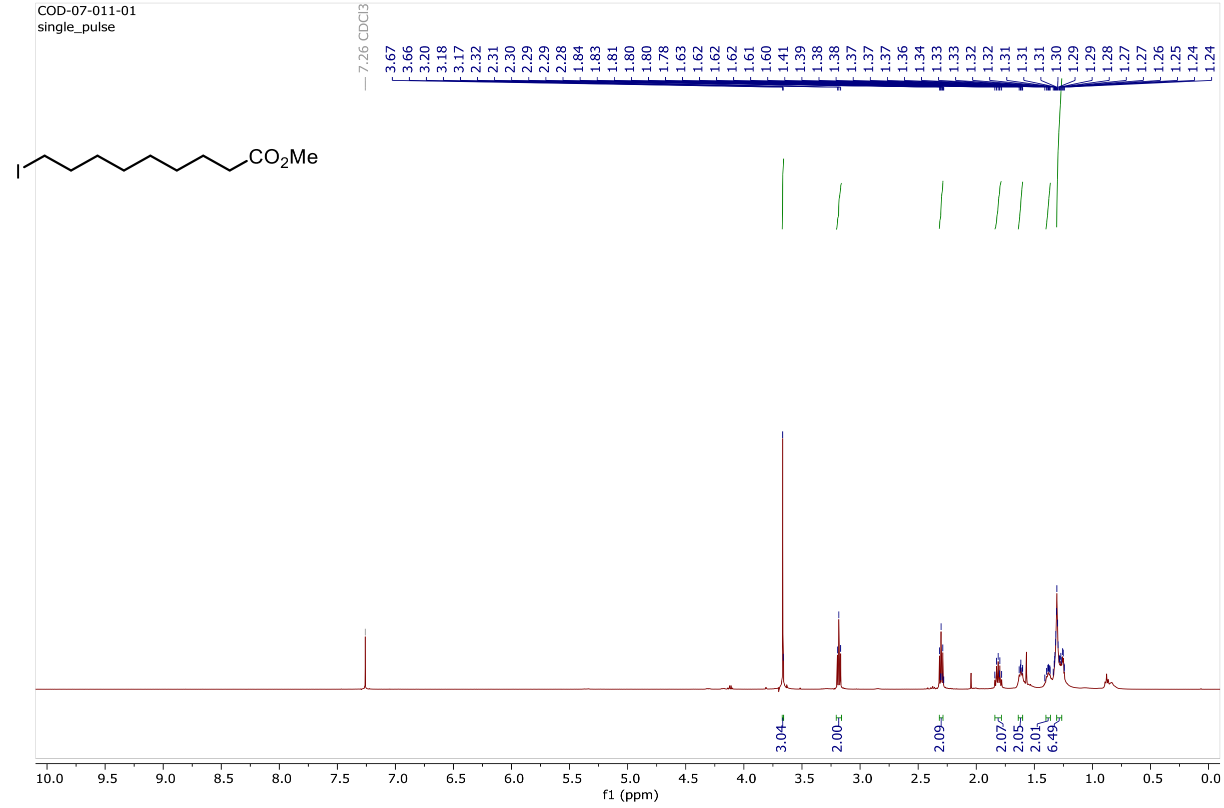

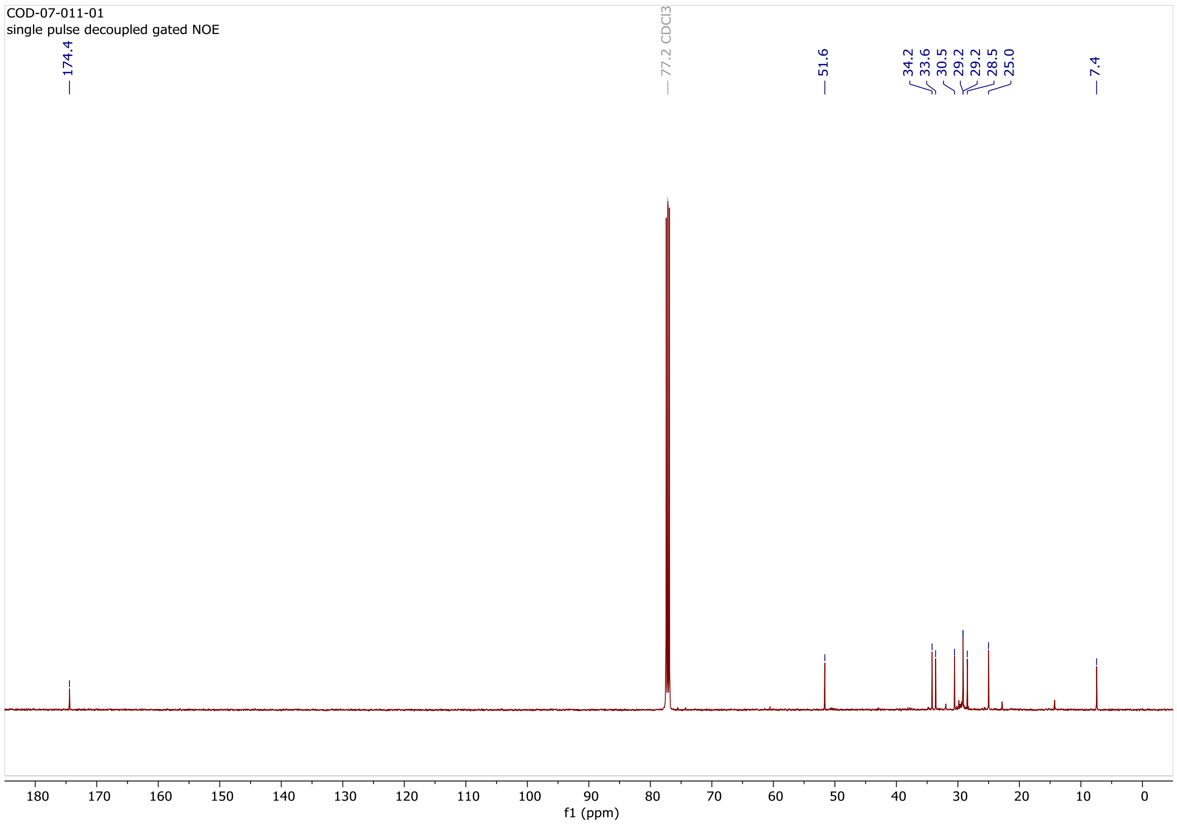

Copy of ^1H and $^{13}C\{^1H\}$ spectra of **1b**

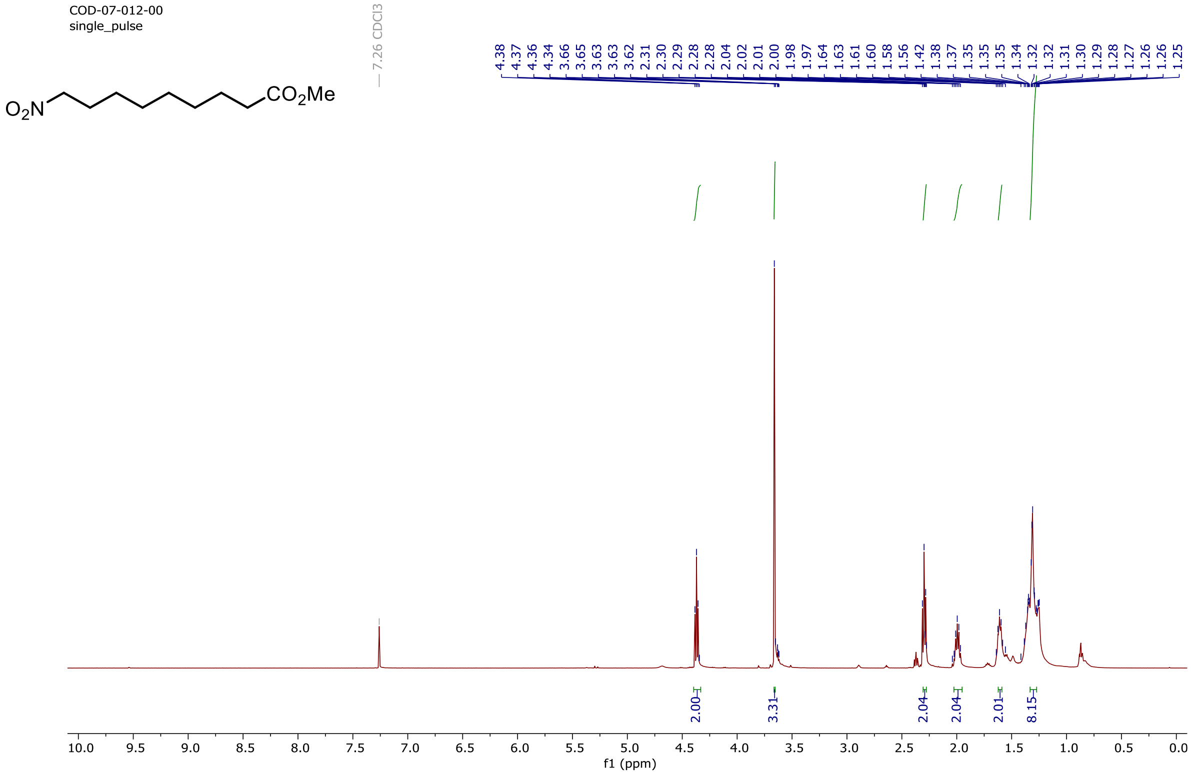

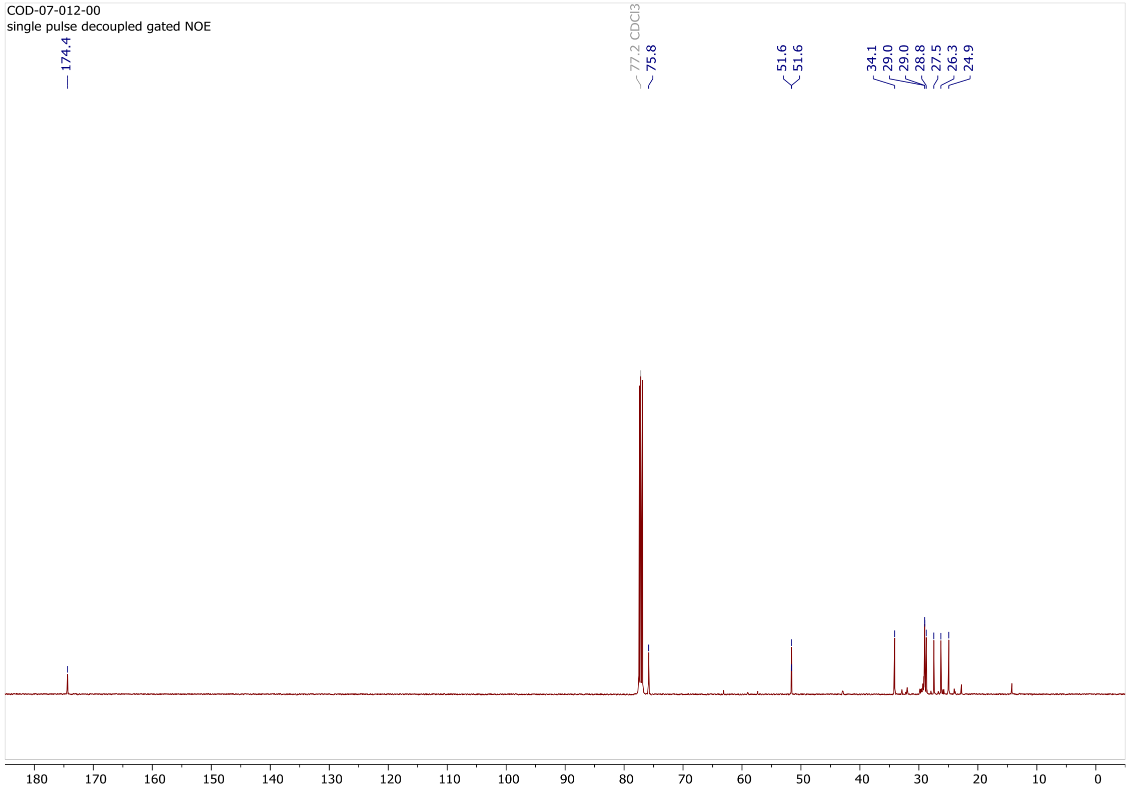

Copy of ^{1}H and $^{13}C\{^{1}H\}$ spectra of **3b**

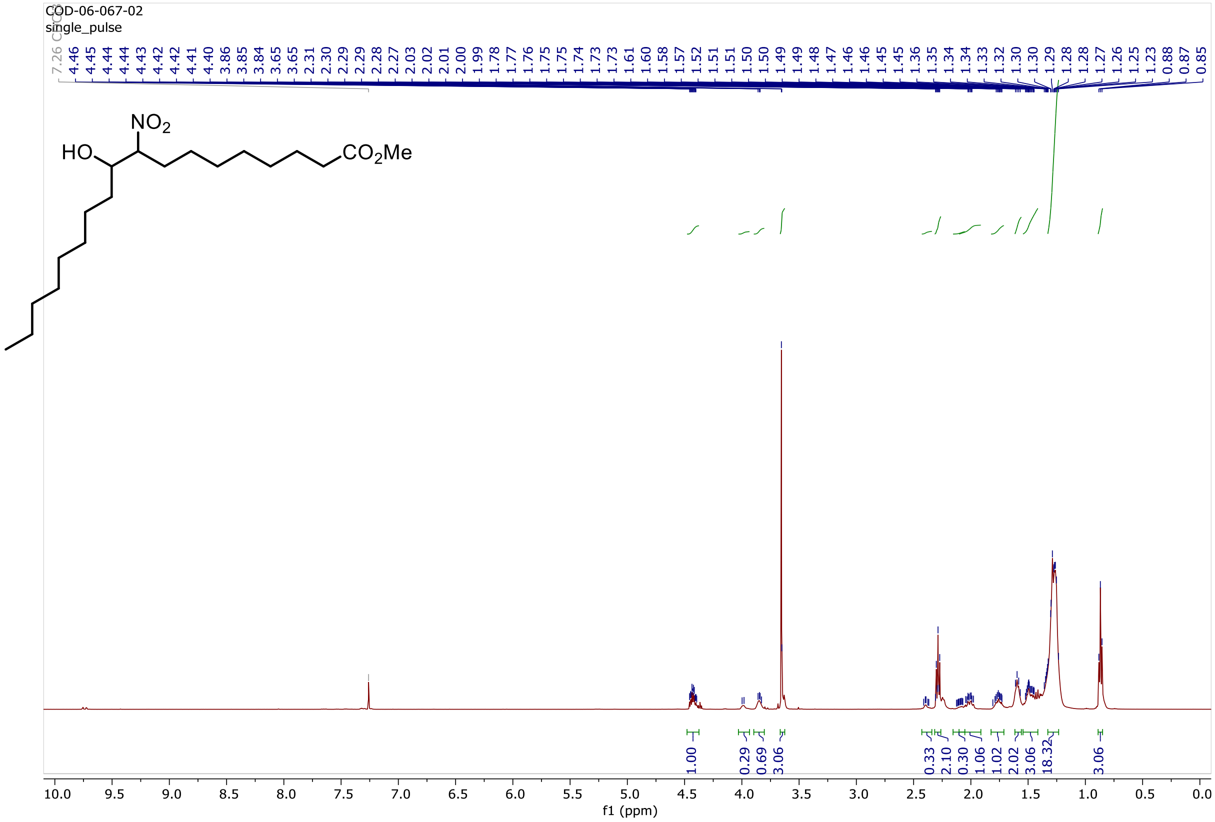

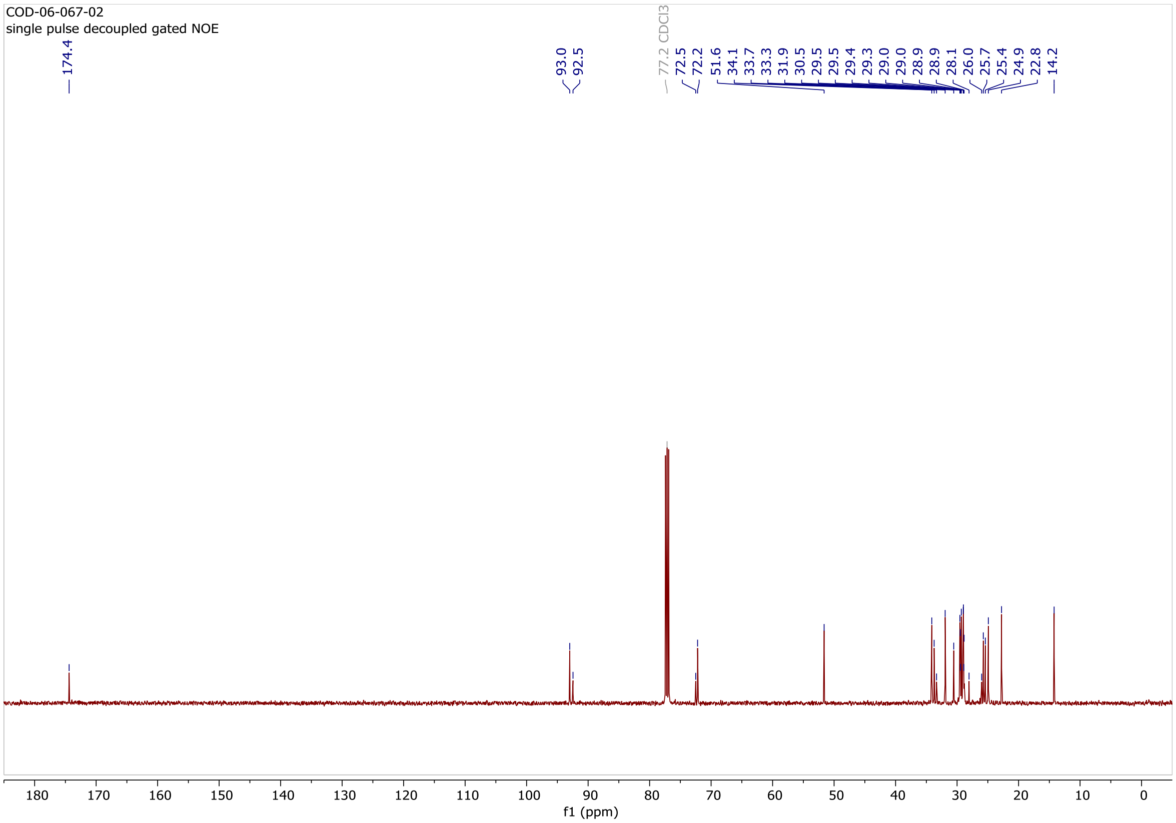

Copy of ^{1}H and $^{13}C\{^{1}H\}$ spectra of (9*E*)-**9-NO_2MeOA**

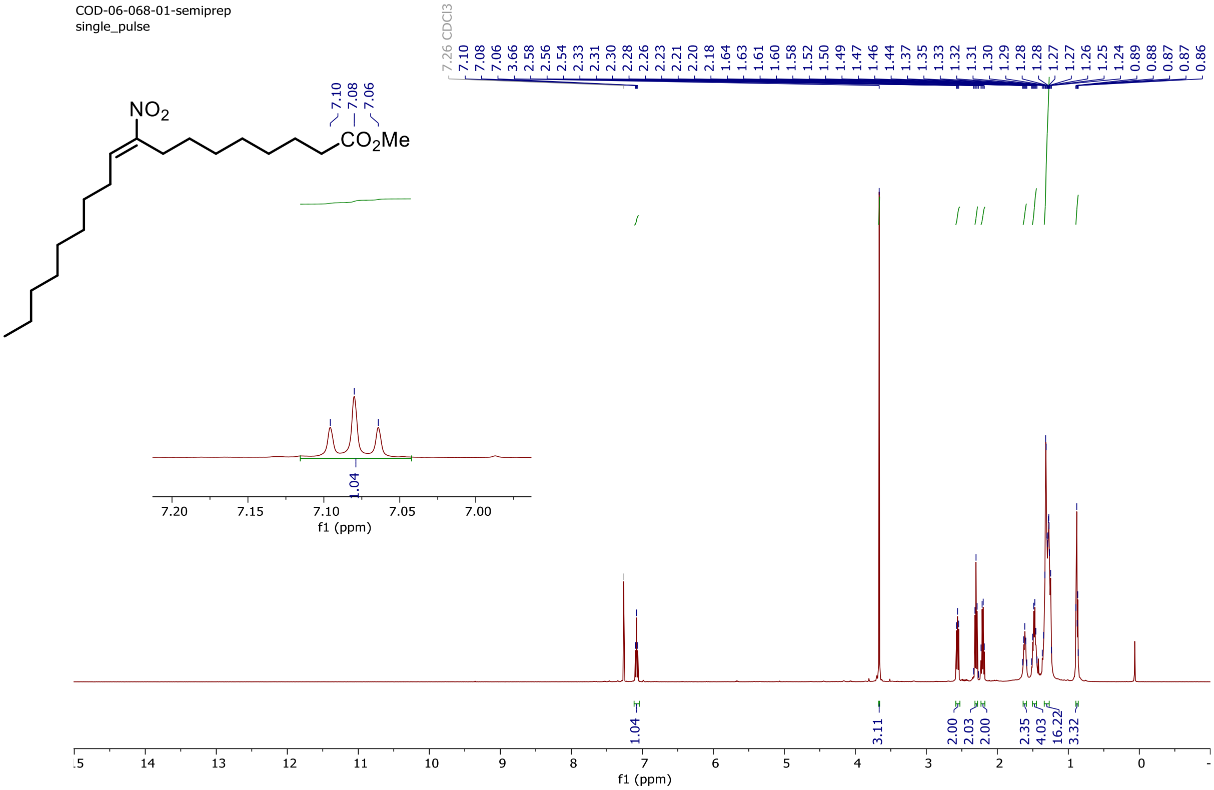

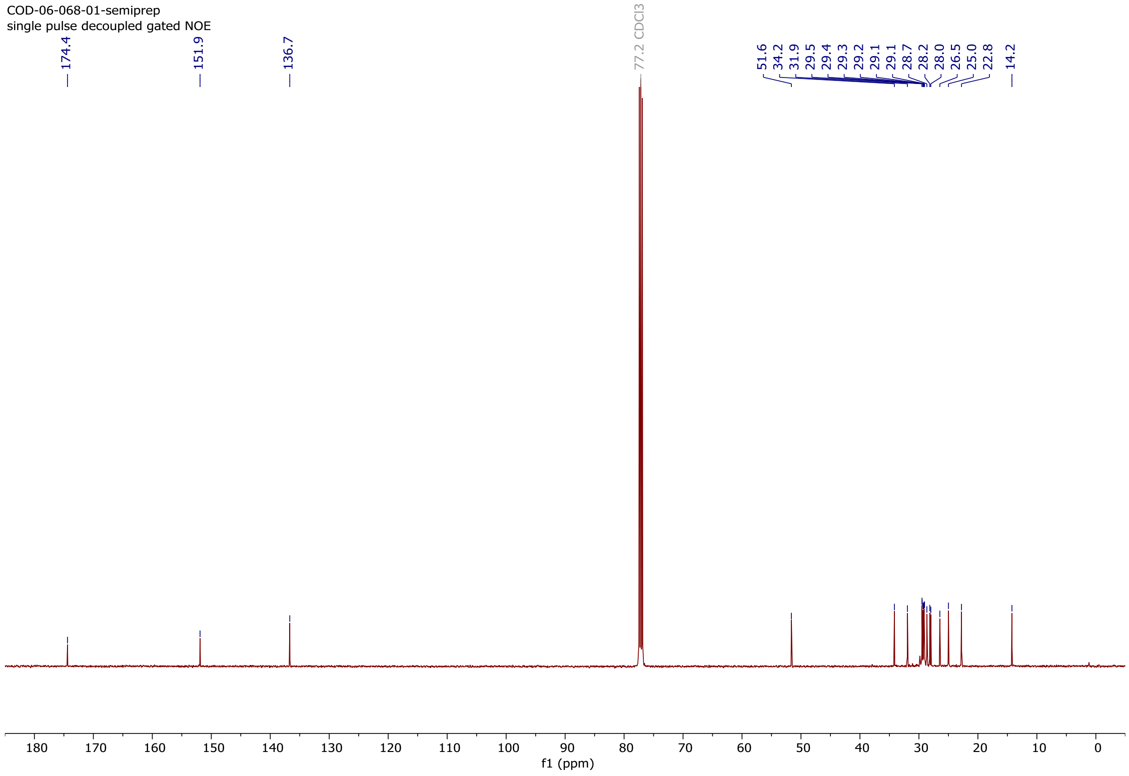

Copy of ^{1}H and $^{13}C\{^{1}H\}$ spectra of **9-NO_2OA**

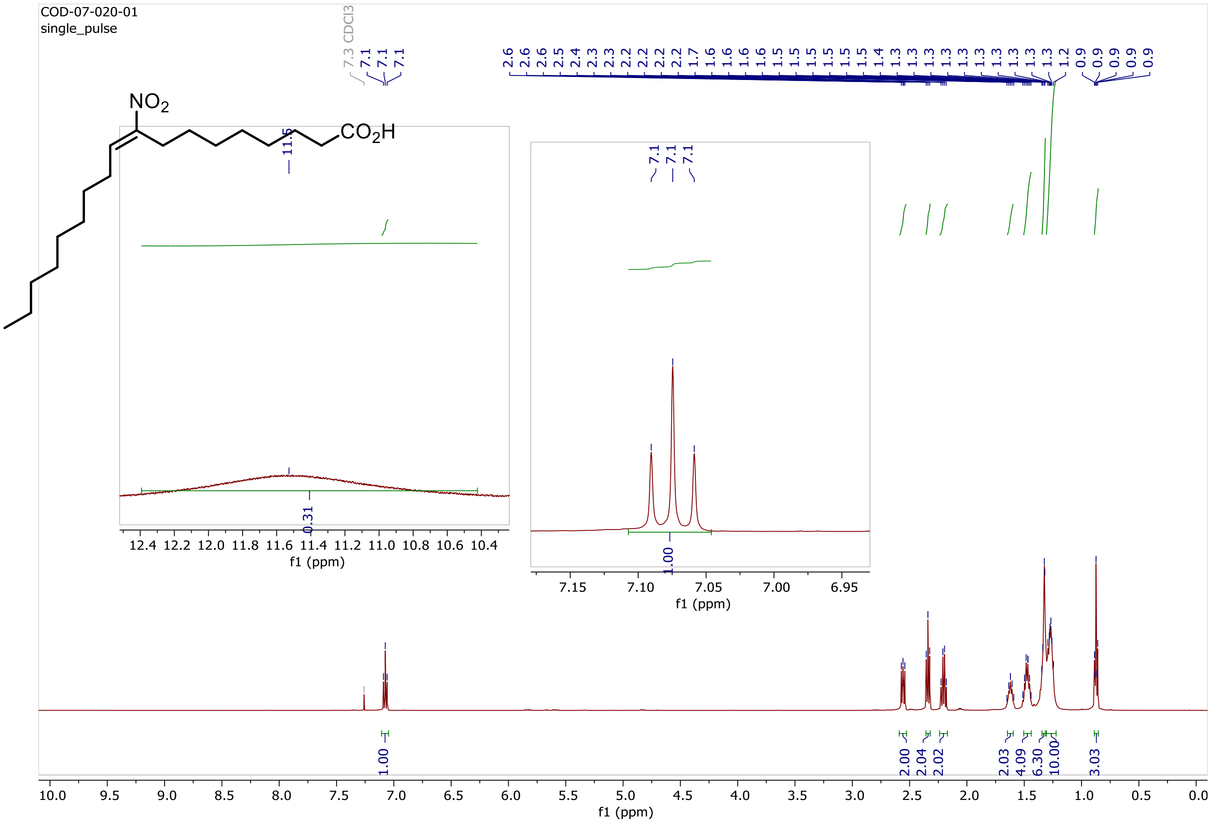

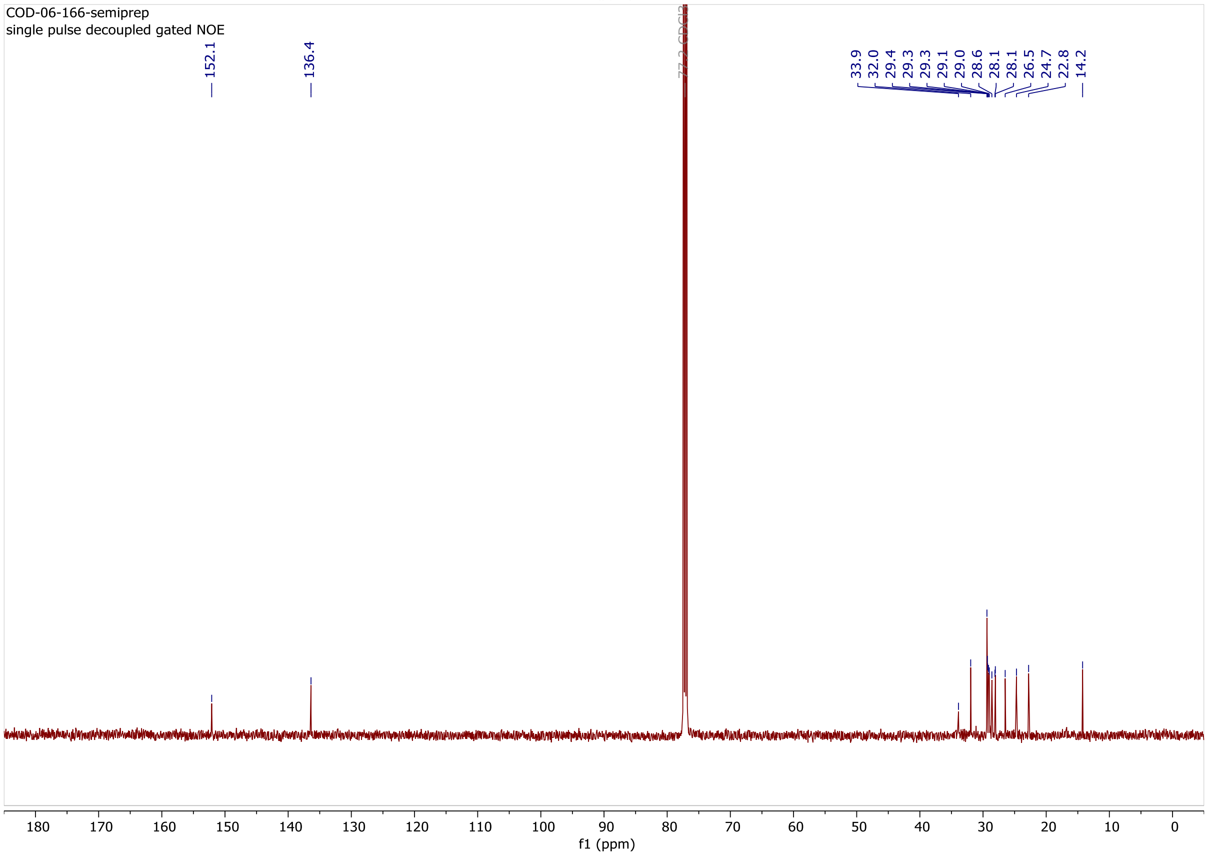

Copy of ^1H and $^{13}C\{^1H\}$ spectra of **S9**

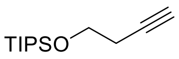

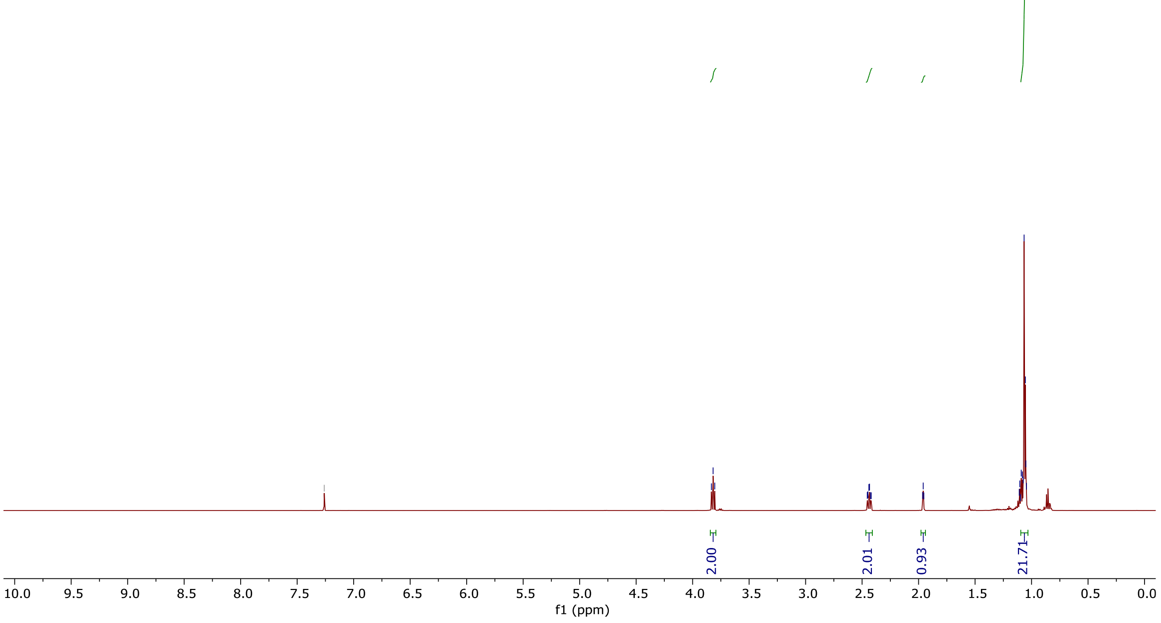

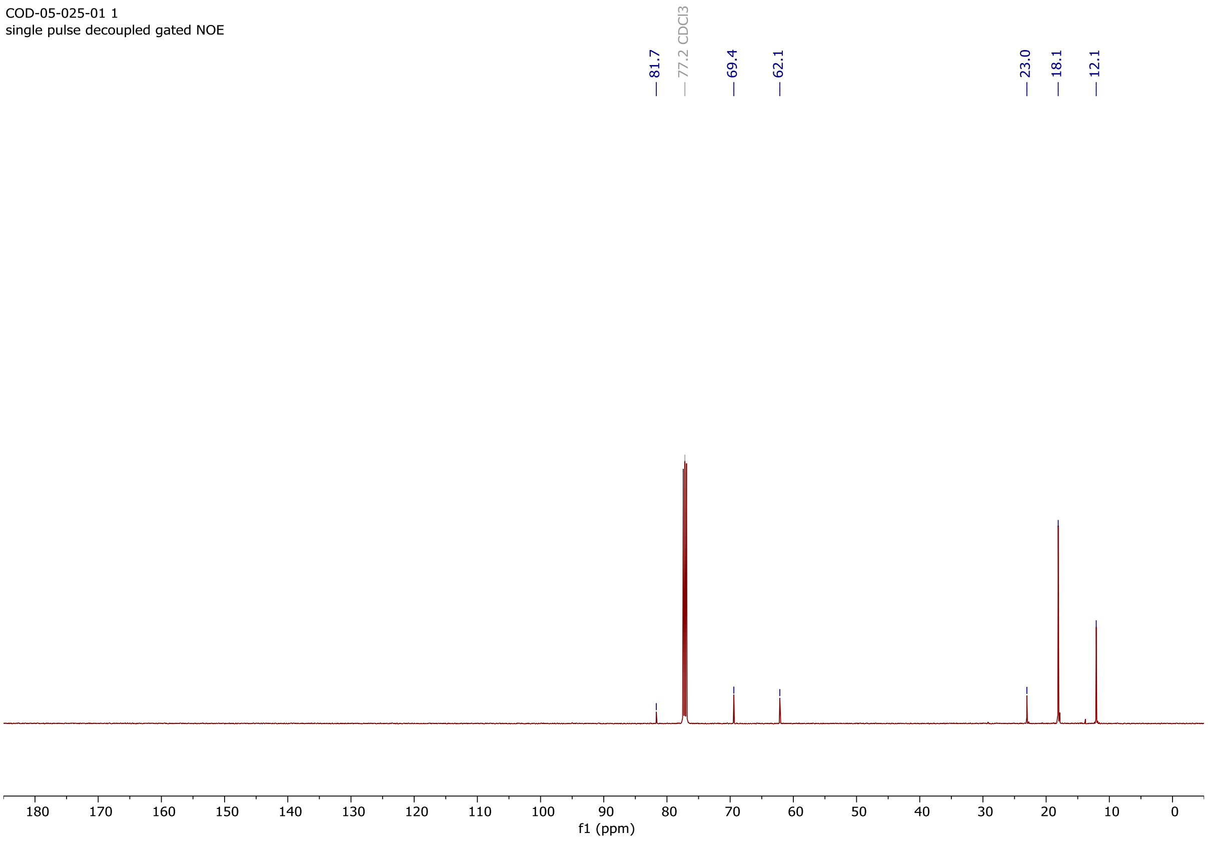

Copy of ^{1}H and $^{13}C\{^{1}H\}$ spectra of **S10**

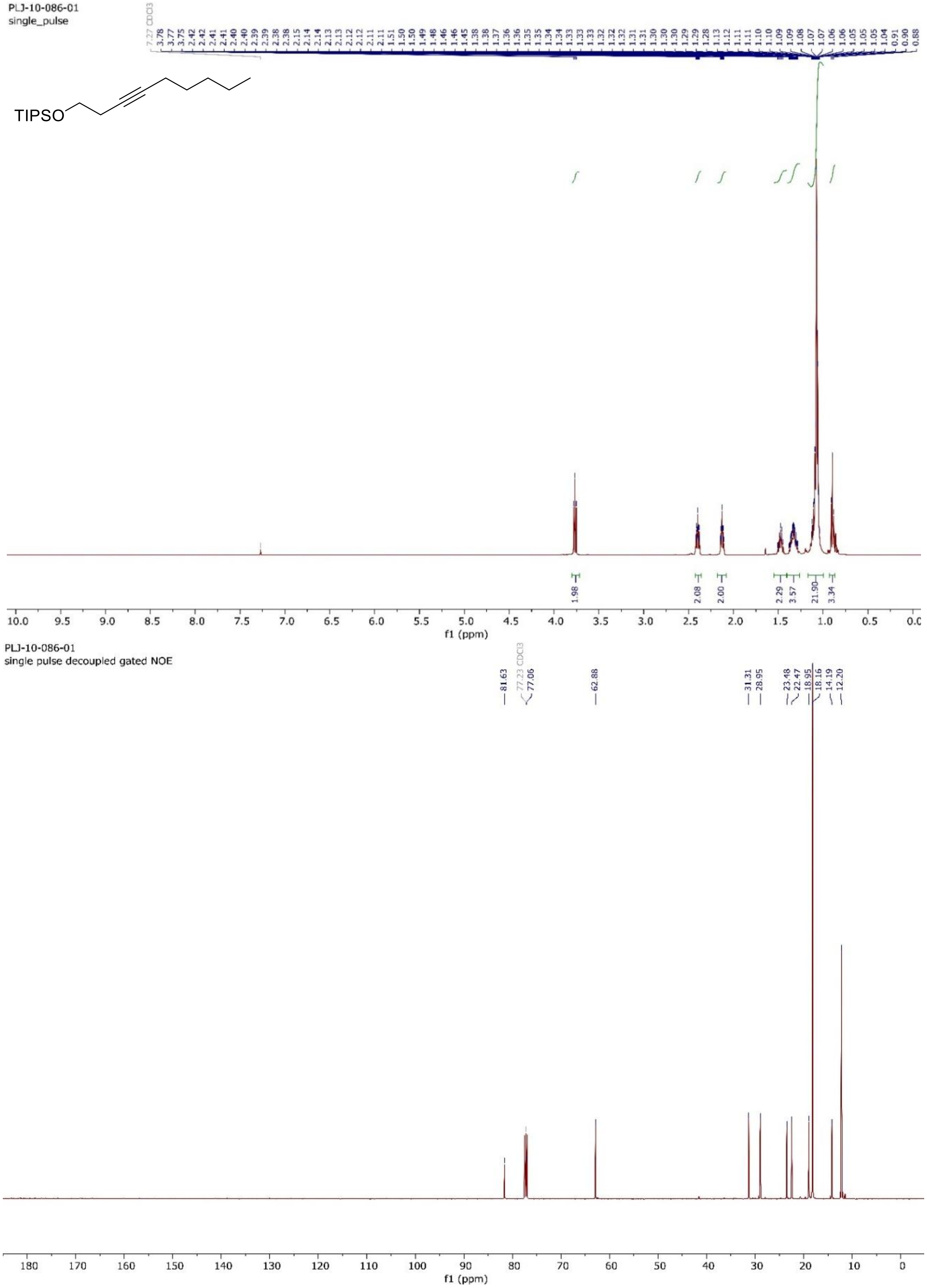

Copy of ^{1}H and $^{13}C\{^{1}H\}$ spectra of **S11**

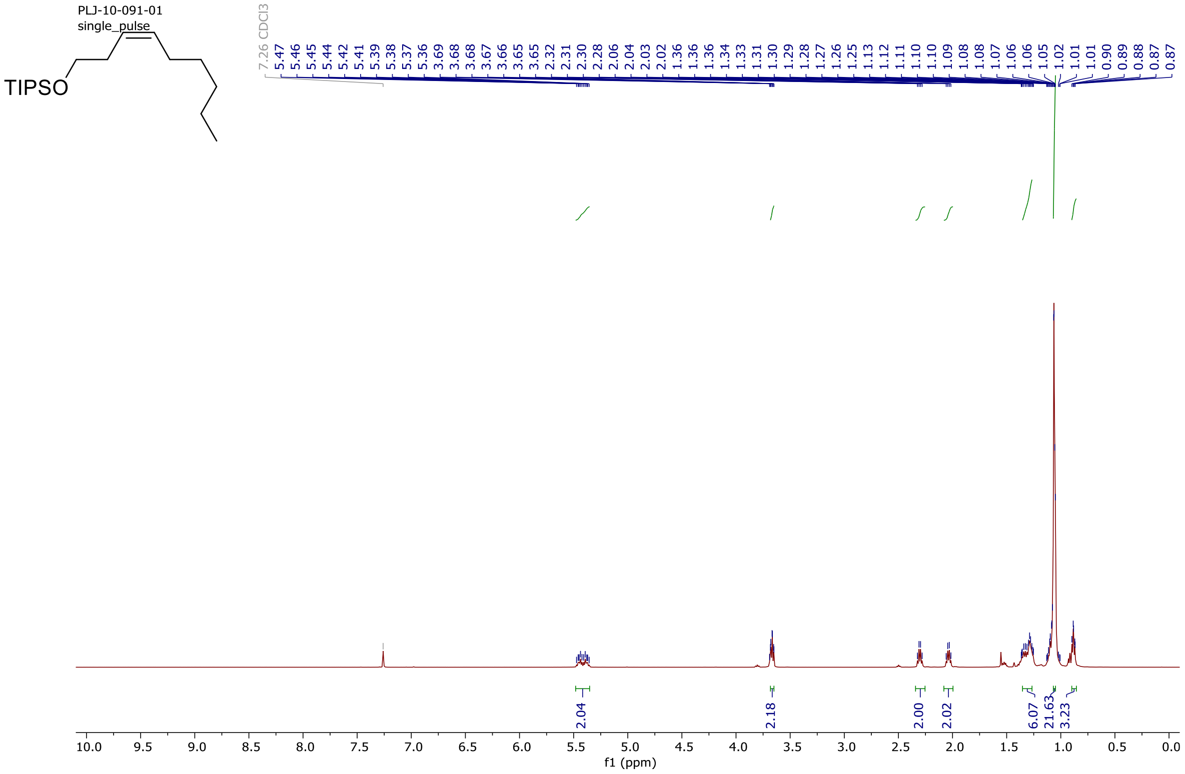

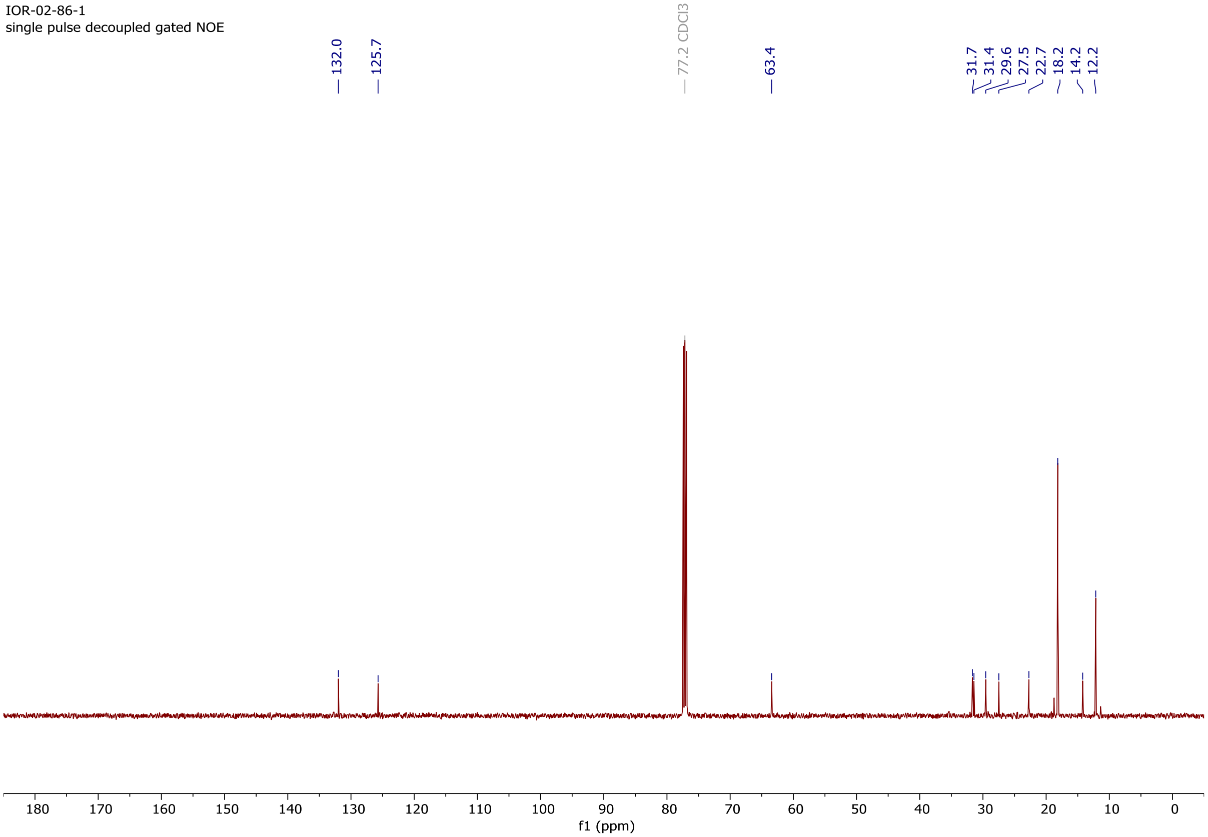

Copy of ^1H and $^{13}C\{^1H\}$ spectra of **4**

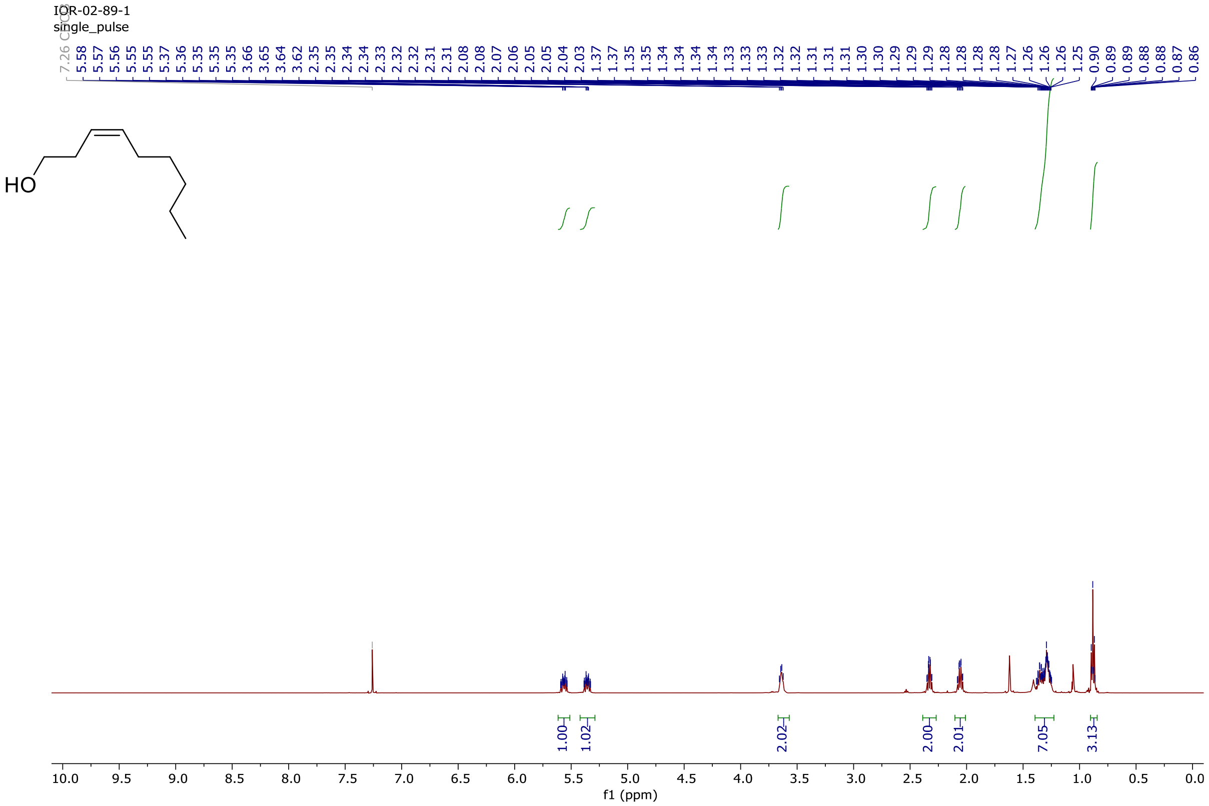

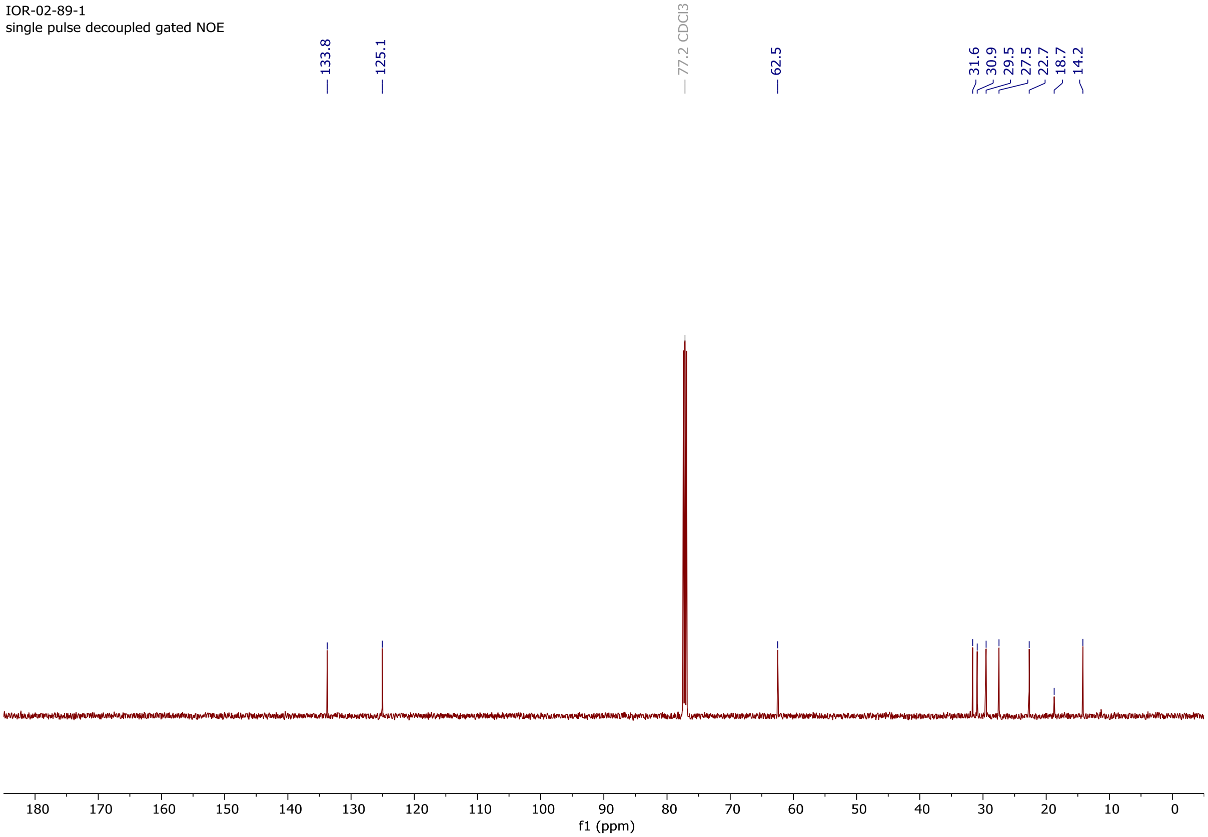

Copy of ^{1}H and ^{13}C{^{1}H} spectra of **S12**

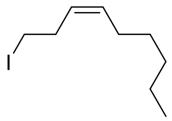

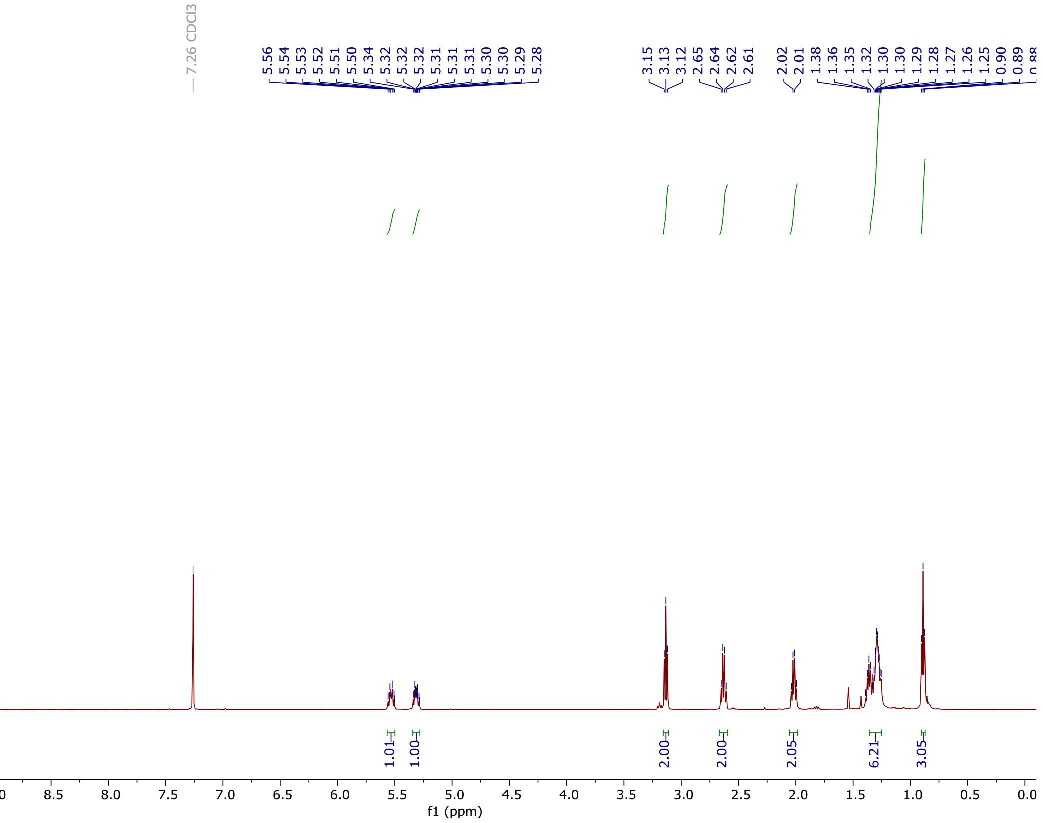

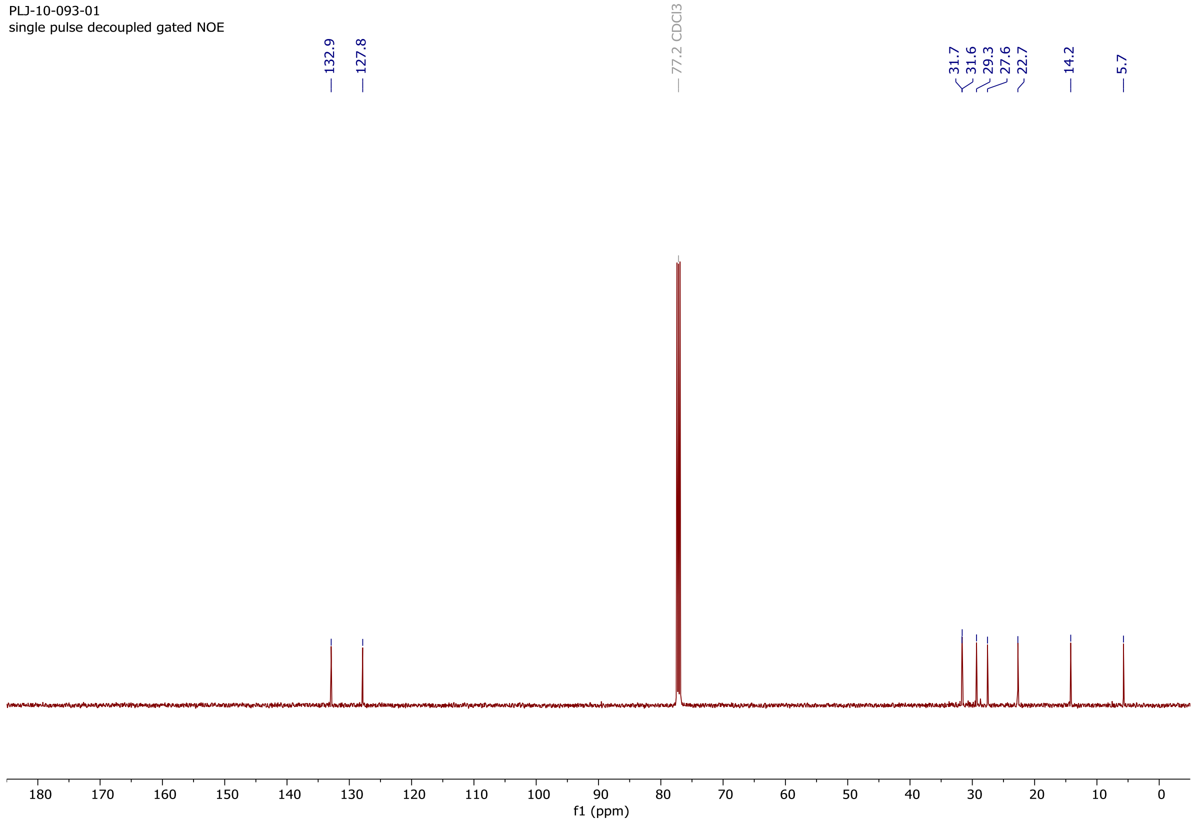

Copy of ^{1}H and $^{13}C\{^{1}H\}$ spectra of **1c**

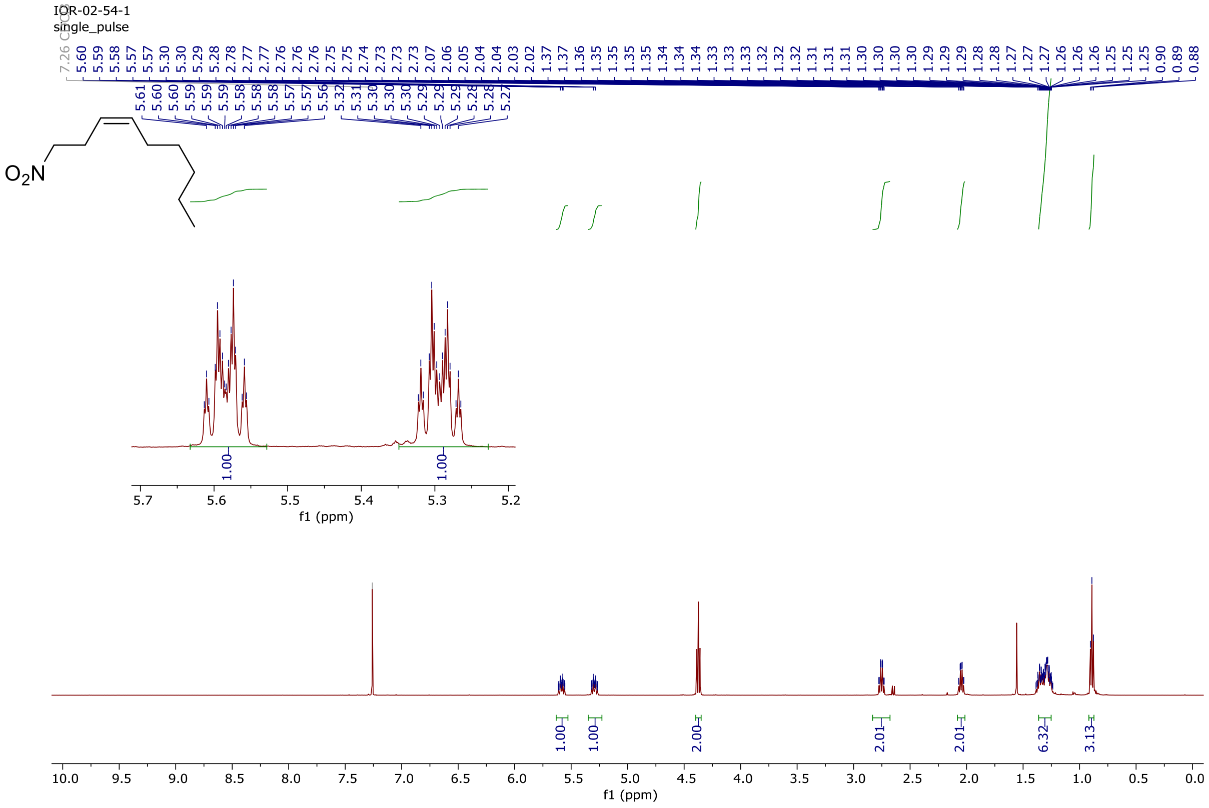

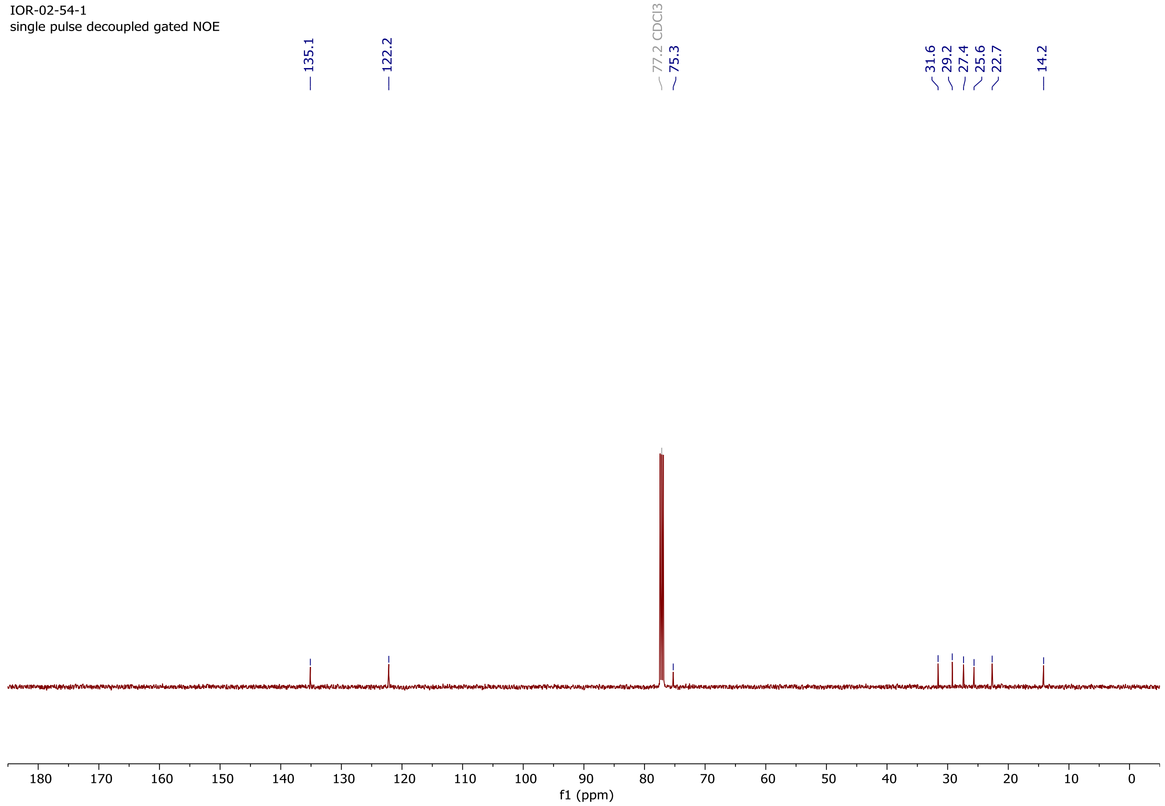

Copy of ^{1}H and ^{13}C{^{1}H} spectra of **2a**

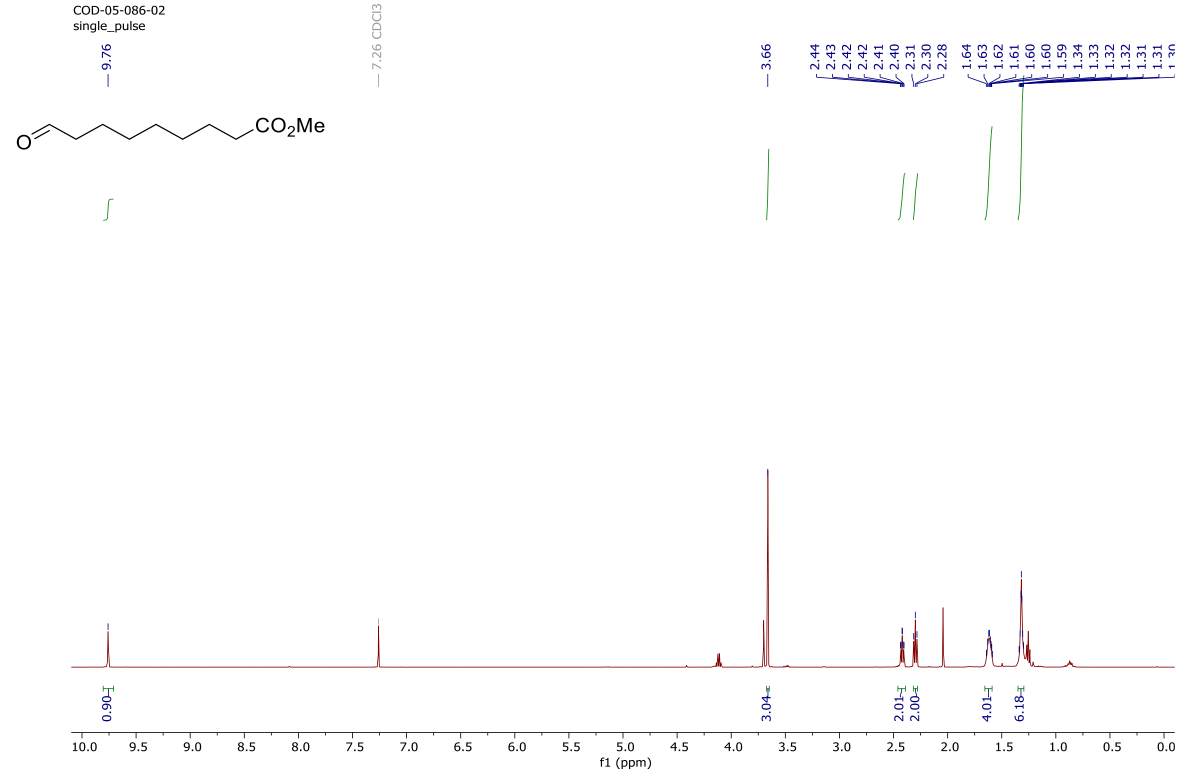

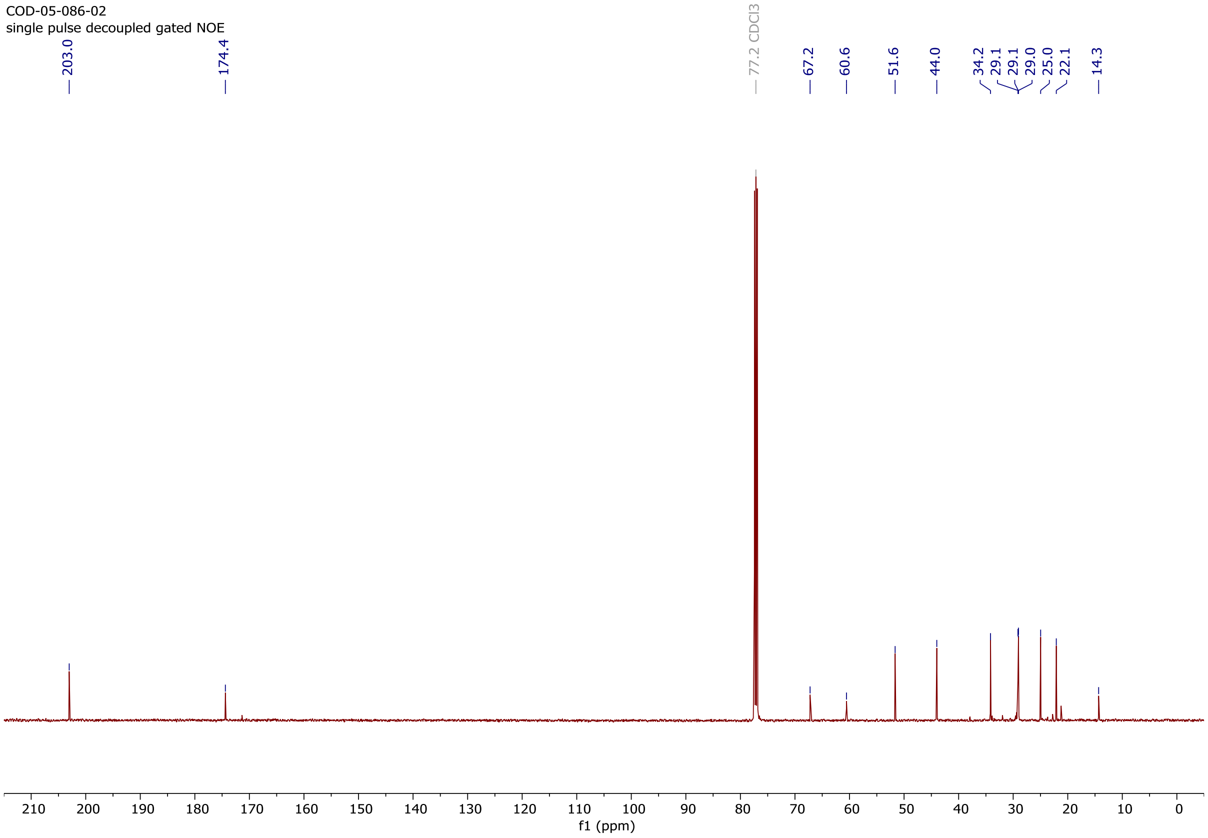

Copy of ^{1}H and $^{13}C\{^{1}H\}$ spectra of **3c**

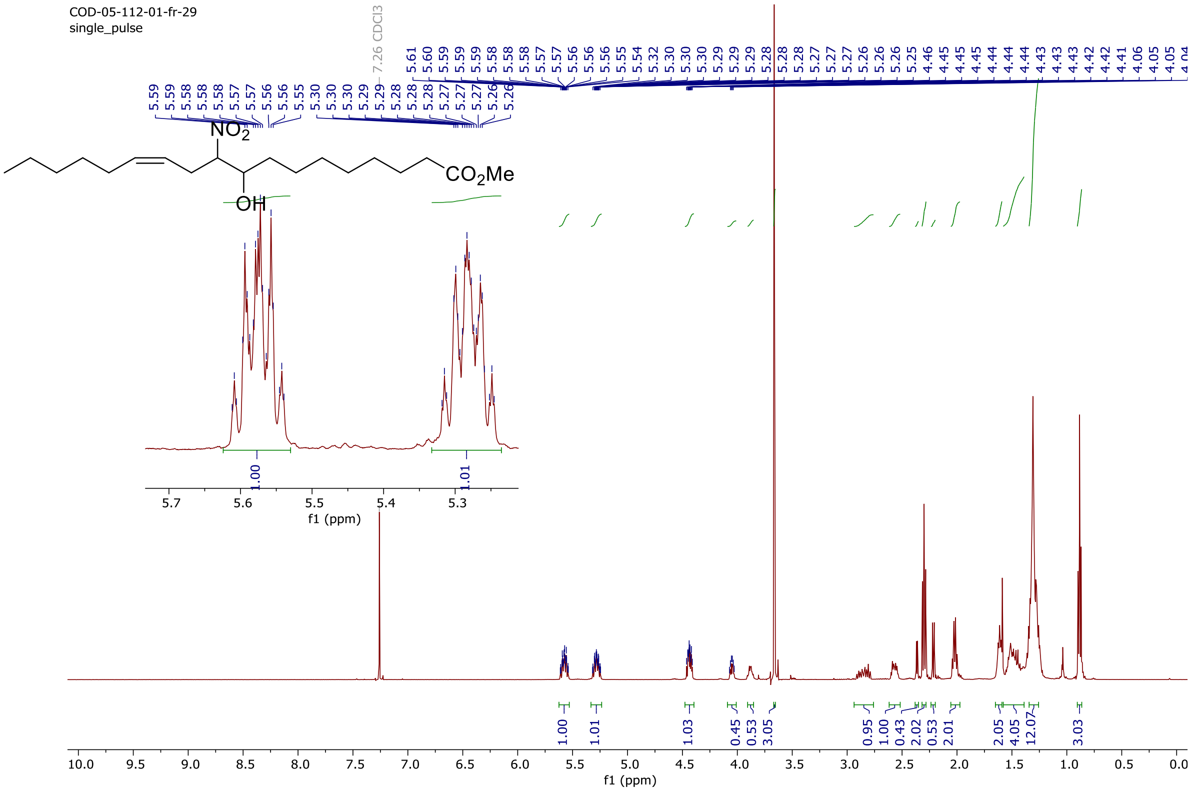

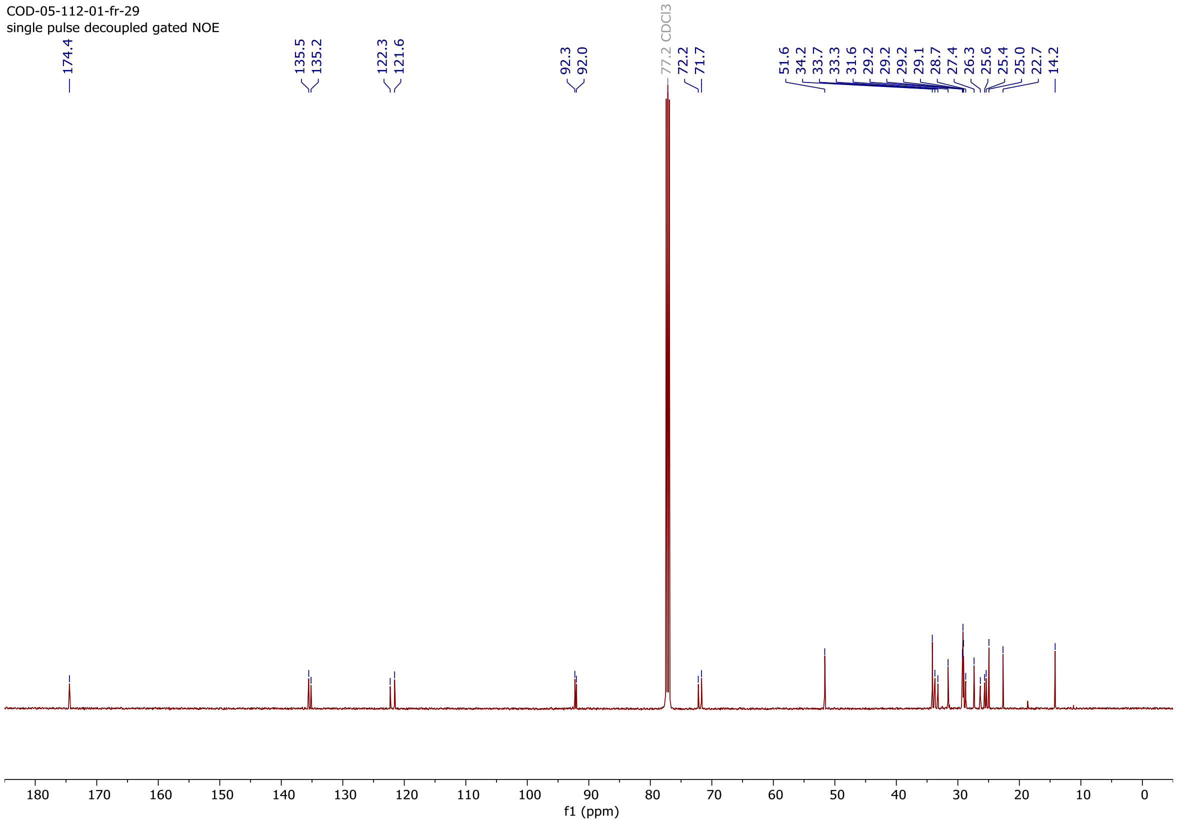

Copy of ^{1}H and $^{13}C\{^{1}H\}$ spectra of **10-NO₂MeLA**

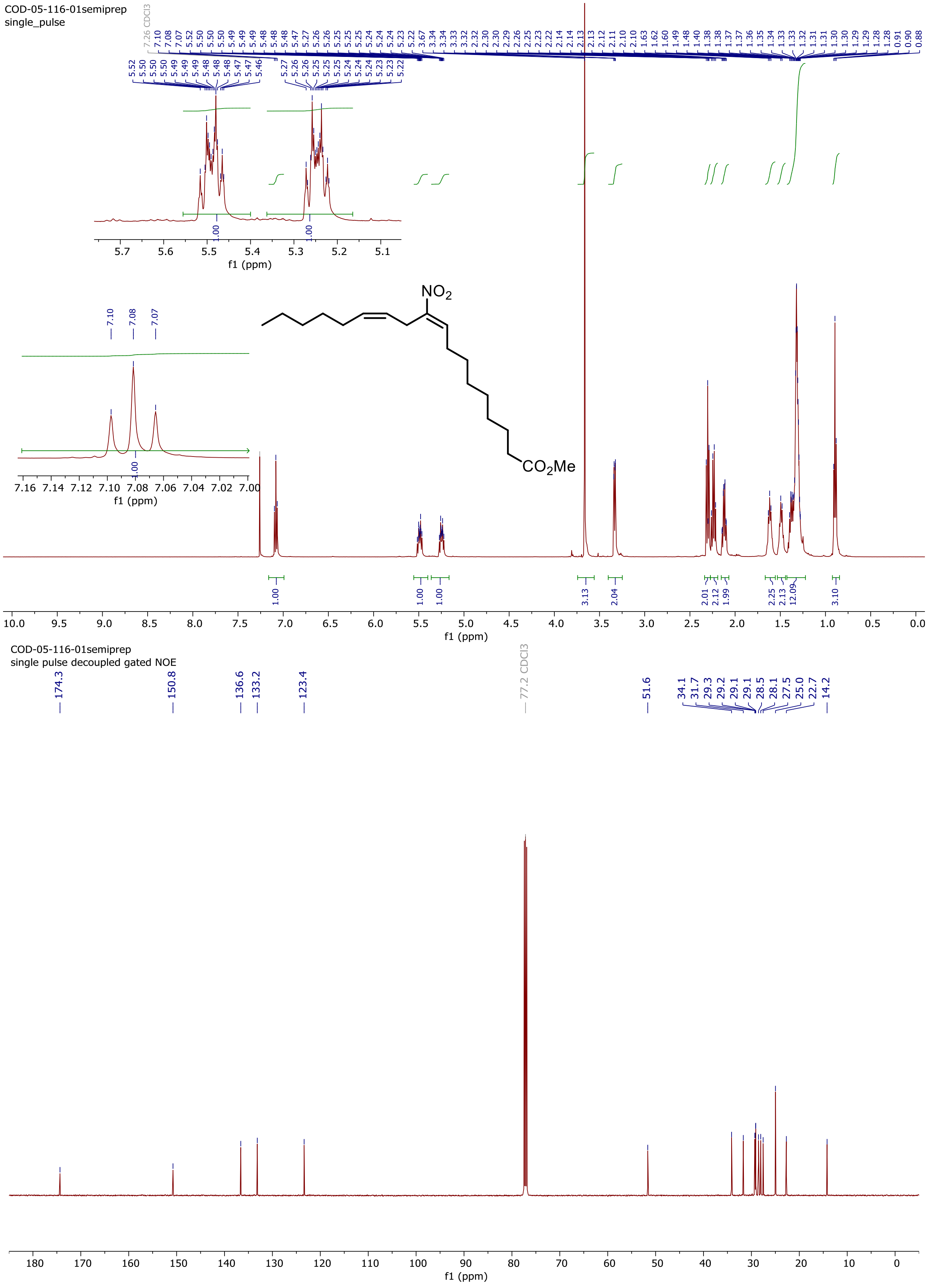

Copy of ^{1}H and $^{13}C\{^{1}H\}$ spectra of **10-NO_2LA**

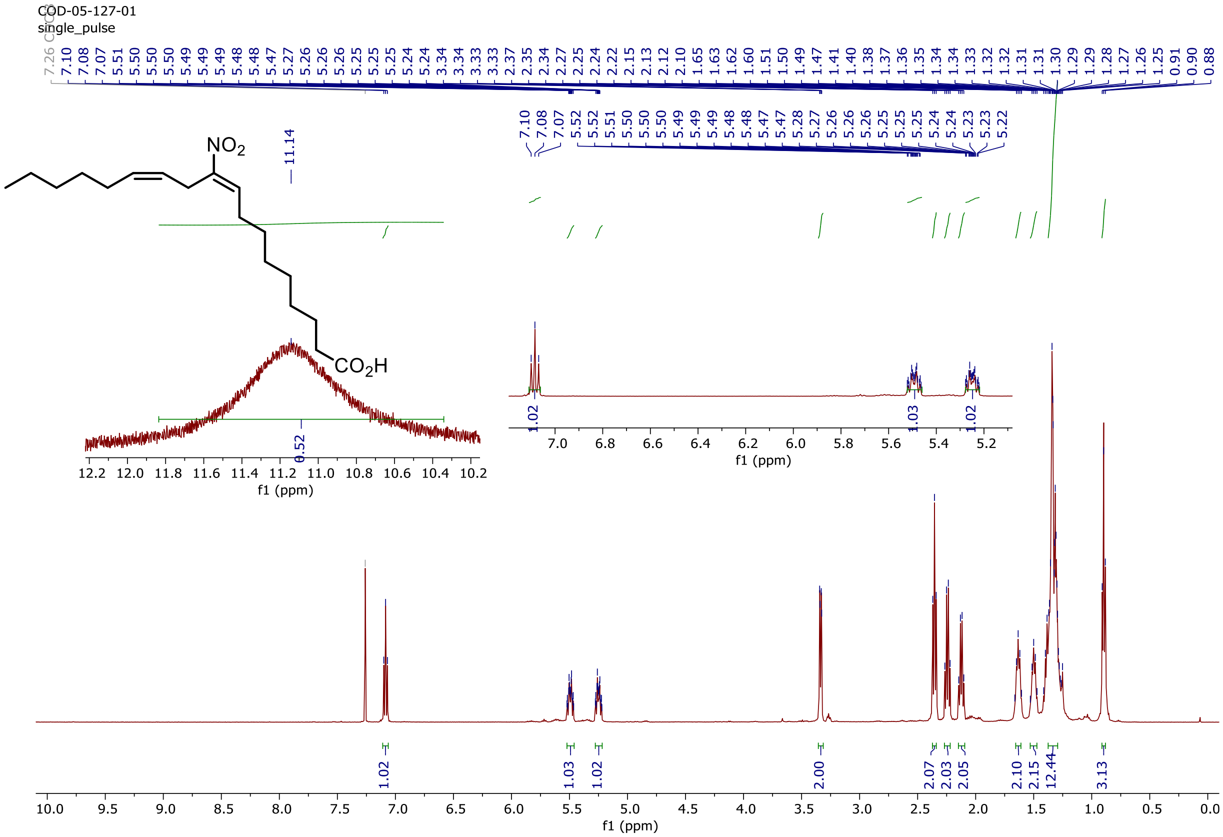

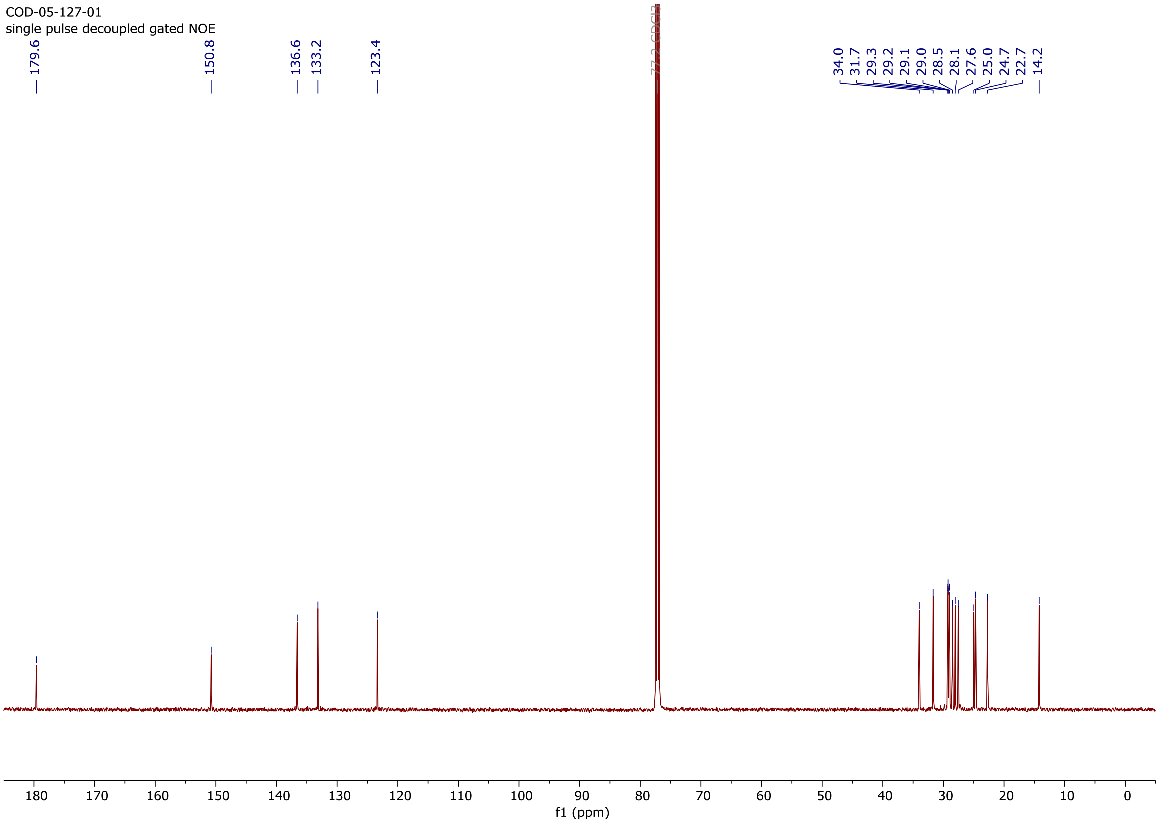

Copy of ^{1}H and $^{13}C\{^{1}H\}$ spectra of **5**

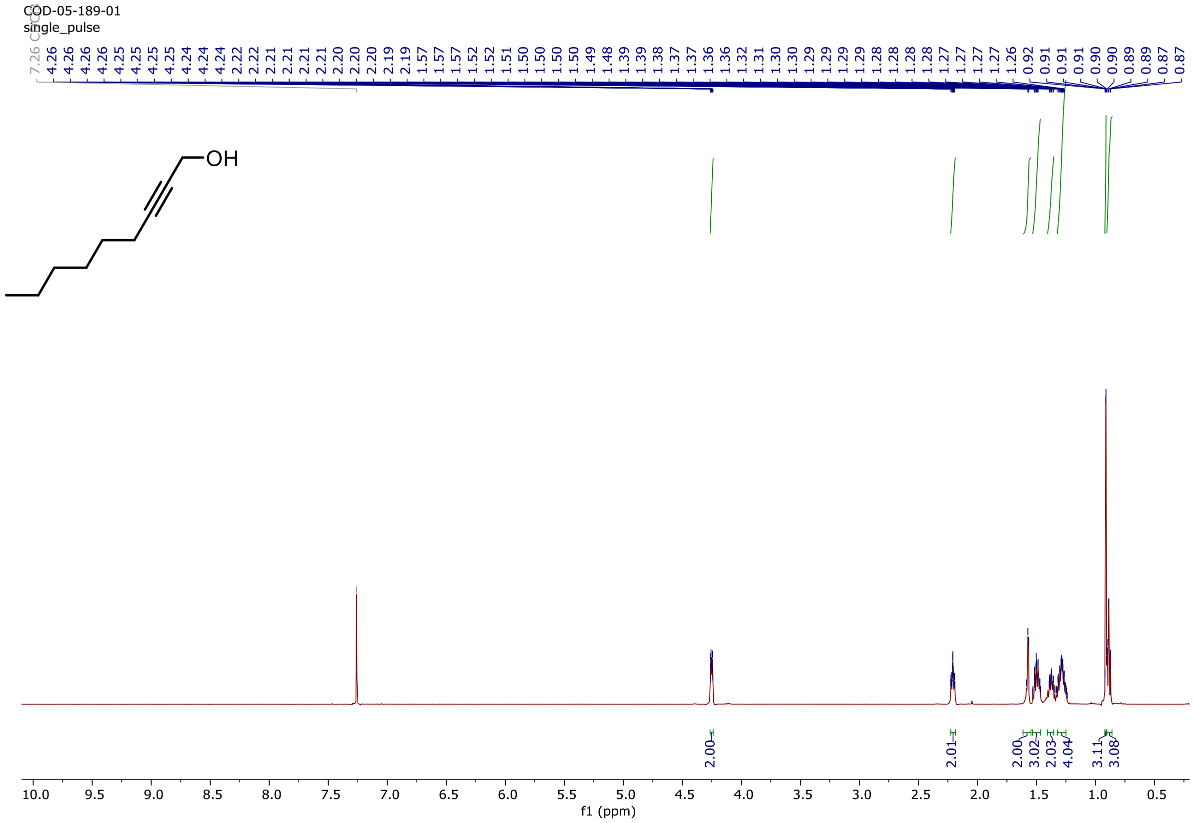

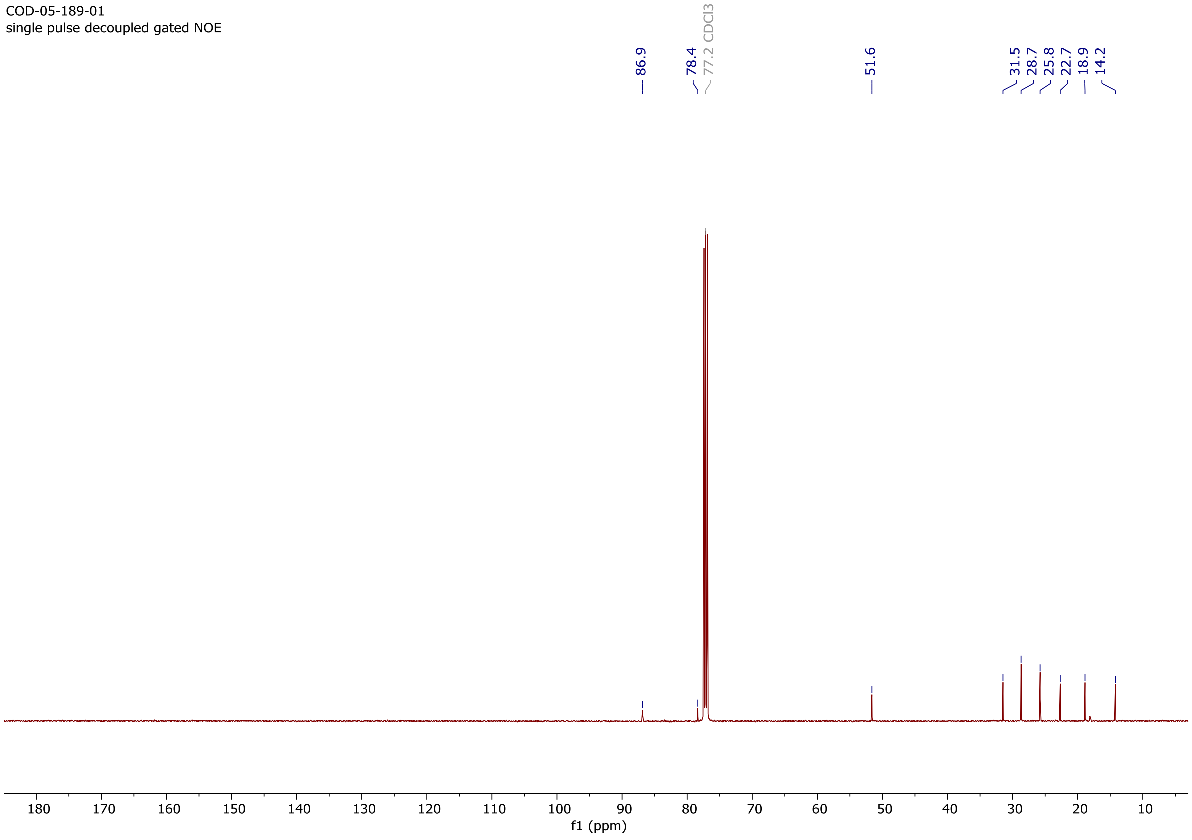

Copy of ^{1}H and ^{13}C{^{1}H} spectra of **S15**

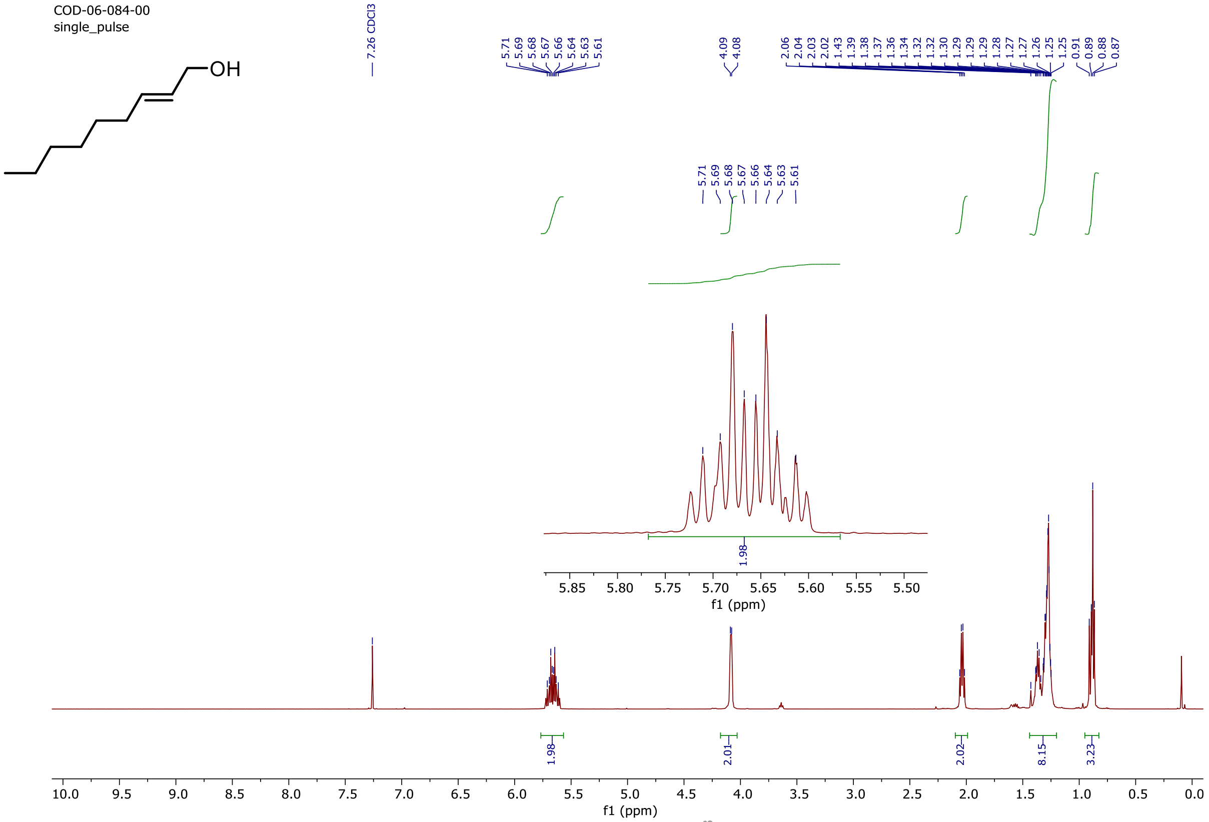

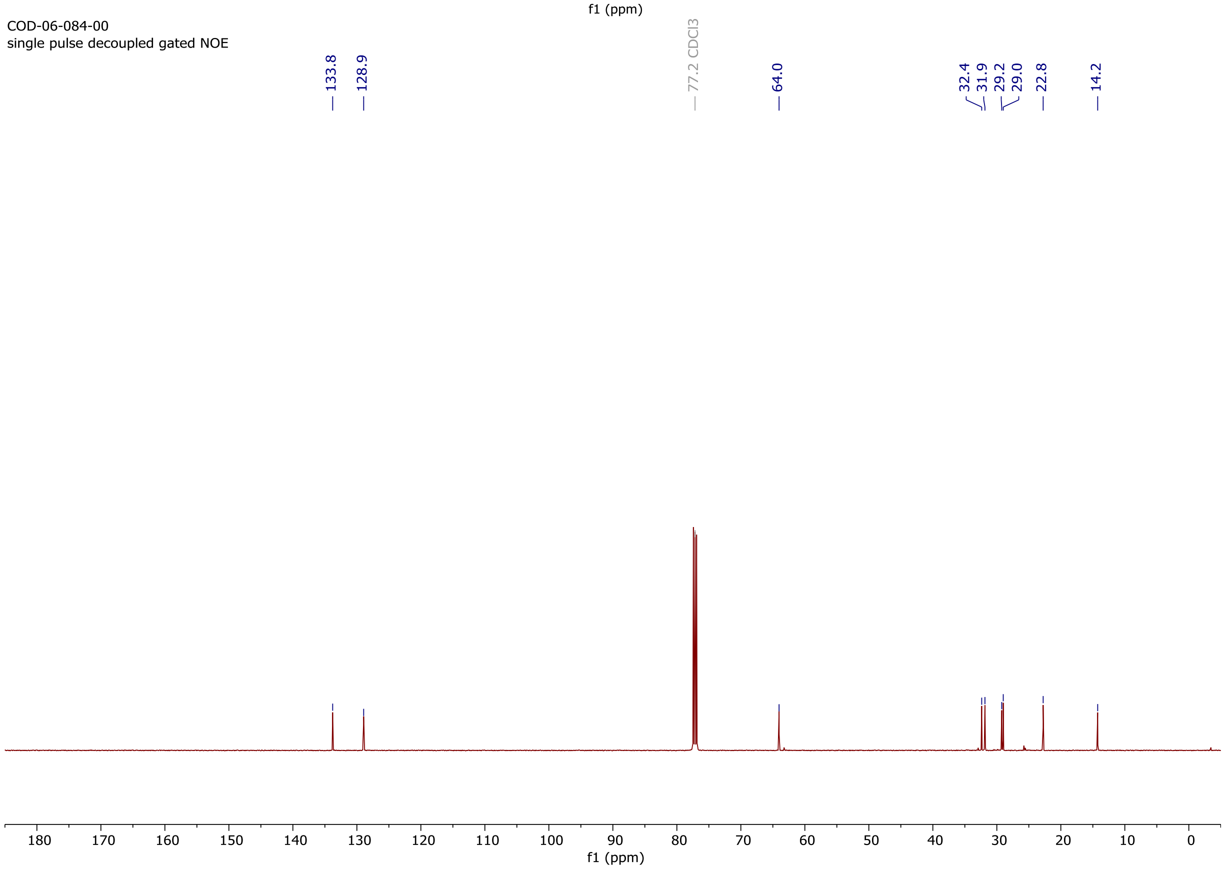

Copy of ^{1}H and $^{13}C\{^{1}H\}$ spectra of **2c**

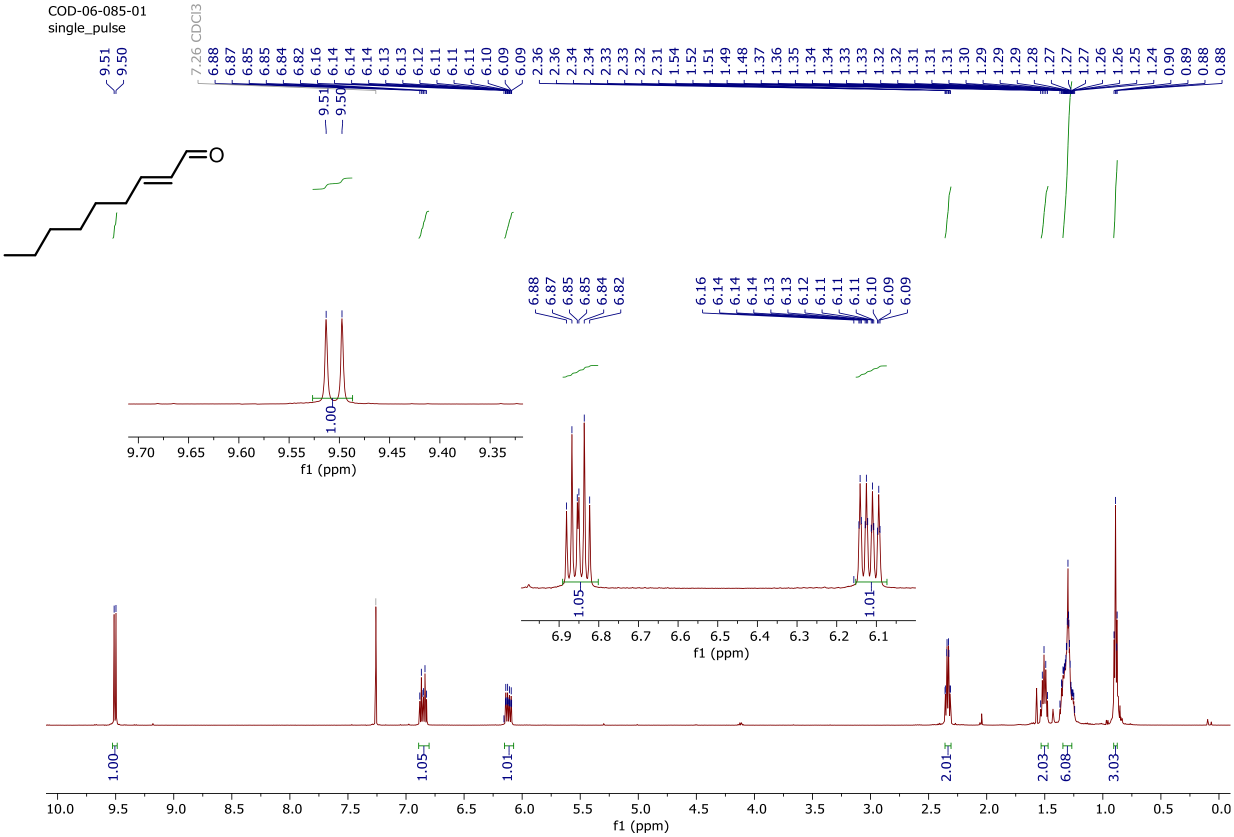

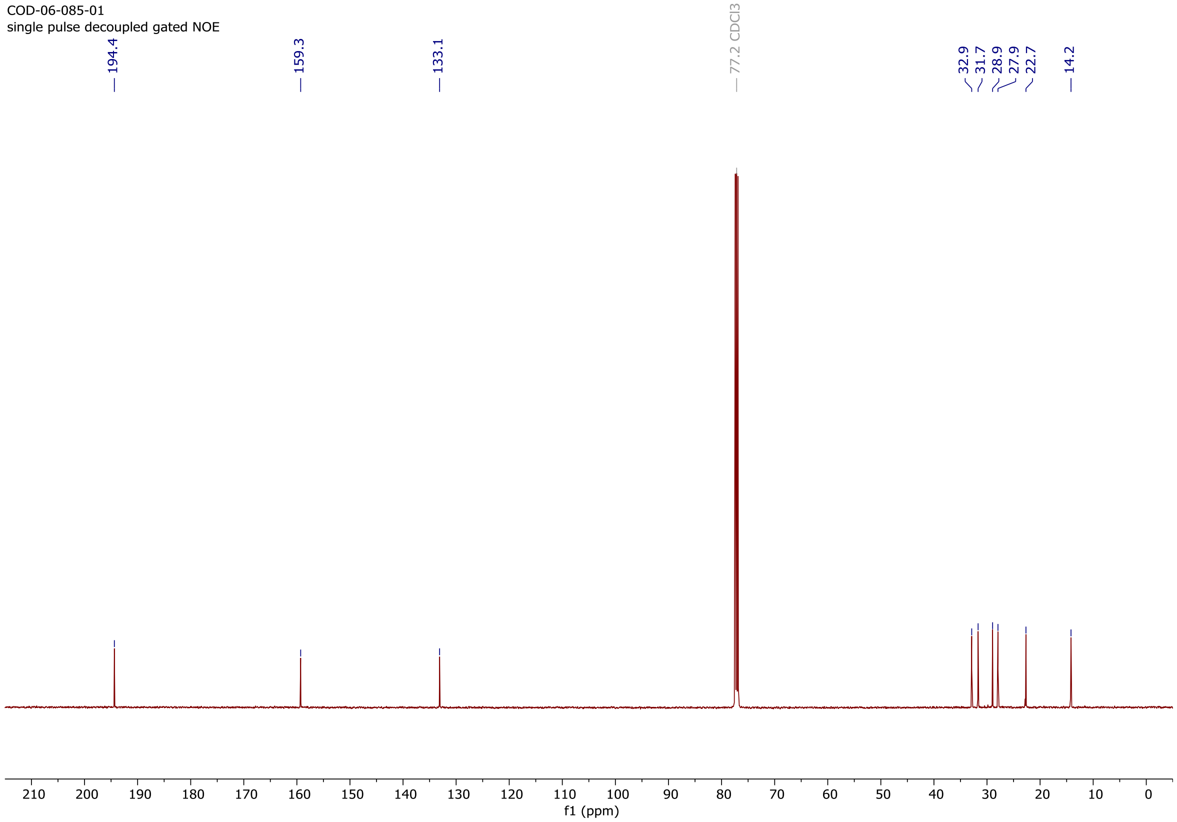

Copy of ^{1}H and ^{13}C{^{1}H} spectra of **9-NO₂MecLA**

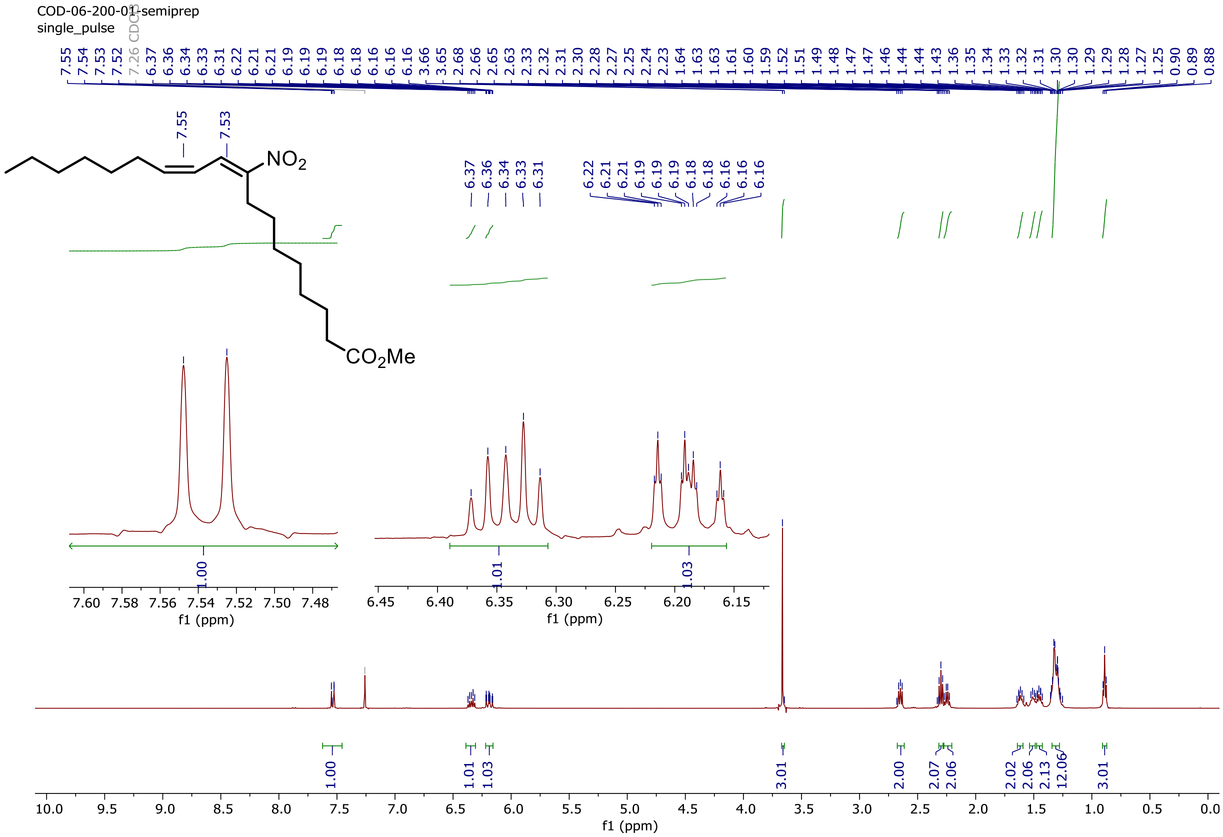

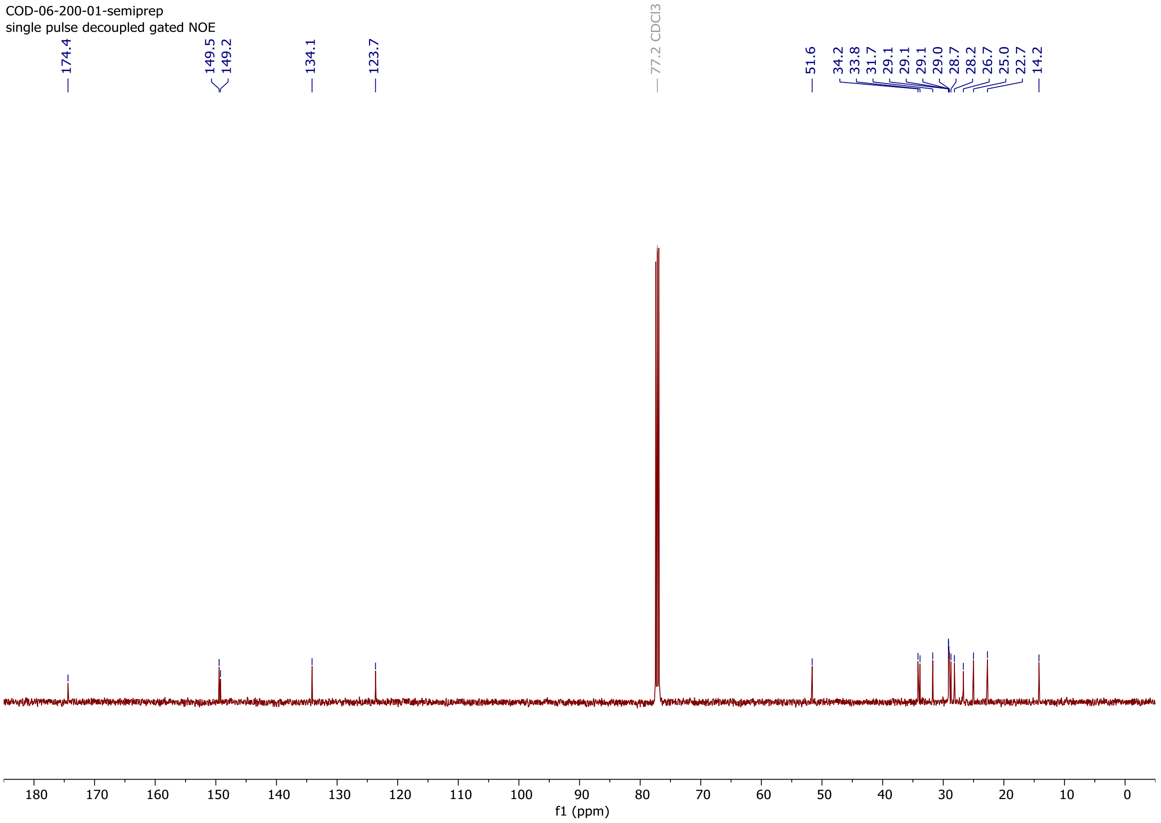

Copy of ^{1}H and $^{13}C\{^{1}H\}$ spectra of **9-NO₂cLA**

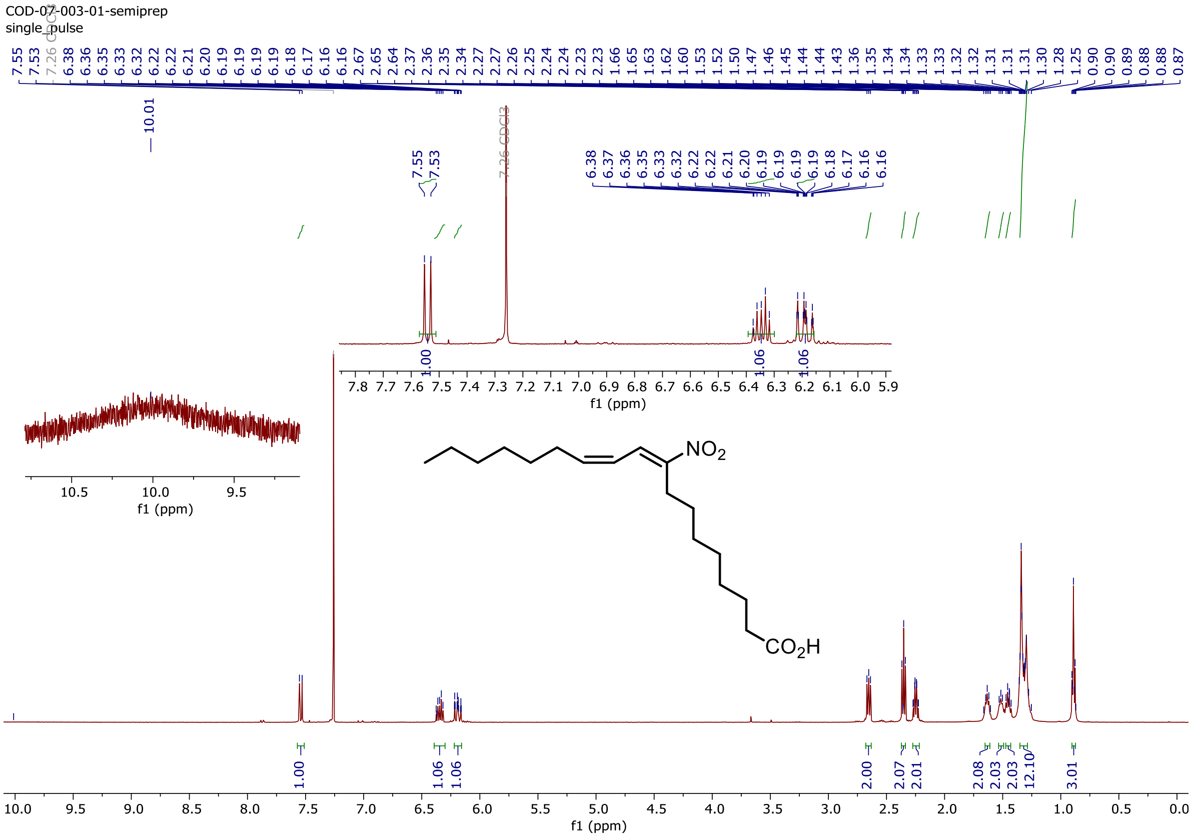

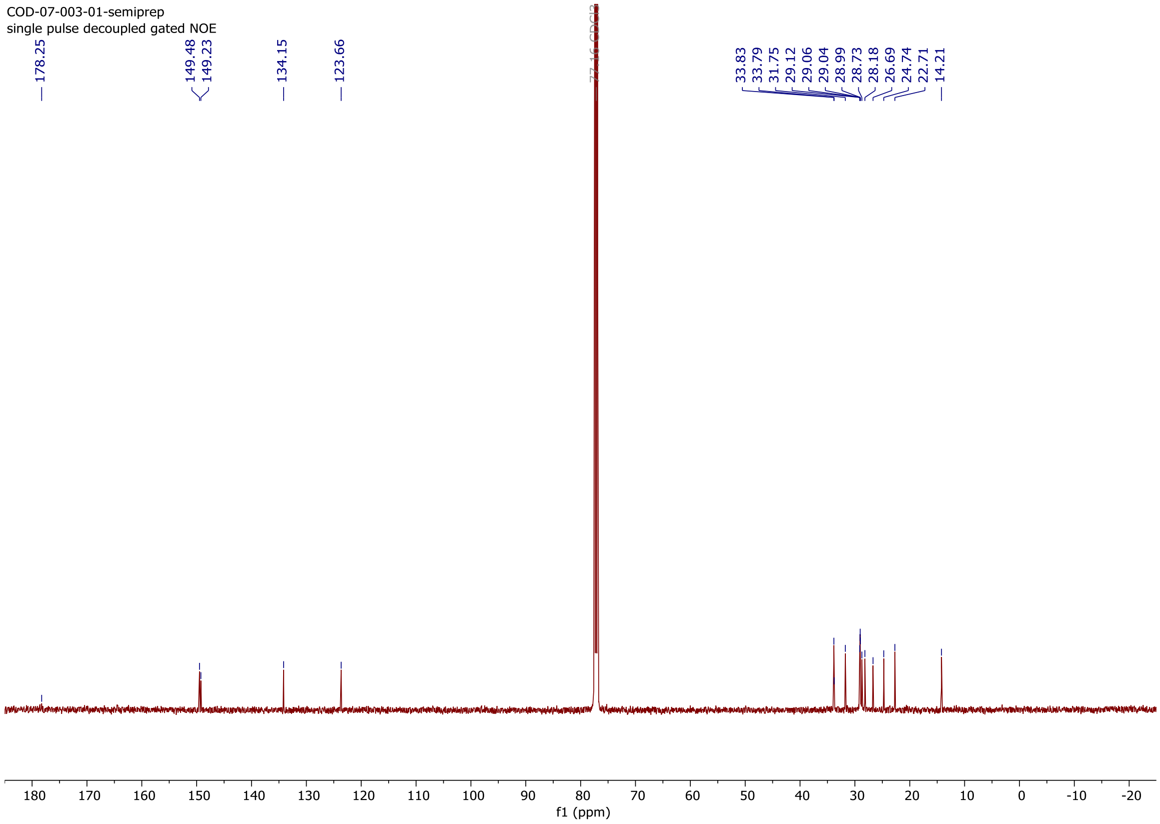

Copy of ^{1}H and $^{13}C\{^{1}H\}$ spectra of **S16**

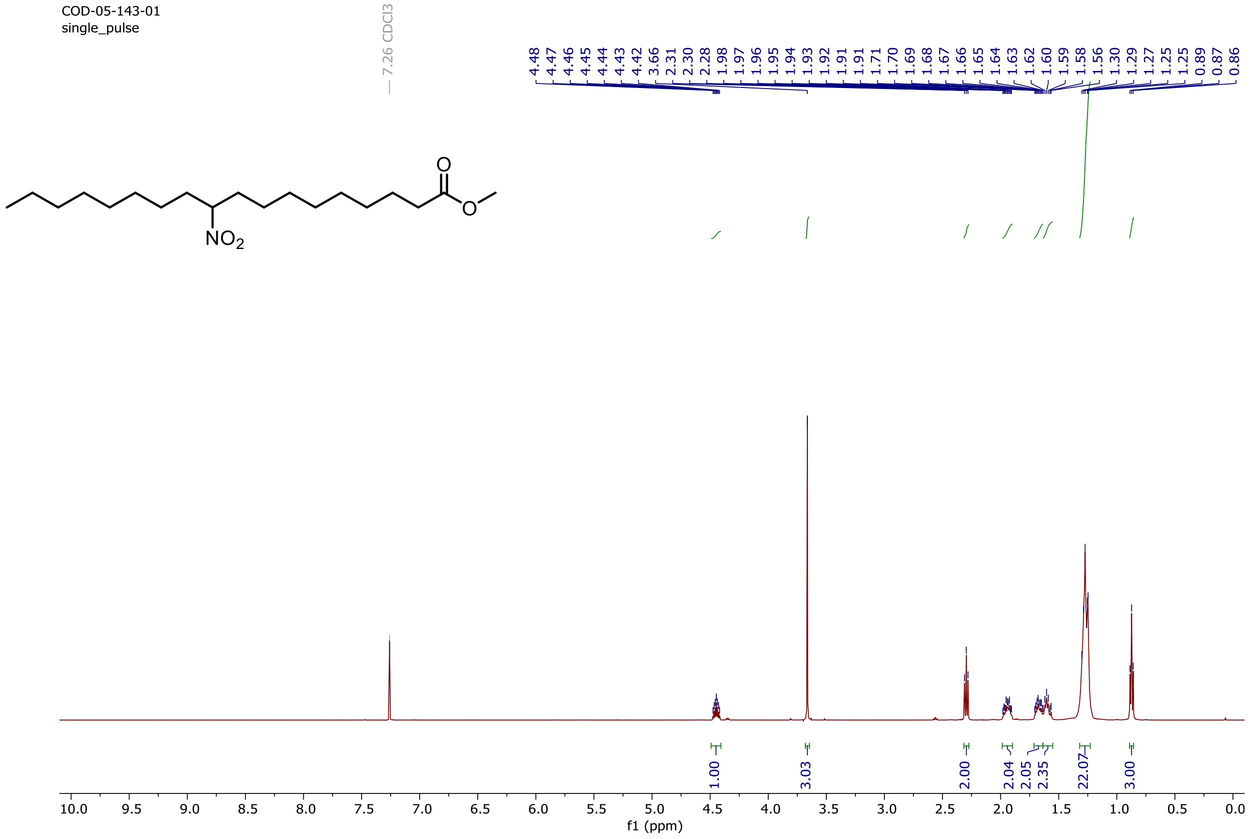

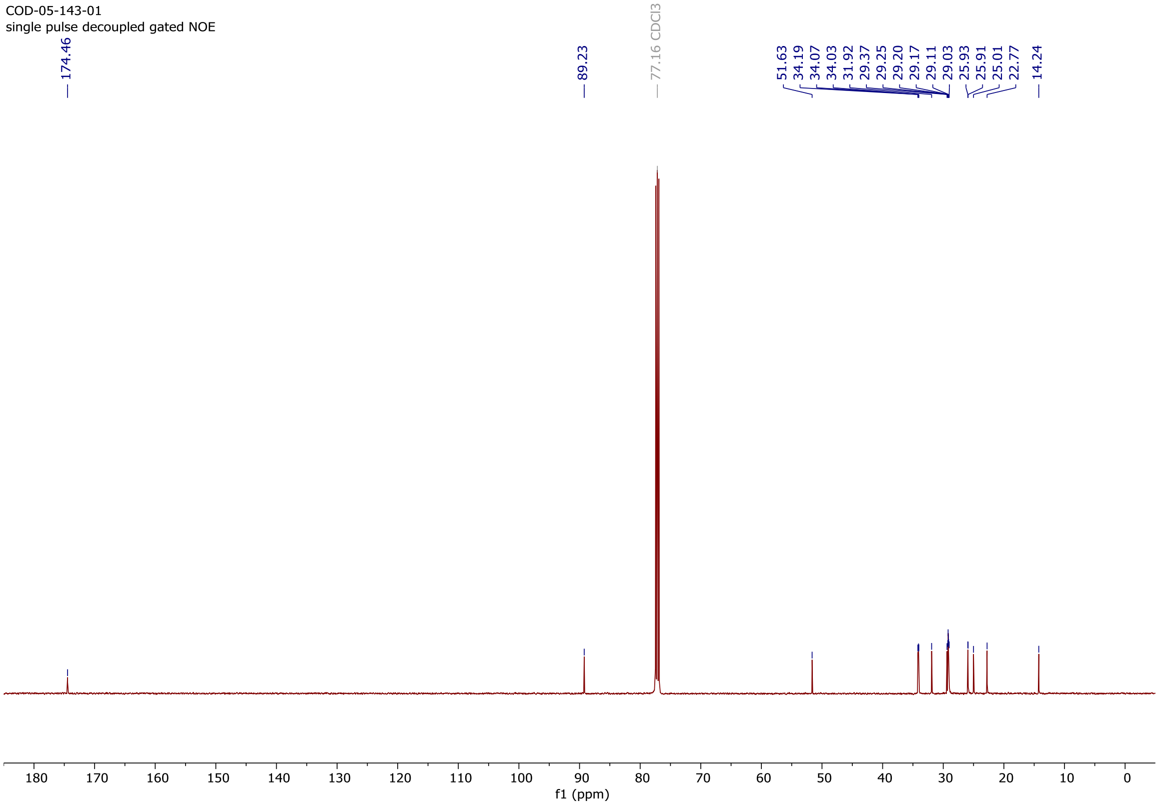

Copy of ^{1}H and ^{13}C{^{1}H} spectra of **10-nitrostearic acid (10-NO_2SA)**

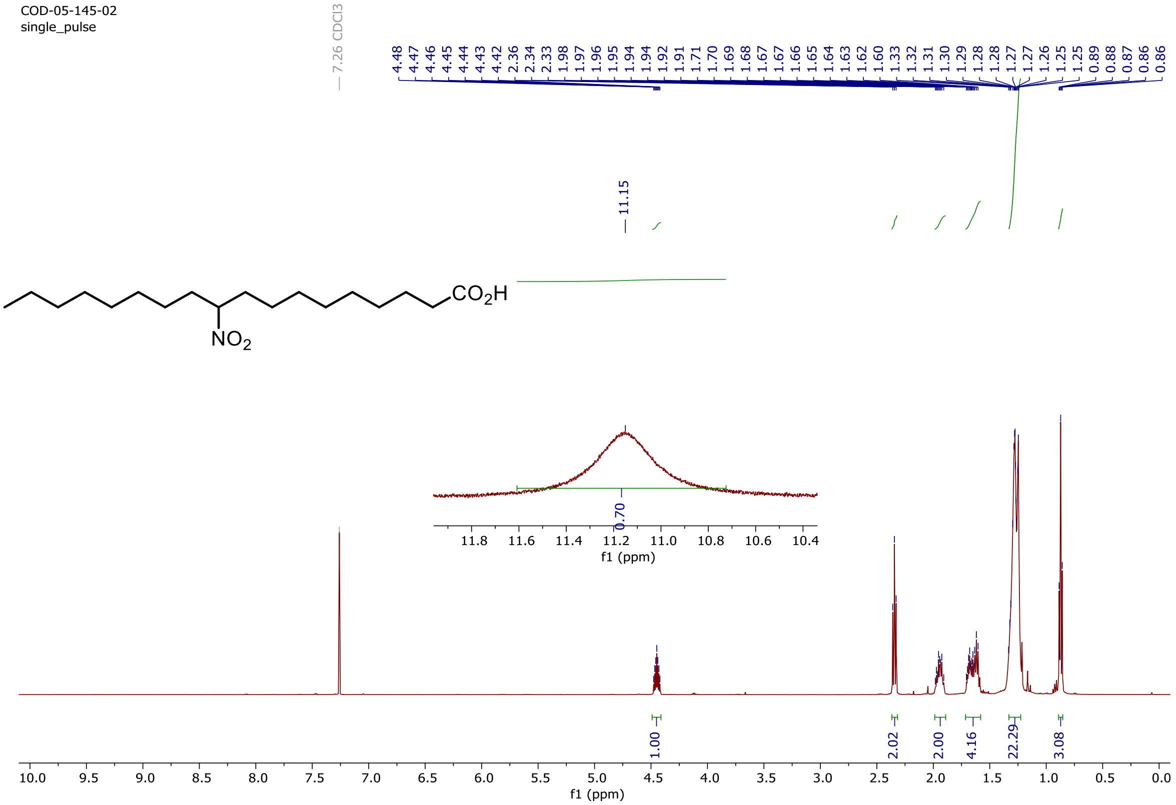

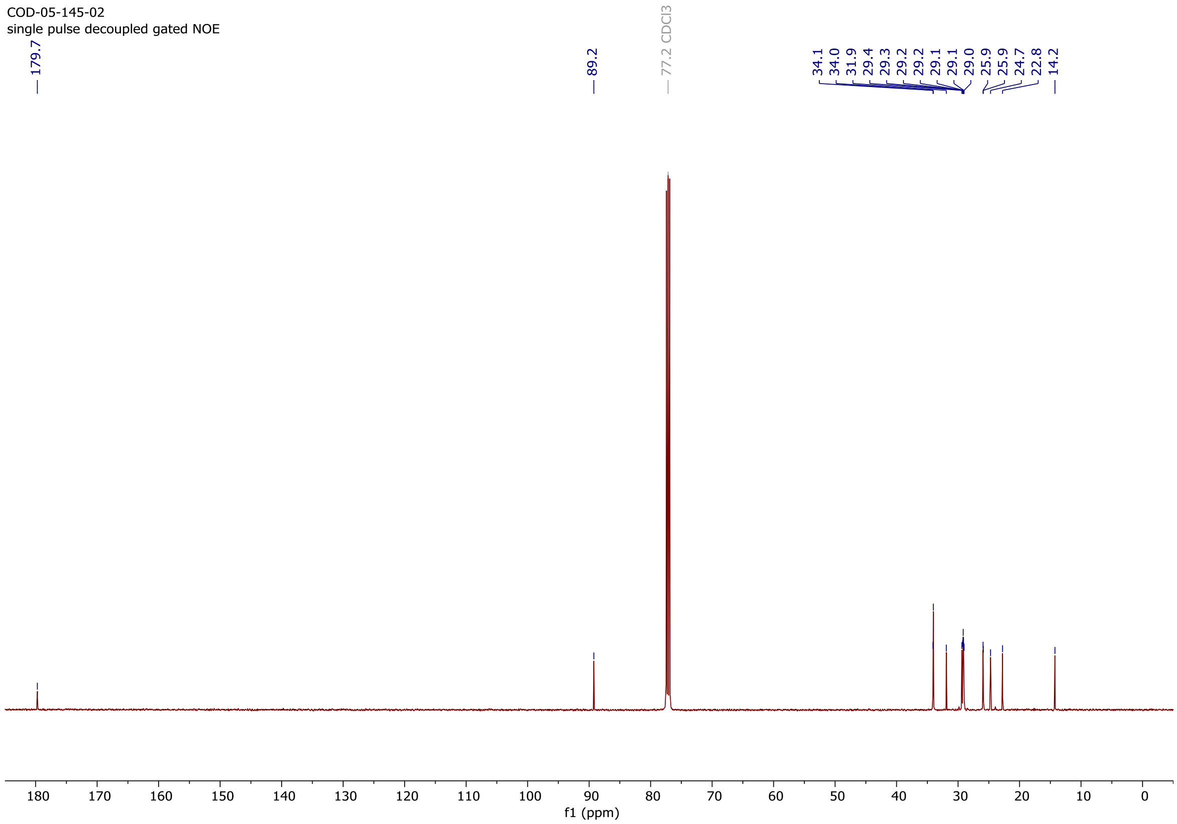

HPLC traces for lead compounds

HPLC chromatogram of **10-NO_2LA**

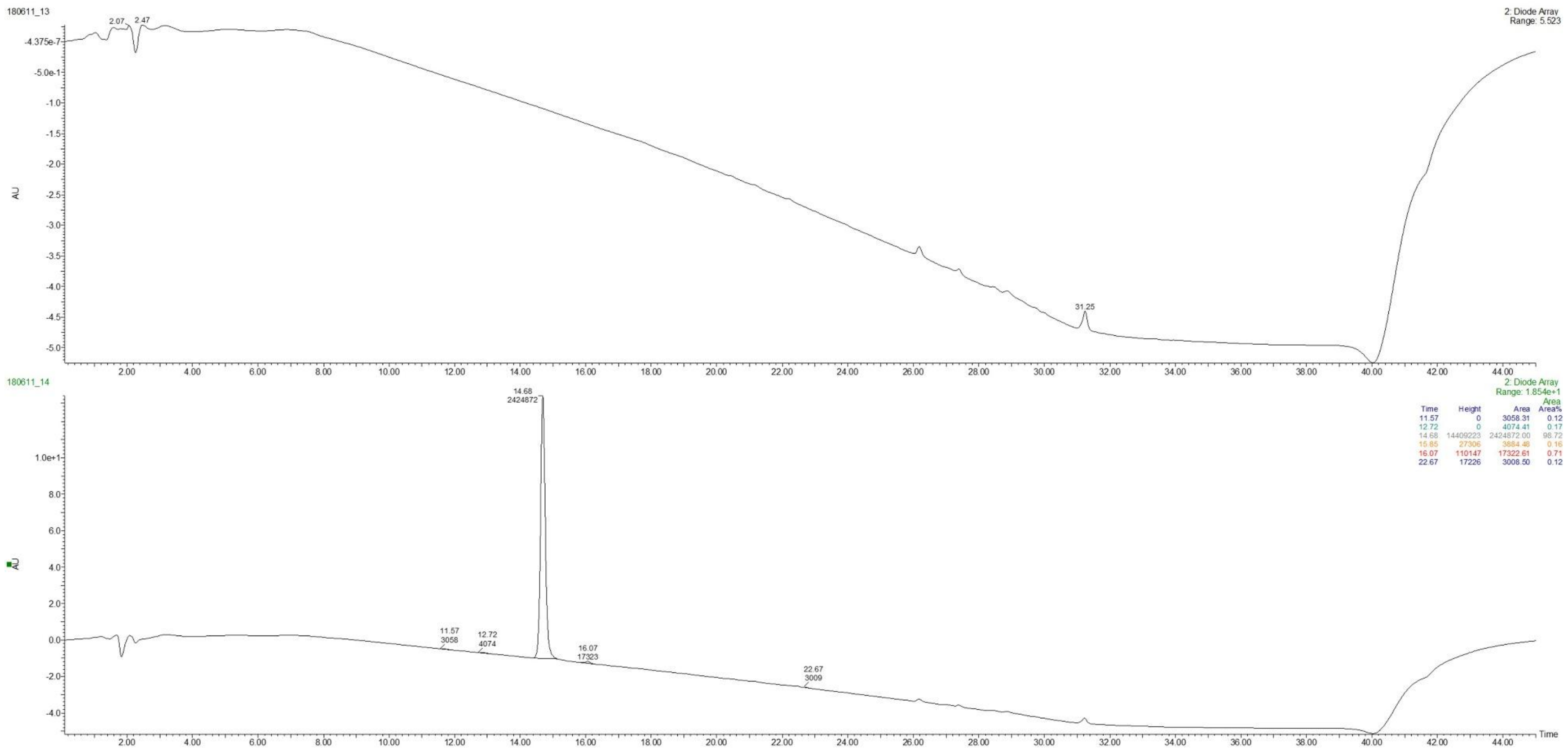

HPLC chromatogram of **10-NO_2OA**

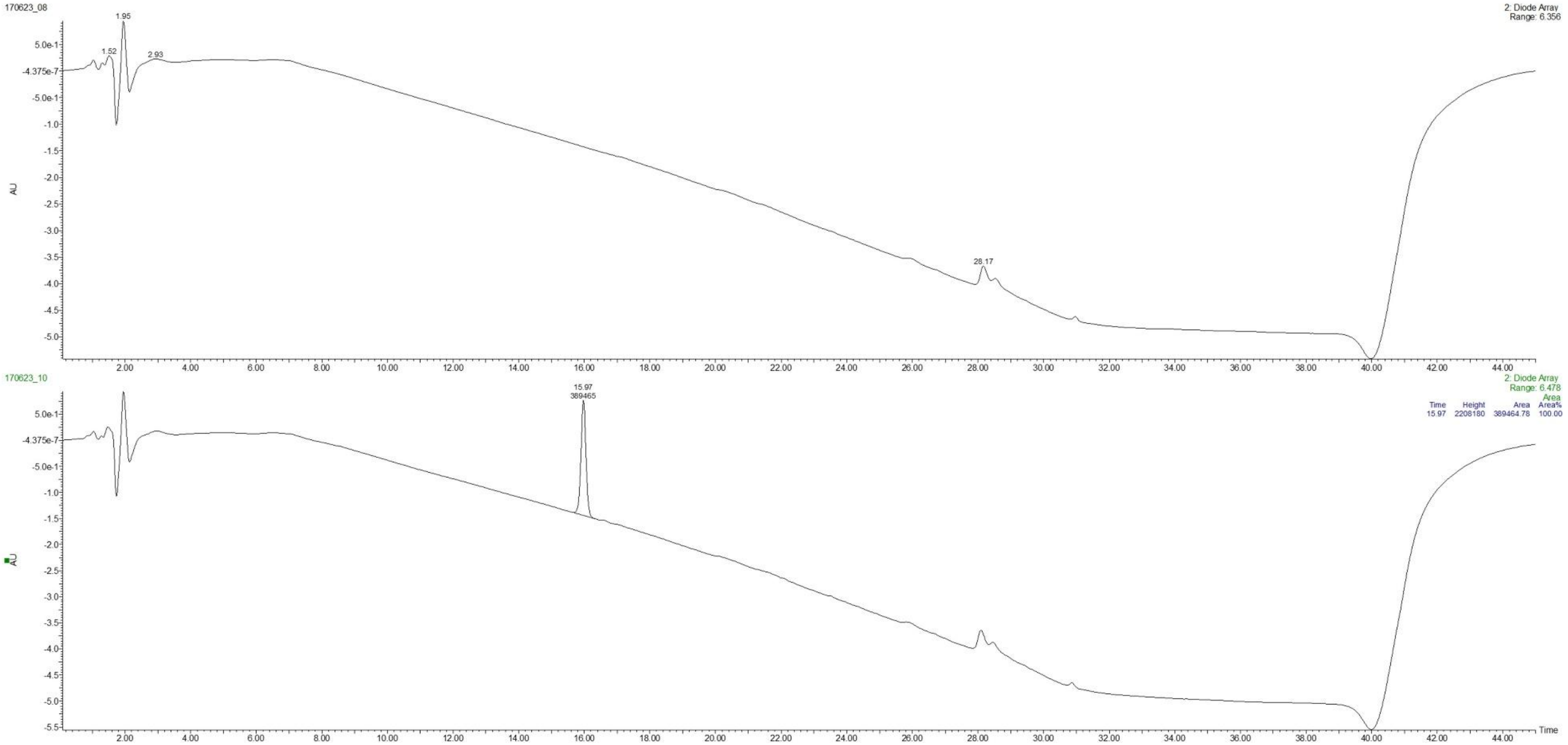

HPLC chromatogram of **9-NO_2OA**

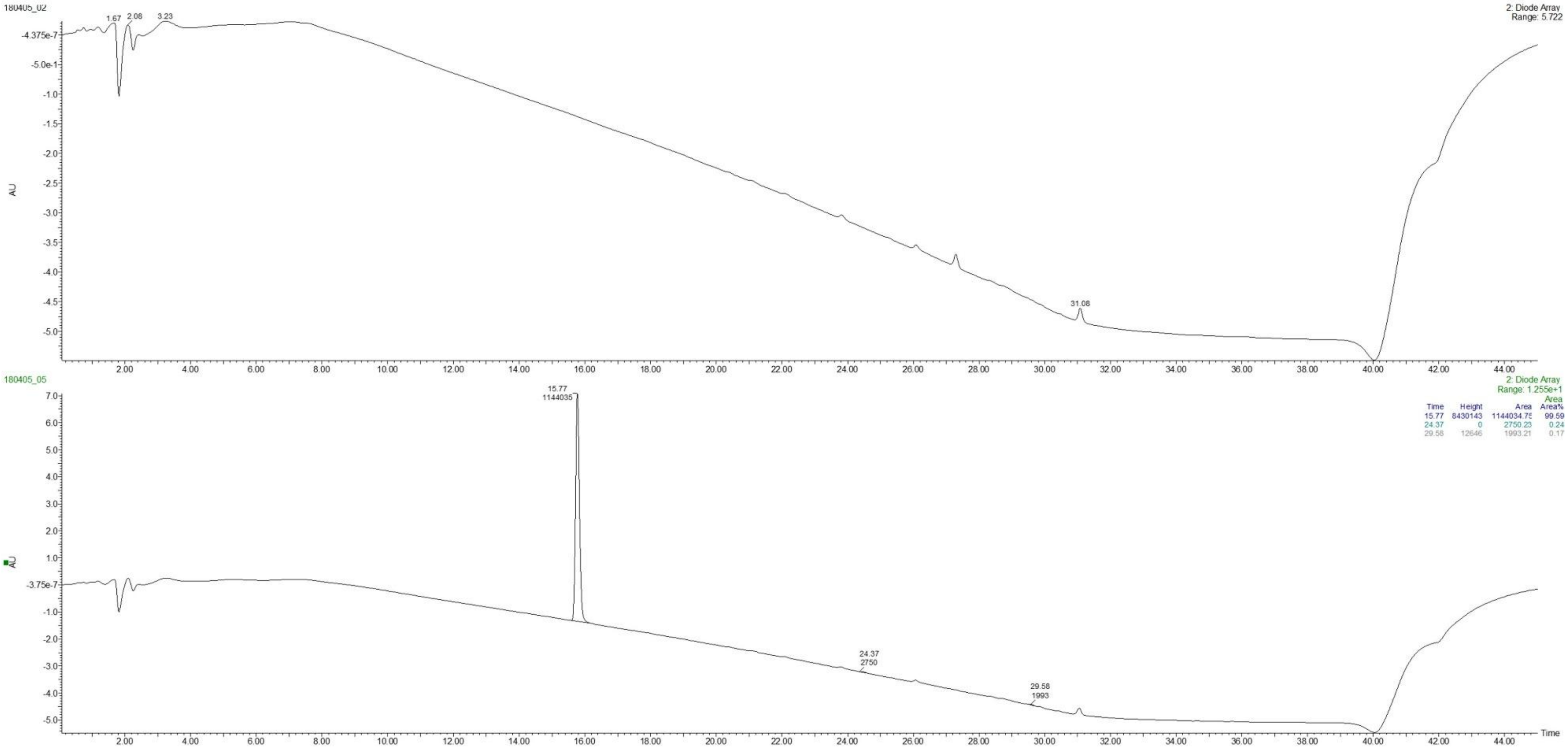

HPLC chromatogram of **9-NO_2cLA**

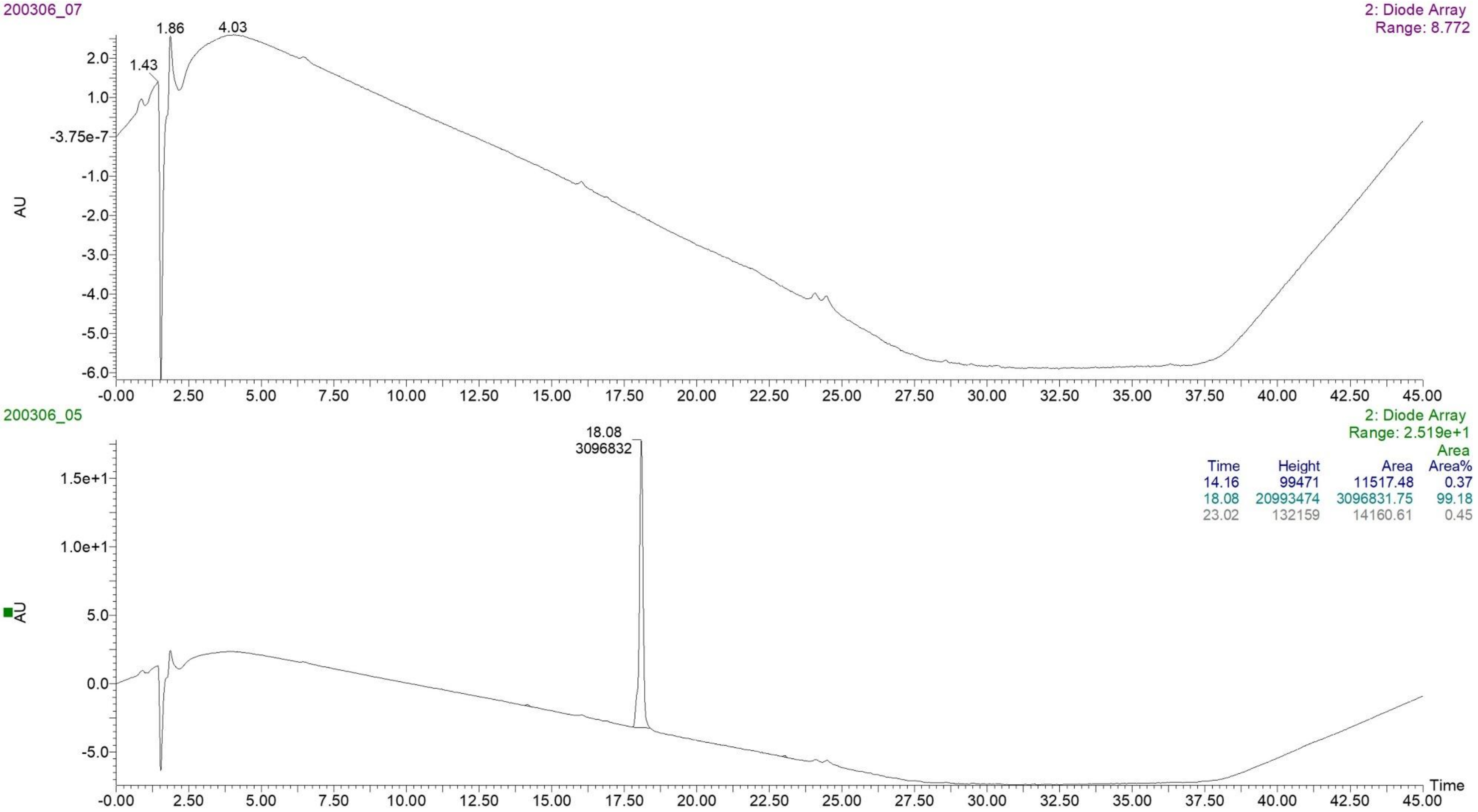

HPLC chromatogram of **10-NO_2SA**

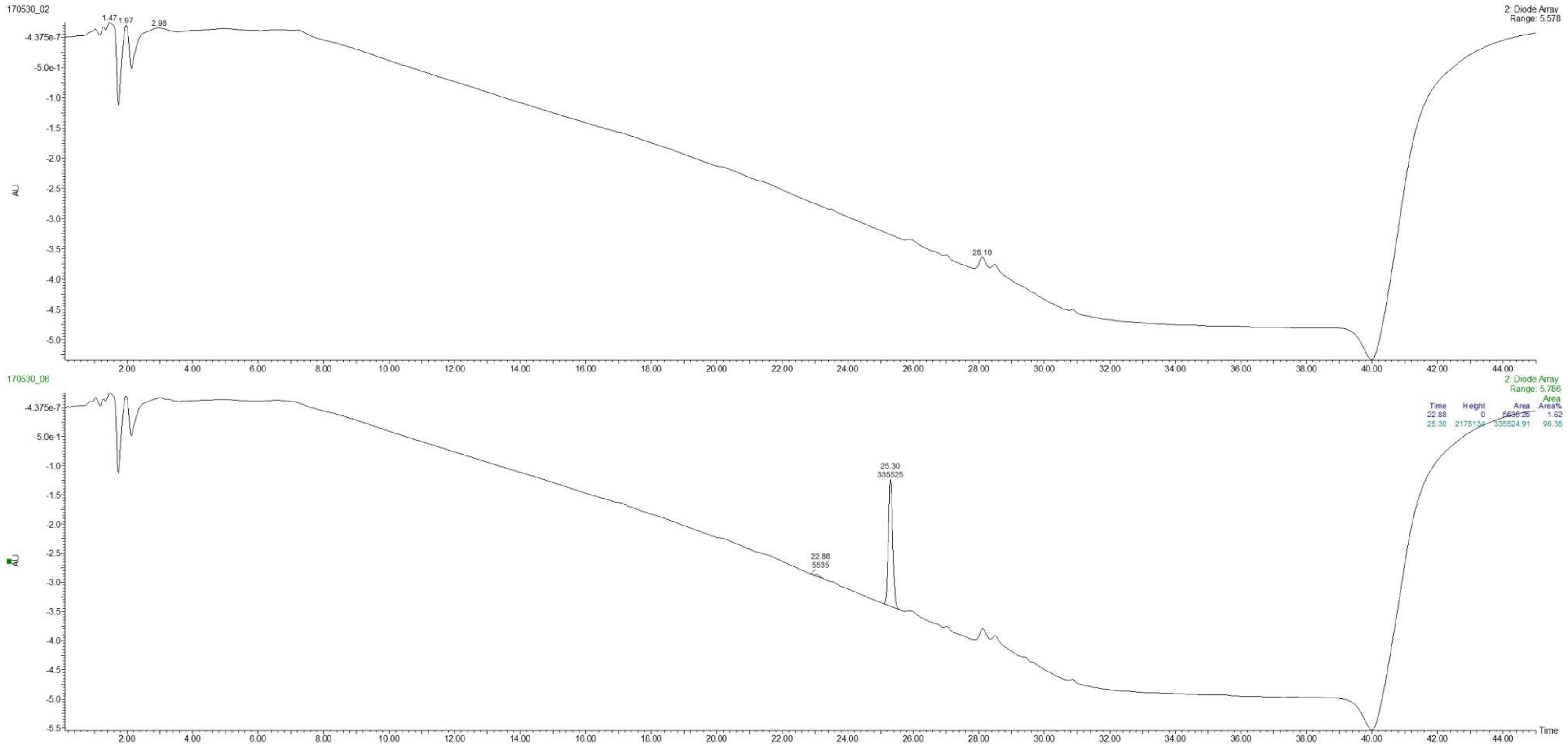

Supplement: Supplementary file 1 [file jm5c00982_si_001.pdf]
